# An energy decomposition and extrapolation scheme for evaluating electron transfer rate constants: a case study on electron self-exchange reactions of transition metal complexes†

**DOI:** 10.1039/d3ra05784d

**Published:** 2023-11-01

**Authors:** Akihiro Mutsuji, Kenichiro Saita, Satoshi Maeda

**Affiliations:** a Graduate School of Chemical Sciences and Engineering, Hokkaido University Sapporo Hokkaido 060-8628 Japan; b Department of Chemistry, Graduate School of Science, Hokkaido University Sapporo Hokkaido 060-0810 Japan smaeda@eis.hokudai.ac.jp; c Institute for Chemical Reaction Design and Discovery (WPI-ICReDD), Hokkaido University Sapporo Hokkaido 001-0021 Japan; d ERATO Maeda Artificial Intelligence for Chemical Reaction Design and Discovery Project, Hokkaido University Sapporo Hokkaido 060-0810 Japan; e Research and Services Division of Materials Data and Integrated System (MaDIS), National Institute for Materials Science (NIMS) Tsukuba Ibaraki 305-0044 Japan

## Abstract

A simple approach to the analysis of electron transfer (ET) reactions based on energy decomposition and extrapolation schemes is proposed. The present energy decomposition and extrapolation-based electron localization (EDEEL) method represents the diabatic energies for the initial and final states using the adiabatic energies of the donor and acceptor species and their complex. A scheme for the efficient estimation of ET rate constants is also proposed. EDEEL is semi-quantitative by directly evaluating the seam-of-crossing region of two diabatic potentials. In a numerical test, EDEEL successfully provided ET rate constants for electron self-exchange reactions of thirteen transition metal complexes with reasonable accuracy. In addition, its energy decomposition and extrapolation schemes provide all the energy values required for activation-strain model (ASM) analysis. The ASM analysis using EDEEL provided rational interpretations of the variation of the ET rate constants as a function of the transition metal complexes. These results suggest that EDEEL is useful for efficiently evaluating ET rate constants and obtaining a rational understanding of their magnitudes.

## Abstract 2

A simple approach to the analysis of electron transfer (ET) reactions based on energy decomposition and extrapolation schemes is proposed.

## Graphical abstract

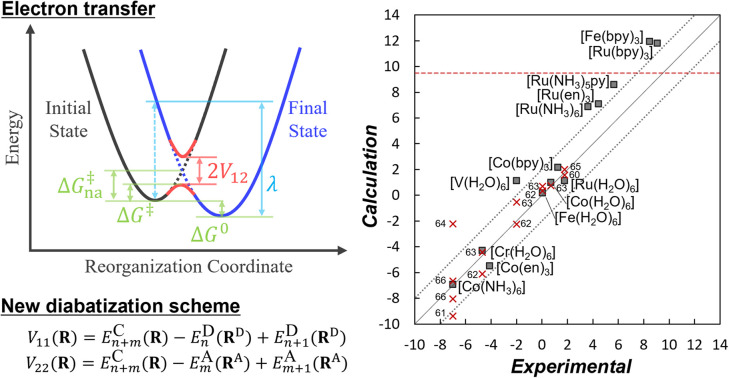

## Introduction

Electron transfer (ET) plays a fundamental role in various chemical systems, including artificial photosynthesis, photocatalysis, electrocatalysis, and electronic devices.^1–6^ Theoretical tools are needed to elucidate and design novel reactivities and functions triggered by ET.^7–10^ The ET rate constant is given by eqn (1).^9,11,12^1
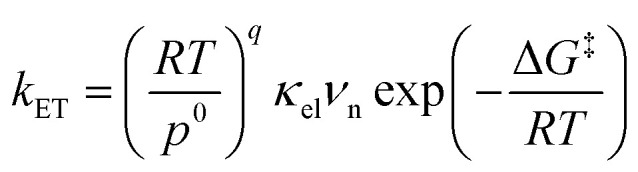
where *R* is the gas constant, *T* is the temperature, *p*^0^ is the standard atmosphere, *q* is either 1 or 0 for intermolecular or intramolecular ET, *κ*_el_ is the electronic transmission coefficient, *ν*_n_ is an effective nuclear frequency along the reaction coordinate, and Δ*G*^‡^ is the Gibbs energy of activation on the adiabatic potential energy surface (PES). *κ*_el_ is given by eqn (2)–(4).2
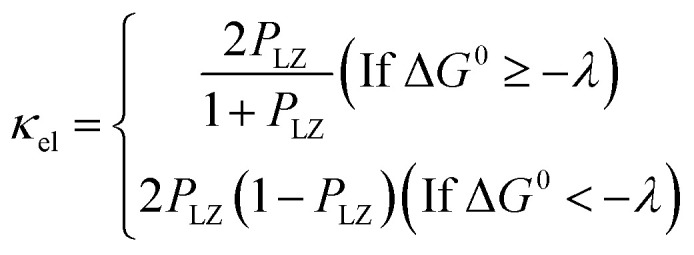

3 *P*_LZ_ = 1 − exp(−2π*γ*)

**Fig. 1:**
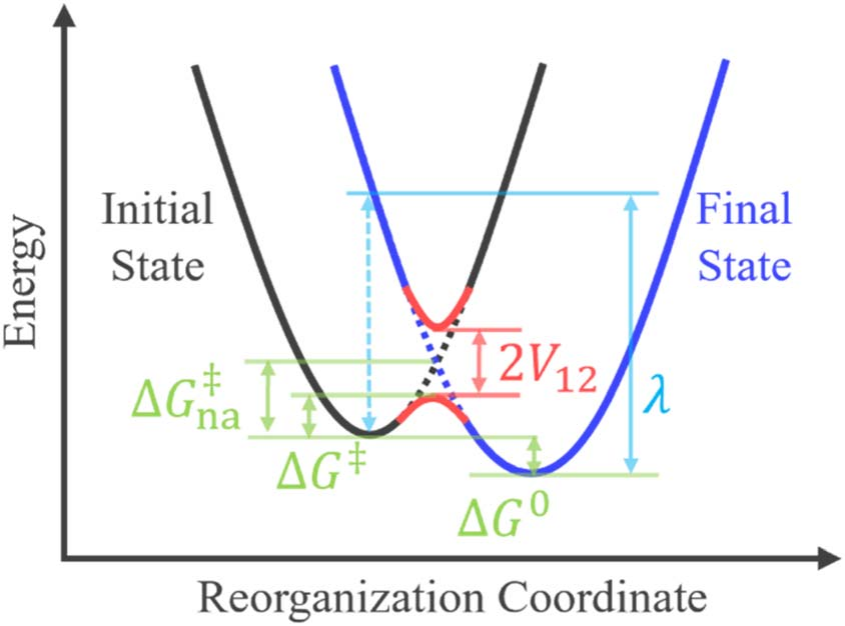
Crossing of two diabatic potential energy surfaces and the relations among *V*_12_, *λ*, Δ*G*^0^, Δ*G*^‡^, and Δ*G*^‡^_na_ in the rate constant expressions.

4
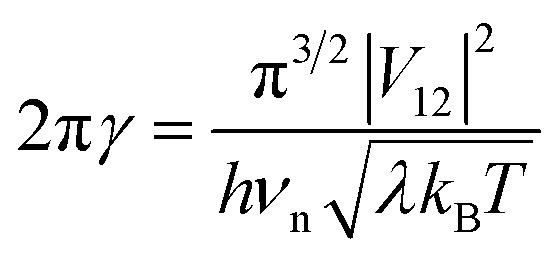
where *P*_LZ_ is the Landau–Zener transition probability, *k*_B_ is the Boltzmann constant, *h* is the Planck constant, *V*_12_ is the coupling constant, and *λ* is the reorganization energy (Fig. 1). When *V*_12_ is relatively small and 2πγ ≪ 1, the Marcus theory equation^13^eqn (5) is obtained from Taylor series of eqn (3).5
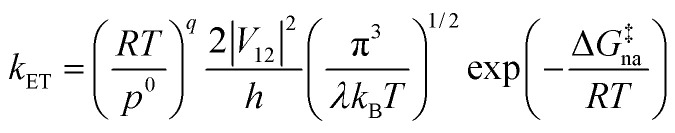


The harmonic approximation is often used to describe the diabatic PESs of the initial and final states, which allows one to estimate the Gibbs energy of activation Δ*G*^‡^_na_ (subscript “na” means “non-adiabatic”) by the following eqn (6) without explicitly locating the intersection between the two diabatic PESs on the multidimensional coordinate space. Note that the reaction barrier in eqn (1) is Δ*G*^‡^, not Δ*G*^‡^_na_ (Fig. 1).6
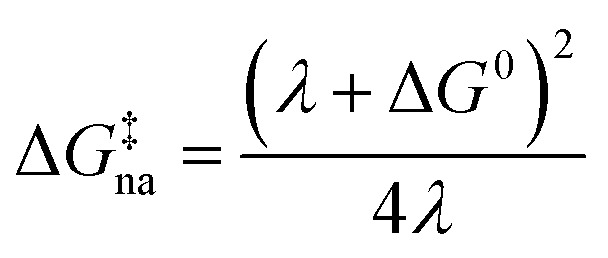


Since the diabatization required to obtain the diabatic PESs and their coupling *V*_12_ is not unique, different approaches have been proposed depending on the purpose.^14–23^ For example, the generalized Mulliken–Hush (GMH) theory is for ET and generates diabatic states based on the assumption that the initial and final diabatic states are charge-localized states at different centers of the donor and acceptor.^24,25^ Constrained density functional theory (CDFT) is also useful for ET and forms diabatic states by imposing constraints on the partial charge (or spin) of arbitrary molecules or fragments using the Lagrange multiplier.^26,27^

To go beyond the framework based on the harmonic approximation, it is necessary to consider the anharmonic effect on the reaction coordinate. This requires explicit evaluations of PESs at around the non-adiabatic transition takes place. Molecular dynamics simulations can be performed to obtain accurate transition probabilities by calculating a large number of trajectories passing through such a region.^9,28–31^ A computationally less demanding alternative approach is to identify the seam-of-crossing (SX) or conical intersection (CI) geometry within the full-dimensional coordinate space.^32–38^ Simulations of non-adiabatic events based on SX or CI geometries have become increasingly common in recent years.^39–45^ In this approach, Δ*G*^‡^_na_ is estimated as the energy gap between the SX/CI geometry and the initial state equilibrium (EQ) geometry.

This study presents a semi-quantitative yet efficient algorithm for predicting the ET rate constant based eqn (1)–(4) rather than eqn (5) because our scheme tends to give relatively large coupling values as discussed in Results and discussion. The present method directly identifies the SX geometry. A simple diabatization based on energy decomposition and extrapolation schemes^46–48^ allows one to optimize an SX geometry and evaluate Δ*G*^‡^, Δ*G*^‡^_na_, *V*_12_, and *λ*. Rate constants for thirteen electron self-exchange reactions were calculated and compared with experimental^49–59^ and calculated^60–66^ values reported in the literature to investigate the performance of the present method. The calculated rate constants showed a good correlation with the experimental values. The energy decomposition scheme provided interpretations of the factors determining the magnitudes of the ET rate constants based on the activation strain model (ASM).^67–69^

## Method

In this study, a simple diabatization scheme called energy decomposition and extrapolation-based electron localization (EDEEL) is proposed. In EDEEL, the diabatic energies for the initial and final states *V*_11_ and *V*_22_ are given by eqn (7) and (8), respectively.

7 *V*_11_(**R**) = *E*^C^_*n*+*m*_(**R**) − *E*^D^_*n*_(**R**^D^) + E^D^_*n*+1_(**R**^D^)

8 *V*_22_(**R**) = *E*^C^_*n*+*m*_(**R**) − *E*^A^_*m*_(**R**^A^) + *E*^A^_*m*+1_(**R**^A^)

where **R** is the geometry of the donor–acceptor complex system, and **R**^D^ and **R**^A^ are its donor and acceptor components, respectively. The subscript for each *E* corresponds to the number of electrons in the corresponding system (*n* = *m* in the electron self-exchange reactions). *E*^C^_*n*+*m*_(**R**) is the adiabatic energy of the donor–acceptor complex without the moving electron. *E*^D^_*n*_(**R**) and *E*^D^_*n*+1_(**R**) are the adiabatic energies of the donor species without and with the moving electron, respectively. *E*^A^_*m*_(**R**) and *E*^A^_*m*+1_(**R**) are the adiabatic energies of the acceptor species without and with the moving electron, respectively. This scheme assumes that the moving electron is localized on either the donor or acceptor species and does not interact with the corresponding counterpart. In other words, this scheme sets these assumptions as the conditions for diabatization. In the EDEEL scheme, an SX geometry between *V*_11_ and *V*_22_ is the critical point at which *k*_ET_ is evaluated.

Δ*G*^‡^_na_ = (*G*_SX_ − *G*_initial−EQ_) is the Gibbs energy gap between the SX and the initial state EQ geometries. Gibbs energy corrections at the EQ and SX are calculated by the normal mode analysis. The coupling *V*_12_ is computed as the energy difference *V*_12_ = *V*_11_−*E*^C^_*n*+*m*+1_, where *E*^C^_*n*+*m*+1_ is the lower adiabatic energy between those for the two states that contain the contributions of the two diabatic states the most. Thus, at the SX between *V*_11_ and *V*_22_, *i.e.*, when *V*_11_ = *V*_22_, the EDEEL scheme reproduces the diabatic–adiabatic relation of the two-state model in eqn (9).9
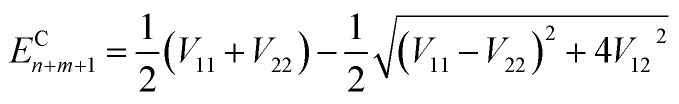


In the electron self-exchange reactions, *E*^C^_*n*+*m*+1_ is the adiabatic energy of the donor–acceptor complex in its electronic ground state. The parameter *λ* is obtained as eqn (10) based on eqn (6).10
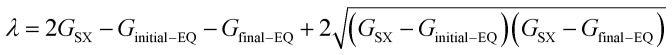
where relations Δ*G*^‡^_na_ = *G*_SX_ − *G*_initial−EQ_ and Δ*G*^0^ = *G*_final−EQ_ − *G*_initial−EQ_ are used, *G*_final−EQ_ is Gibbs energy of the final state EQ, and eqn (6) gives *λ* = 4Δ*G*^‡^_na_ for electron self-exchange reactions.^70^

The distance dependence of the ET reaction rate may have a distinct peak.^60^ This study regards adiabatic ground state at the SX geometry between *V*_11_ and *V*_22_ as the approximate transition state and maximizes the ET rate constant *k*_ET_ within the SX region to obtain the final *k*_ET_ (=*k*_calc_). This is done by taking the donor–acceptor distance *r*_DA_ (the metal–metal distance in the transition metal examples below) as the reaction coordinate, evaluating the *k*_ET_ at various *r*_DA_, and maximizing the *k*_ET_ along *r*_DA_. Details of how the initial *r*_DA_(ini) is systematically determined and how the *k*_ET_ is maximized are described in the ESI.†

**Fig. 2:**
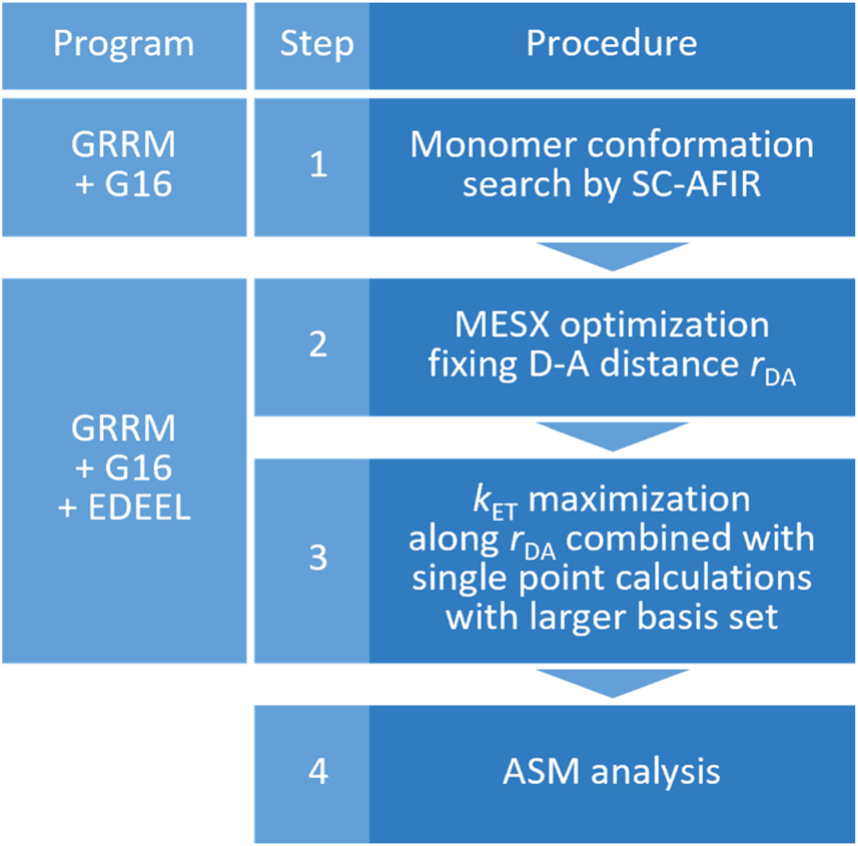
The entire workflow for obtaining an electron transfer rate constant by EDEEL and the calculation programs used in each procedure.

As shown in Fig. 2, the entire *k*_calc_ evaluation workflow consists of the following four steps.

(1) Systematic search for monomer conformations using the SC-AFIR^71,72^ method implemented in the Global Reaction Route Mapping (GRRM) program.^73^

(2) Optimization of a minimum energy SX (MESX) geometry^74,75^ within the SX hypersurface between *V*_11_ and *V*_22_ while maintaining the donor–acceptor distance to *r*_DA_(ini).

(3) *k*_ET_ maximization along *r*_DA_ by calculating *k*_ET_ for five *r*_DA_ values near *r*_DA_(ini), where line search is done by simple quadratic curve fitting using three *k*_ET_ values in this study. In the calculation of *k*_ET_ with eqn (1)–(4), the Gibbs energies are extrapolated by the electronic energies obtained from single point calculations with larger basis set.

(4) ASM analysis at the geometry of the largest *k*_ET_ (=*k*_calc_) obtained in step 3 (optional).

## Results and discussion

The workflow in Fig. 2 was applied to the electron self-exchange reactions of thirteen transition metal complexes listed in Table 1. Electronic structure calculations were performed with the Gaussian 16 program^76^ and geometry optimization was performed with the GRRM program at the UωB97X-D/Def2-SV(P)^77,78^ level taking into account the solvent effect of water by the conductor-like polarizable continuum model (C-PCM).,^79,80^ where UωB97X-D stands for a spin unrestricted DFT calculation using the ωB97X-D functional. *k*_ET_ was maximized using the electronic energy calculated at the UωB97X-D/Def2-TZVP^81^ level and the Gibbs energy correction at the UωB97X-D/Def2-SV(P) level; the calculation level is represented as UωB97X-D/Def2-TZVP//Def2-SV(P). In the calculation of *k*_ET_, *ν*_n_ in eqn (1) was approximated as *k*_B_*T*/*h*, the coefficient of transition state theory rate constant equation. The spin multiplicity was chosen to stabilize each state as much as possible and was set to the values listed in Table 1. The reaction set in Table 1 covers reactions of different timescales with rate constants in the wide range of 10^−7^ to 10^9^ dm^3^ mol^−1^ s^−1^, and thus would be suitable as a test set. It was assumed that both +3 and +2 charged complexes were in the electronic ground state at SX, although there were possibilities for ET *via* metal-to-ligand-charge transfer (MLCT) or electronic excited states of each complex.^82^ Further computational details are presented in Computational section.

**Table 1:** Spin multiplicity used in the calculations, experimental rate constants *k*_expt_ of the thirteen electron self-exchange reactions of transition metal complexes, and their data sources

Redox couple	Spin multiplicity (3+/2+)	Experimental values		References
		*k*_expt_/dm^3^ mol^−1^ s^−1^	log_10_ *k*_expt_	
[V(H_2_O)_6_]^3+/2+^	3/4	3 × 10^−3^, 1 × 10^−2^	−2.0	49 and 50
[Cr(H_2_O)_6_]^3+/2+^	4/5	<2 × 10^−5^	−4.7	51
[Fe(H_2_O)_6_]^3+/2+^	6/5	1.1	0.0	51
[Co(H_2_O)_6_]^3+/2+^	5/4	5	0.7	49 and 52
[Ru(H_2_O)_6_]^3+/2+^	2/1	(6 ± 4) × 10^1^	1.8	53
[Co(NH_3_)_6_]^3+/2+^	1/4	>10^−7^	−7.0	51
[Ru(NH_3_)_6_]^3+/2+^	2/1	4.3 × 10^3^	3.6	49 and 54
[Ru(NH_3_)_5_py]^3+/2+^	2/1	4.7 × 10^5^	5.7	55
[Co(en)_3_]^3+/2+^	1/4	7.7 × 10^−5^	−4.1	56
[Ru(en)_3_]^3+/2+^	2/1	2.8 × 10^4^	4.4	51
[Fe(bpy)_3_]^3+/2+^	2/1	3 × 10^8^	8.5	57 and 58
[Co(bpy)_3_]^3+/2+^	1/4 (1/2)^a^	18	1.3	51
[Ru(bpy)_3_]^3+/2+^	2/1	1.2 × 10^9^	9.1	59

a Values for monomer and SX are shown outside and inside the parentheses, respectively.

**Fig. 3:**
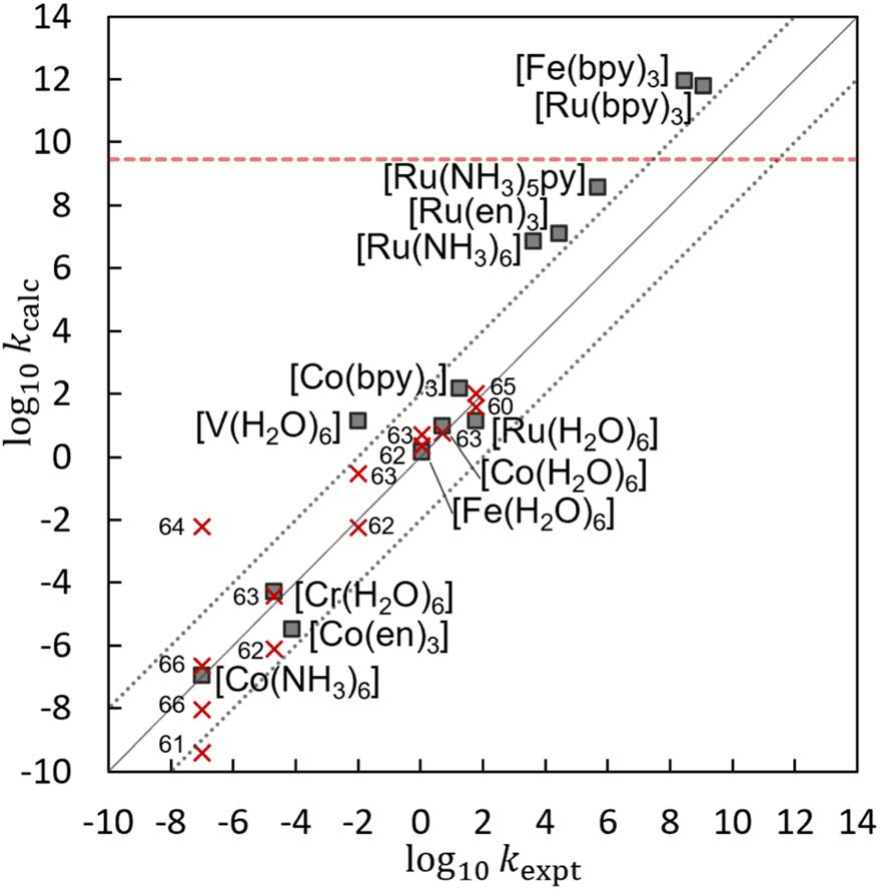
A correlation between experimental log_10_ *k*_expt_ and calculated log_10_ *k*_calc_ by EDEEL at the UωB97X-D/Def2-TZVP//Def2-SV(P) level (filled squares) or by the other method^60–66^ (red crosses). The red dotted line represents the diffusion-controlled rate constant.^49^

Table 2 lists the log_10_ *k*_calc_, Δ*G*^‡^, *λ*, *V*_12_, *κ*_el_, and *r*_DA_ values obtained by the present calculations at the UωB97X-D/Def2-TZVP//Def2-SV(P) level. Fig. 3 shows the correlation between the calculated log_10_ *k*_calc_ and the experimental log_10_ *k*_expt_,^49–59^ and that the present calculation reproduces the experimental trend well. Fig. 3 also compares the log_10_ *k*_calc_ values with those obtained by the other theoretical methods.^60–66^ Despite the simplicity of the algorithm, which does not include a complex diabatization scheme or any empirical factors, the present method provides an accuracy comparable to the other theoretical methods, making EDEEL promising for the semi-quantitative estimation of ET rate constants.

**Table 2:** log_10_ *k*_calc_, Gibbs energies of activation Δ*G*^‡^, reorganization energy *λ*, diabatic coupling *V*_12_, electronic transmission coefficient *κ*_el_, and donor–acceptor distance *r*_DA_ by EDEEL at the UωB97X-D/Def2-TZVP//Def2-SV(P) level

Redox couple	log_10_ *k*_calc_	Δ*G*^‡^/eV	*λ*/eV	*V*_12_/eV	*κ*_el_	*r*_DA_/Å
[V(H_2_O)_6_]^3+/2+^	1.1	0.77	4.02	0.23	1.00	5.76
[Cr(H_2_O)_6_]^3+/2+^	−4.3	1.09	5.45	0.27	1.00	5.74
[Fe(H_2_O)_6_]^3+/2+^	0.2	0.83	4.29	0.24	1.00	5.72
[Co(H_2_O)_6_]^3+/2+^	1.0	0.78	4.10	0.24	1.00	5.53
[Ru(H_2_O)_6_]^3+/2+^	1.1	0.77	4.01	0.23	1.00	5.78
[Co(NH_3_)_6_]^3+/2+^	−6.9	1.25	5.26	0.07	0.96	7.68
[Ru(NH_3_)_6_]^3+/2+^	6.9	0.43	2.01	0.07	0.99	7.39
[Ru(NH_3_)_5_py]^3+/2+^	8.6	0.33	1.65	0.08	1.00	9.51
[Co(en)_3_]^3+/2+^	−5.5	1.16	4.86	0.05	0.91	7.96
[Ru(en)_3_]^3+/2+^	7.1	0.42	1.90	0.06	0.98	8.02
[Fe(bpy)_3_]^3+/2+^	11.9	0.08	0.87	0.13	1.00	9.08
[Co(bpy)_3_]^3+/2+^	2.2	0.71	3.24	0.10	1.00	8.75
[Ru(bpy)_3_]^3+/2+^	11.8	0.14	0.97	0.10	1.00	8.91

In Table 2, the coupling values are relatively large, making *κ*_el_ almost unity. This is because our diabatization constraint assumes that there is no interaction between the electron to be transferred and the acceptor molecule. In other words, our constraint pushes the strong attraction between the negative charge on the moving electron and the +3 positive charge on the acceptor molecule into the coupling value, making the coupling values large. As for the non-adiabaticity, electron transfer reactions in [M(H_2_O)_6_]^3+/2+^, which showed the largest coupling values among the systems in our calculation, are known as non-adiabatic process with smaller actual coupling values (<100 cm^−1^).^83^ The log_10_ *k*_calc_ does not include the diffusion-controlled rate constant, which is estimated to be *k*_diff_ = 3 × 10^9^ dm^3^ mol^−1^ s^−1^ for these reactions.^49^ In other words, *k*_diff_ would be a source of the large error seen for fast reactions such as [Fe(bpy)_3_]^3+/2+^ and [Ru(bpy)_3_]^3+/2+^. In our scheme, the C-PCM environments of the initial and final states are relaxed independently, which does not satisfy the Franck–Condon principle. This would have led to the underestimation of reaction barriers. For example, the outer-sphere reorganization energy of [Ru(H_2_O)_6_]^3+/2+^ was estimated to be 0.68 eV based on dielectric continuum theory.^13^ This implies additional reaction barrier of 0.17 eV, reducing its log_10_ *k*_ET_ from 1.1 to −1.7, which is three-order of magnitude smaller than the experimental value. The simple addition of the outers-sphere reorganization energy correction is not sufficient in our scheme. The better treatment of outer-sphere reorganization in our scheme needs further study in the future. Many other factors such as explicit solvation, dynamic and quantum motion of the atoms, and higher order (or static) electron correlation would contribute to the error. However, further improvement of the accuracy considering these factors is beyond the scope of this study.

Thanks to the energy decomposition and extrapolation scheme of EDEEL, the ASM analysis^67–69^ can be performed using the energy components, *i.e.*, *E*^C^_*n*+*m*_, *E*^D^_*n*_, *E*^D^_*n*+1_, *E*^A^_*m*_, and *E*^A^_*m*+1_, calculated during the EDEEL calculations, without any additional calculations. Note that the ASM analysis was performed using electronic energies rather than Gibbs energies for the SX geometry with the highest *k*_ET_ value. In the ASM/EDEEL analysis, the strain energies in donor Δ*E*^‡^_Strain(D)_ and acceptor Δ*E*^‡^_Strain(A)_ and their interaction energy Δ*E*^‡^_Interaction(C)_ are represented as follows.

11 Δ*E*^‡^_Strain(D)_ = *E*^D(SX)^_*n*+1_ − *E*^D(initial−EQ)^_*n*+1_

12 Δ*E*^‡^_Strain(A)_ = *E*^A(SX)^_m_ − *E*^A(initial−EQ)^_m_

**Fig. 4:**
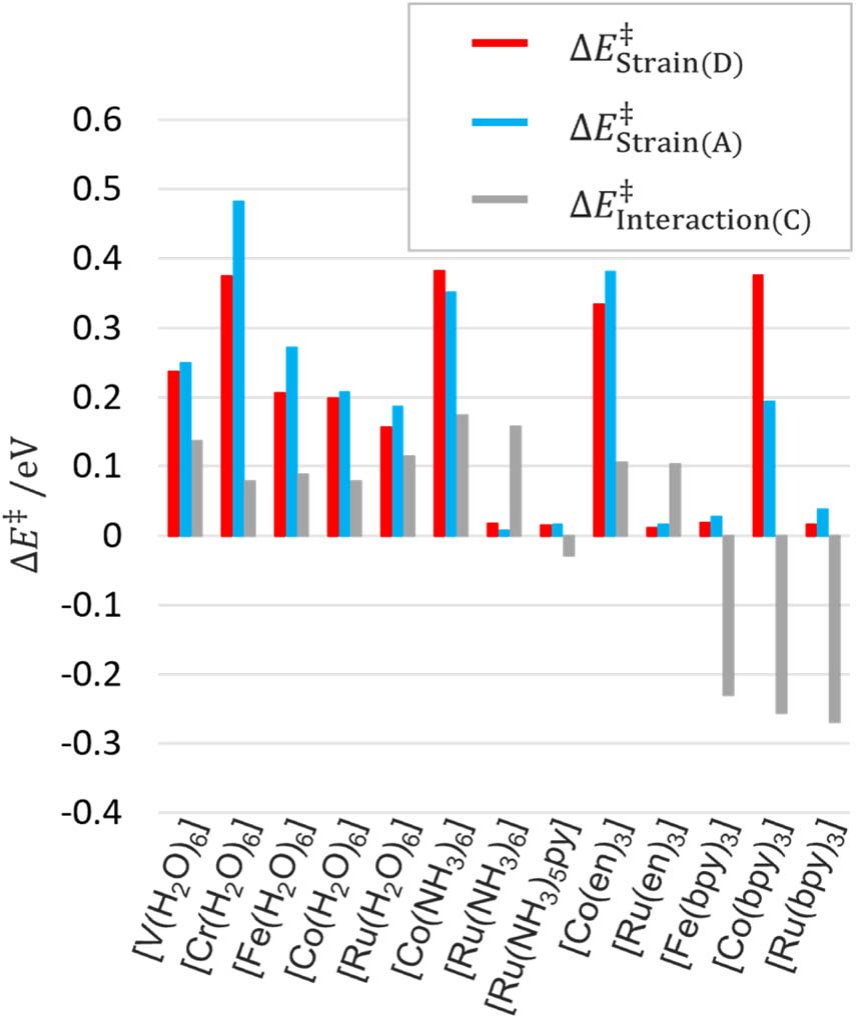
Strain energies and interaction energies by ASM analysis at UωB97X-D/Def2-TZVP.

**Fig. 5:**
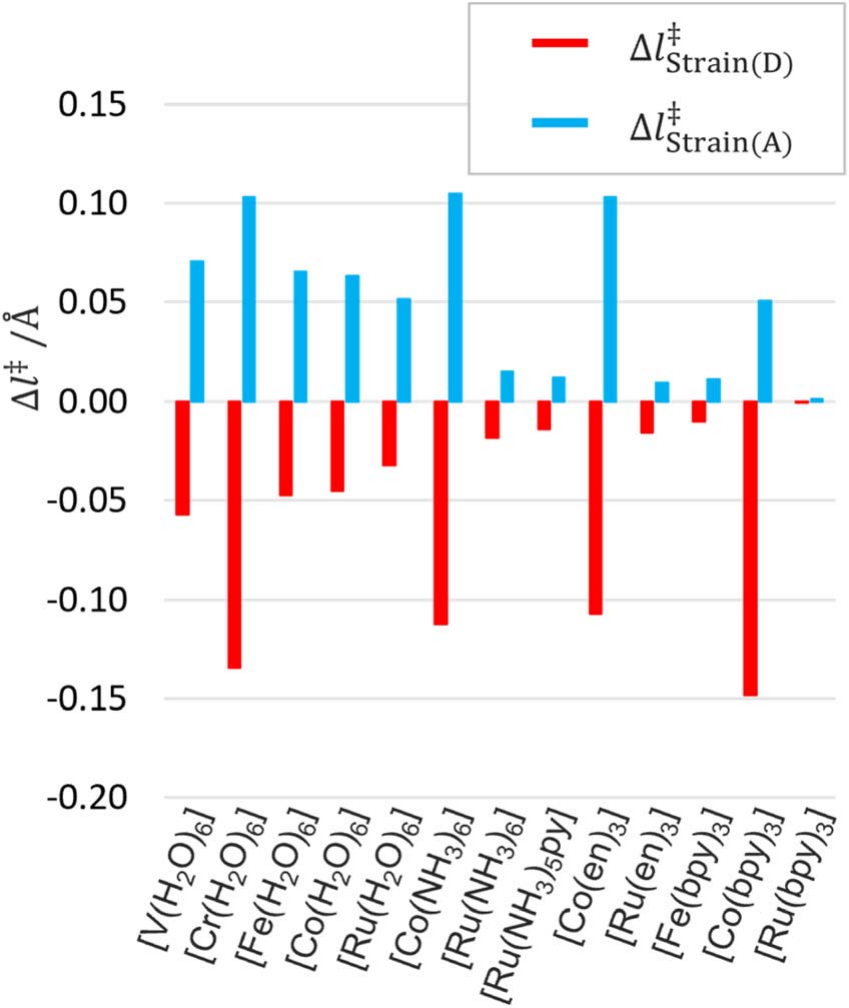
Changes in mean coordination bond length.

13 Δ*E*^‡^_Interaction(C)_ = *E*^C(SX)^_*n*+*m*_ − *E*^D(SX)^_*n*_ − *E*^A(SX)^_*m*_

where *E*^D(SX)^_*n*+1_, *E*^D(SX)^_*n*_, *E*^A(SX)^_*m*_ and *E*^C(SX)^_*n*+*m*_ are the electronic energies at the SX geometry of the donor with the moving electron, that of the donor without the moving electron, that of the acceptor without the moving electron, and that of the donor–acceptor complex without the moving electron, and *E*^D(initial−EQ)^_*n*+1_ and *E*^A(initial−EQ)^_*m*_ are the electronic energies at the initial state EQ geometry of the donor with the moving electron and of the acceptor without the moving electron. The donor and acceptor in the initial EQ geometry are assumed to be infinitely far apart and not interacting. Fig. 4 and 5 show the results of the ASM/EDEEL analysis at the UωB97X-D/Def2-TZVP level. The magnitudes of the strain energies of the aqua and cobalt complexes are large as shown in Fig. 4, reflecting the large oxidation/redox potentials of their +2/+3 species. In these systems, to compensate for the large energy gaps between the +2 and +3 species, large structural deformations (Δ*l*^‡^_Strain(D)_ and Δ*l*^‡^_Strain(A)_, changes in mean coordination bond length in donor and acceptor, respectively) are seen in the SXs around their central metal, as shown in Fig. 5. In pyridine and bipyridine complexes, attractive interactions are seen due to π-stacking between pyridine moieties and π-stacking between bipyridine molecules, respectively.^13,49^ On the other hand, aqua, ammonia, and ethylenediamine complexes show repulsive interactions, even though aqua complexes formed hydrogen bonding between water molecules. These interpretations by ASM/EDEEL would be beneficial in understanding ET rate constants quantitatively.

## Conclusion

In this article, we have proposed a simple diabatization scheme called EDEEL for the analysis of ET reactions. EDEEL represents the diabatic energies, *V*_11_ and *V*_22_, by combining the adiabatic energies of the donor, acceptor, and their complex. Such a simple representation allows us to easily optimize the SX geometries between the two diabatic potentials. A scheme for estimating the ET rate constants at the SX geometries has also been introduced. The diabatic coupling *V*_12_ is also estimated using the adiabatic energy of the donor–acceptor complex. In other words, the present scheme allows one to obtain all the parameters necessary to estimate ET rate constants using only the adiabatic energies. Numerical tests with electron self-exchange reactions of thirteen transition metal complexes have shown that EDEEL reproduces the trend of the experimental rate constants well and is semi-quantitative.

Two advantages of using EDEEL can be suggested. One is that EDEEL can be combined with any *ab initio* method and program without touching their codes. This is because all diabatic potential elements, *i.e.*, *V*_11_, *V*_22_ and *V*_12_, are represented by the adiabatic energies. The other is that EDEEL provides all the energy components and SX geometries needed in the ASM analysis. The latter is helpful in interpreting the ET efficiency and further designing a system with higher ET efficiency. In the present applications, the EDEEL-based ASM analysis successfully provided rational explanations for the variation of the magnitudes of the ET rate constants depending on the transition metal complexes.

## Computational section

The above procedures were implemented in an in-house Python script as an interface program between the GRRM program and the Gaussian 16 program.^73,76^ The script takes a geometry from GRRM, performs the necessary electronic structure calculations to obtain the EDEEL PES at the geometry using Gaussian 16, and returns the energy and gradient (and Hessian if necessary) of the EDEEL PES at the geometry to GRRM. Geometry optimizations were performed by a developer version of the GRRM program at the UωB97X-D/Def2-SV(P) level. The “Stable = Opt” option was also used to identify the electronic ground state configuration of a given spin multiplicity. The “Int(Grid = 99 590)” option was used in DFT calculations. Although some of the aqua-complexes are highly acidic and may not prevail as simple [M(H_2_O)_6_]^3+/2+^ complexes in actual aqueous solution, the metal centers were assumed to be hexa-coordinated in this study as previous reports.^60,62,63,65^ Solvent water was modeled by the conductor-like polarizable continuum model (C-PCM).^79,80^ Gibbs energy corrections were obtained from the standard normal mode analysis of 3*N*−8 dimensions (one direction orthogonal to SX and one direction along the vector between two metal atoms were removed from the full 3*N*−6 dimensions) at *T* = 298.15 K and *p*^0^ = 1 atm, with normal mode frequencies smaller than 100 cm^−1^ replaced by 100 cm^−1^ as suggested in the literature.^84^ The bases of Gibbs activation energies were calculated as the sum of the extrapolated Gibbs energies of the most stable donor and acceptor monomers, where the most stable structures were identified by a systematic conformation search using SC-AFIR with possible spin multiplicities. An entropy correction −4.3 kcal mol^−1^ was added to the Gibbs energy of the dimer complex in the activation energy calculation to account for the restriction of their mobility in the water solvent, following the previous studies.^85–87^ The rate constant maximization was done at the UωB97X-D/Def2-TZVP//Def2-SV(P) level.

## Conflicts of interest

There are no conflicts to declare.

## Supplementary Material

RA-013-D3RA05784D-s001
