# Supplementary material for: An energy decomposition and extrapolation scheme for evaluating electron transfer rate constants: a case study on electron self-exchange reactions of transition metal complexes
Source: RSC Adv. 2023 Nov 1;13(46):32097–103. doi: 10.1039/d3ra05784d (PMC10619204; doi:10.1039/d3ra05784d)
Supplement: RA-013-D3RA05784D-s001 [file RA-013-D3RA05784D-s001.pdf]

Electronic Supplementary Material (ESI) for RSC Advances.
This journal is © The Royal Society of Chemistry 2023

Electronic supplementary information:

An energy decomposition and extrapolation scheme for evaluating electron transfer rate constants: A case study on electron self-exchange reactions of transition metal complexes

Akihiro Mutsuji[1], Kenichiro Saita[2], and Satoshi Maeda*[2,3,4,5]

[1]Graduate School of Chemical Sciences and Engineering, Hokkaido University; Sapporo Hokkaido 060-8628, Japan
[2]Department of Chemistry, Graduate School of Science, Hokkaido University; Sapporo, Hokkaido 060-0810, Japan
[3]Institute for Chemical Reaction Design and Discovery (WPI-ICReDD), Hokkaido University; Sapporo, Hokkaido 001-0021, Japan
[4]ERATO Maeda Artificial Intelligence for Chemical Reaction Design and Discovery Project, Hokkaido University; Sapporo, Hokkaido 060-0810, Japan
[5]Research and Services Division of Materials Data and Integrated System (MaDIS), National Institute for Materials Science (NIMS); Tsukuba, Ibaraki 305-0044, Japan

Contents

1. Initial SX geometry preparation
2. Maximization of electron transfer reaction rate constants
3. Cartesian coordinates and energies of the most stable monomer equilibrium geometries in Gibbs energy with charge 3+ and 2+ at the UωB97X-D/Def2-TZVP//Def2-SV(P) level
4. Cartesian coordinates and energies of SX geometries obtained from the initial SX geometry generation at the UωB97X-D/Def2-SV(P) level
5. Cartesian coordinates and energies of MESX geometries by EDEEL with metal-metal distance fixed to r_{DA}(ini) at the UωB97X-D/Def2-SV(P) level
6. Cartesian coordinates and energies of MESX geometries with metal-metal distances showing maximum electron self-exchange reaction rate constants at the UωB97X-D/Def2-TZVP//Def2-SV(P) level

1. Initial SX geometry preparation

The initial SX structure for the subsequent rate constant maximization along the metal-metal distance was generated as a MESX geometry between the following E'_1 and E'_2,

$$E_1^{'} = V_{11} + \Delta E_1^{MM} \#(S1)$$

$$E_2^{'} = V_{22} + \Delta E_2^{MM} \#(S2)$$

where the molecular mechanics (MM) correction terms are expressed as follows,

$$\Delta E_1^{MM} = \left(E_{n+m+1}^{\mathrm{C}\text{ - }MM\text{ - }Initial} - E_{n+1}^{D\text{ - }MM} - E_{m}^{A\text{ - }MM}\right) - \left(E_{n+m}^{\mathrm{C}\text{ - }MM} - E_{n}^{D\text{ - }MM} - E_{m}^{A\text{ - }MM}\right)\#(S3)$$

$$\Delta E_2^{MM} = \left(E_{n+m+1}^{\mathrm{C}\text{ - }MM\text{ - Final}} - E_{n}^{D\text{ - }MM} - E_{m+1}^{A\text{ - }MM}\right) - \left(E_{n+m}^{\mathrm{C}\text{ - }MM} - E_{n}^{D\text{ - }MM} - E_{m}^{A\text{ - }MM}\right)\#(S4)$$

where n and m, respectively, are the number of electrons in the donor and acceptor species ($n = m$ in the electron self-exchange reactions), the subscript for each E corresponds to the number of electrons in the corresponding system, $E_{n+m+1}^{\mathrm{C}\text{ - }MM\text{ - }Initial}$ and $E_{n+m+1}^{\mathrm{C}\text{ - }MM\text{ - Final}}$, respectively, are the MM energies of the donor-acceptor complex with the charge distributions of the initial and final states, and $E_{n}^{D\text{ - }MM}$, $E_{m}^{A\text{ - }MM}$, $E_{n+1}^{D\text{ - }MM}$, and $E_{m+1}^{A\text{ - }MM}$, respectively, are the MM energies of the donor and acceptor species. In other words, these MM terms represent the interaction between the moving electron and the corresponding counterpart, which is missing in V_{11} and V_{22}. An MESX geometry between E'_1 and E'_2 can be a good initial guess of the final geometry to compute k_{calc}. Therefore, the donor-acceptor distance at the MESX geometry between E'_1 and E'_2 is set as r_{DA}(ini). In the MM calculations, the universal force field[1] implemented in the Gaussian16 program was employed, where atomic charges were obtained by the DFT calculation with the keyword "pop=MKUFF".[2,3]

1 A. K. Rappe, C. J. Casewit, K. S. Colwell, W. A. Goddard and W. M. Skiff, *J. Am. Chem. Soc.*, 1992, **114**, 10024.

2 U. C. Singh and P. A. Kollman, *J. Comput. Chem.*, 1984, **5**, 129.

3 B. H. Besler, K. M. Merz Jr. and P. A. Kollman, *J. Comput. Chem.*, 1990, **11**, 431.

2. Maximization of electron transfer reaction rate constants

Starting from r_{DA}(ini), k_{ET} is maximized along the metal-metal distance r_{DA}, while k_{ET} is evaluated by the following equation.

$$k_{ET} = \left(\frac{RT}{p^0}\right)^q \kappa_{el} \nu_n exp\left(-\frac{\Delta G^\ddagger}{RT}\right) \#(1)$$

1. A scaling factor x is set to 1.0.
2. An increment d is set to 0.0125.
3. The iteration number n is set to 1.
4. A MESX is optimized while keeping r_{DA} at xr_{DA}(ini) by translation of the central metal atom in one complex. If x = 1.0 + (2a + 1)d (a = 0, 1, 2, …), "Int(Grid=FineGrid)" option for DFT calculation and "Opt=Loose" for geometry optimization were used. Else, "Int(Grid=99590)" option was used.
5. If x = 1.0 + (2a + 1)d (a = 0, 1, 2, …), add d to x and return to step 4. Else, perform the single point calculation with larger basis set at the MESX obtained in step4, and extrapolate the Gibbs energy of the MESX.
6. k_{ET} at this iteration $k_{ET}(n)$ is calculated with eqn (1) at the MESX obtained in step 4 using the extrapolated Gibbs energy obtained in step 5.
7. If not x = 1.1 nor n > 5, add d to x, increase n by 1, and return to step 4.
8. Calculate $k_{harmonic}$ as the maximum along a harmonic curve including the point with the largest $\log_{10}k_{ET}(m)$ and its two neighbors. If $|k_{harmonic} - k_{ET}(m)| < 0.1$, then set the final $k_{calc} = k_{ET}(m)$ and exit. Else, set x to the value of the maximum point along the harmonic curve, increase n by 1, and return to step 4.

Below, the actual $\log_{10}k_{ET}$ maximization processes for the thirteen transition metal complexes are presented in Fig. S1-S13.

Plots of $\log_{10}k_{ET}$ maximization at the UωB97X-D/Def2-TZVP//Def2-SV(P) level

$[V(H_2O)_6]^{3+/2+}$

Cycle 1 (Converged)

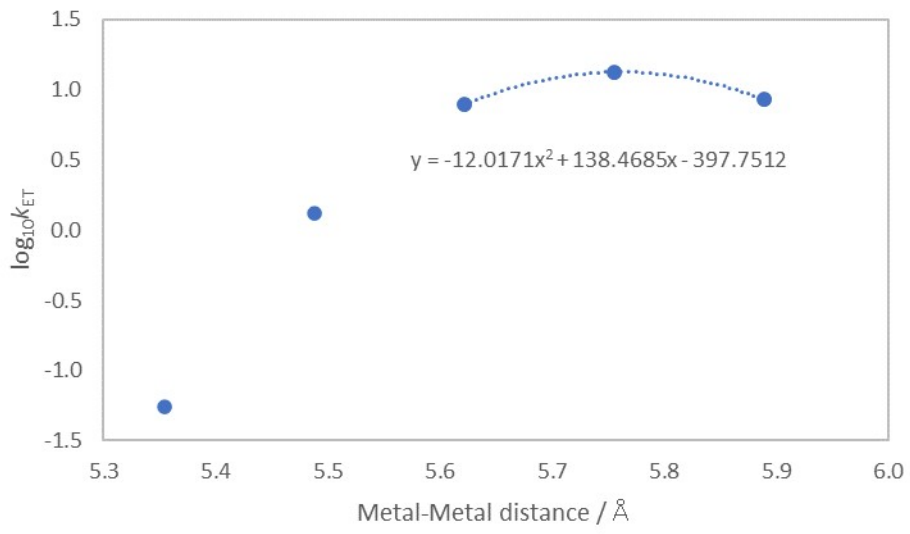

Fig. S1 Plots of $\log_{10}k_{ET}$ maximization for $[V(H_2O)_6]^{3+/2+}$ (Cycle 1).

$[Cr(H_2O)_6]^{3+/2+}$

Cycle 1 (Converged)

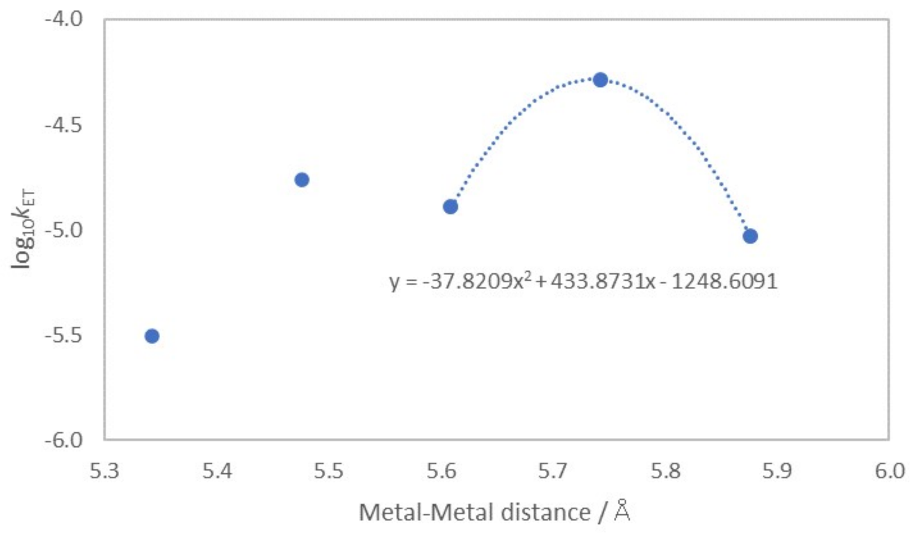

Fig. S2 Plots of $\log_{10}k_{ET}$ maximization for $[Cr(H_2O)_6]^{3+/2+}$ (Cycle 1).

$[Fe(H_2O)_6]^{3+/2+}$

Cycle 1 (Converged)

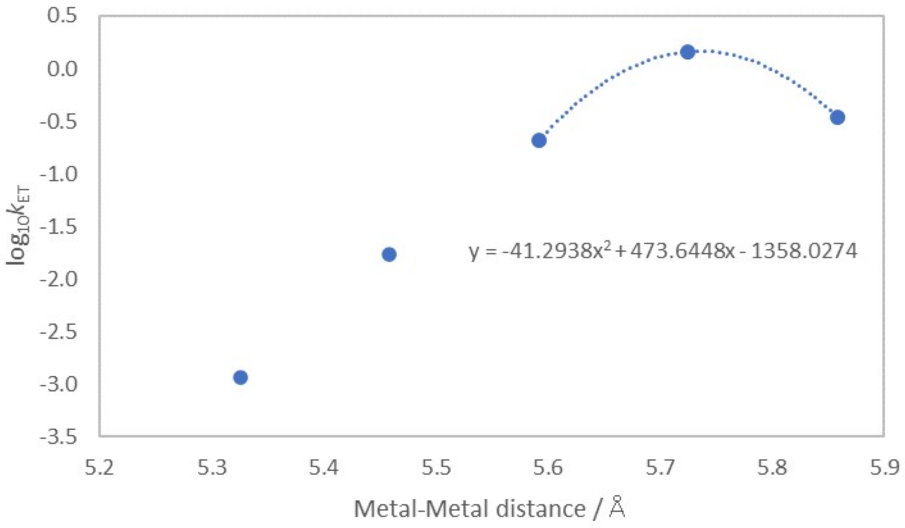

Fig. S3 Plots of $\log_{10}k_{ET}$ maximization for $[Fe(H_2O)_6]^{3+/2+}$ (Cycle 1).

$[Co(H_2O)_6]^{3+/2+}$

Cycle 1 (Converged)

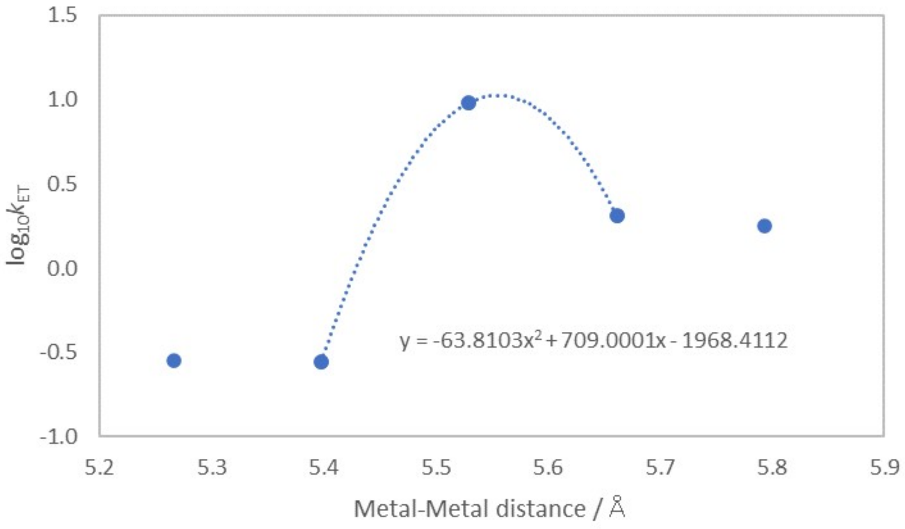

Fig. S4 Plots of $\log_{10}k_{ET}$ maximization for $[Co(H_2O)_6]^{3+/2+}$ (Cycle 1).

$[Ru(H_2O)_6]^{3+/2+}$

Cycle 1 (Not Converged)

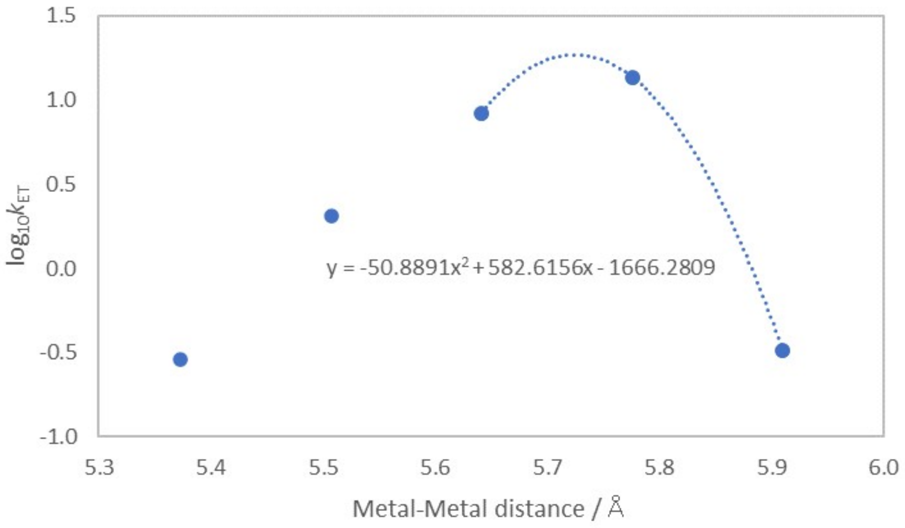

(a)

Cycle 2 (Converged)

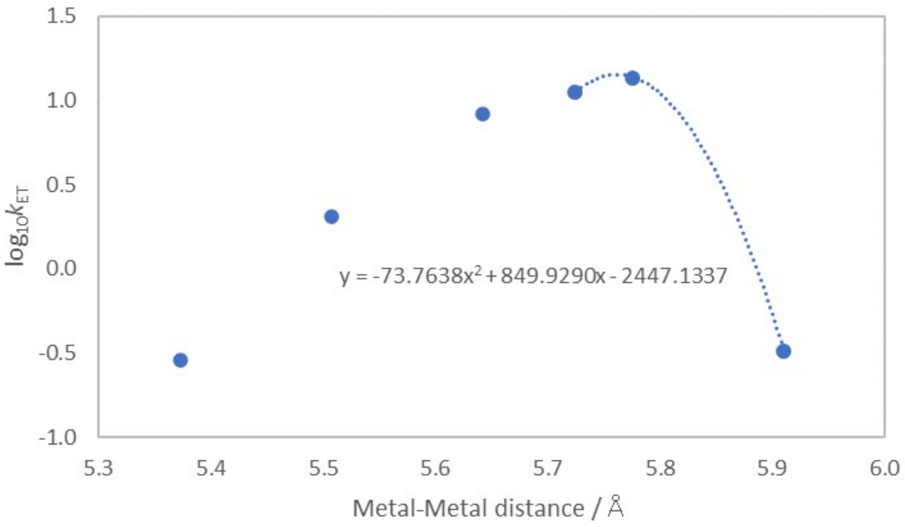

(b)

Fig. S5 Plots of $\log_{10}k_{ET}$ maximization for $[Ru(H_2O)_6]^{3+/2+}$. (a) Cycle 1, (b) Cycle2.

$[Co(NH_3)_6]^{3+/2+}$

Cycle 1 (Converged)

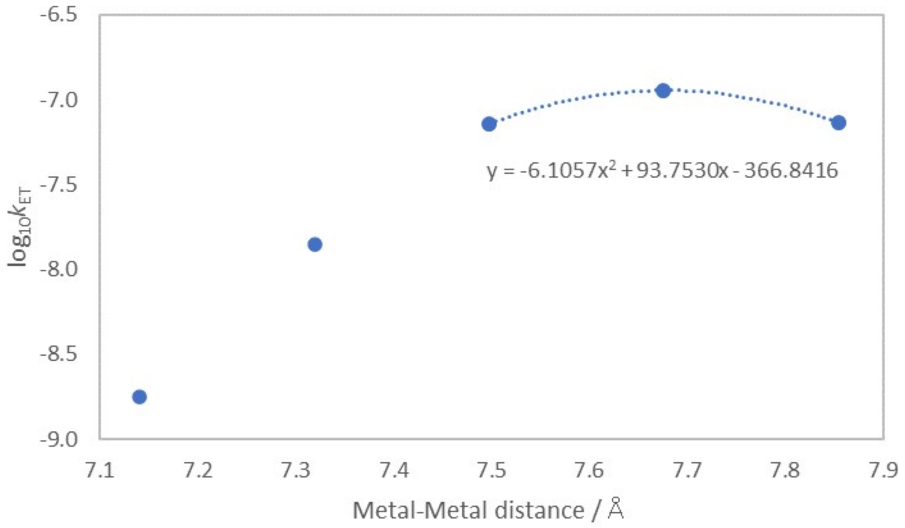

Fig. S6 Plots of $\log_{10}k_{ET}$ maximization for $[Co(NH_3)_6]^{3+/2+}$ (Cycle 1).

$[Ru(NH_3)_6]^{3+/2+}$

Cycle 1 (Converged)

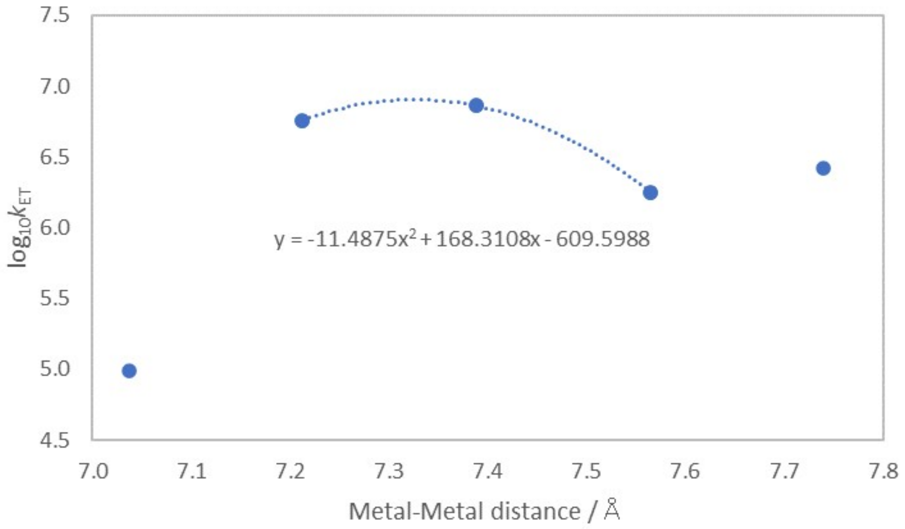

Fig. S7 Plots of $\log_{10}k_{ET}$ maximization for $[Ru(NH_3)_6]^{3+/2+}$ (Cycle 1).

$[Ru(NH_3)_5py]^{3+/2+}$

Cycle 1 (Converged)

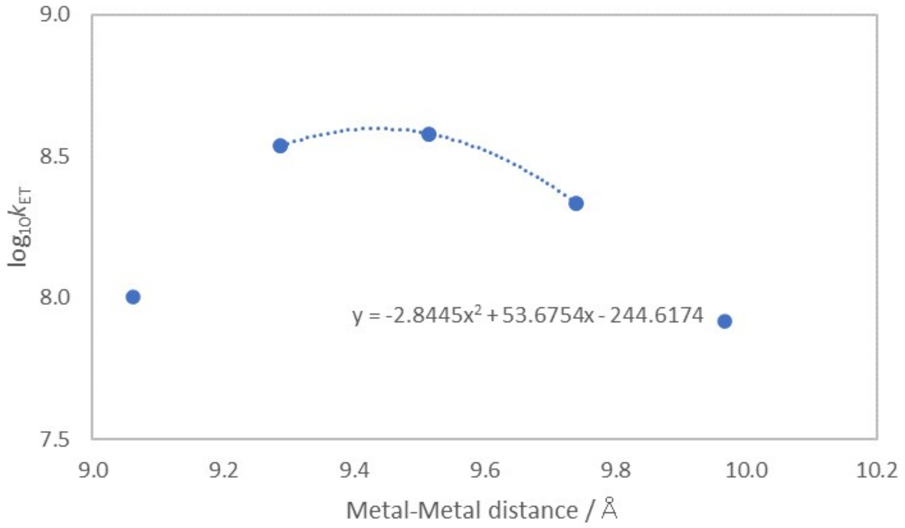

Fig. S8 Plots of $\log_{10}k_{ET}$ maximization for $[Ru(NH_3)_5py]^{3+/2+}$ (Cycle 1).

$[Co(en)_3]^{3+/2+}$

Cycle 1 (Converged)

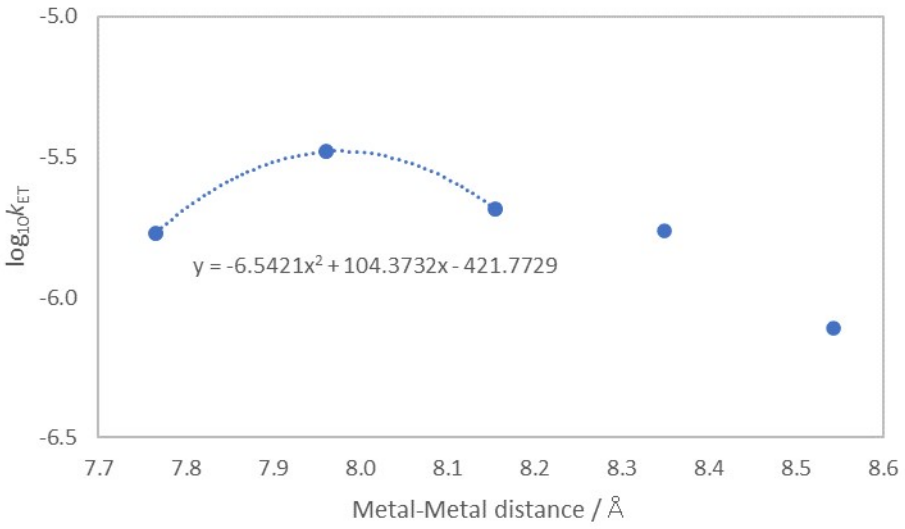

Fig. S9 Plots of $\log_{10}k_{ET}$ maximization for $[Co(en)_3]^{3+/2+}$ (Cycle 1).

$[Ru(en)_3]^{3+/2+}$

Cycle 1 (Converged)

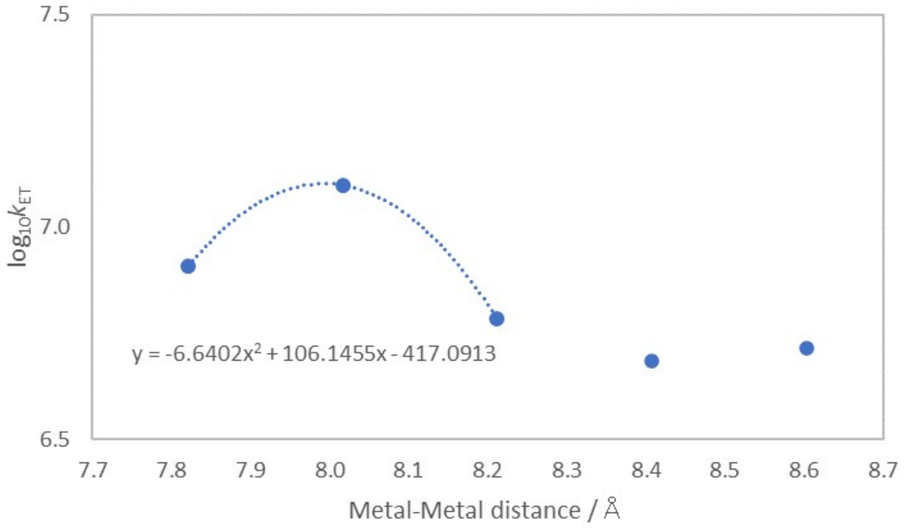

Fig. S10 Plots of $\log_{10}k_{ET}$ maximization for $[Ru(en)_3]^{3+/2+}$ (Cycle 1).

$[Fe(bpy)_3]^{3+/2+}$

Cycle 1 (Converged)

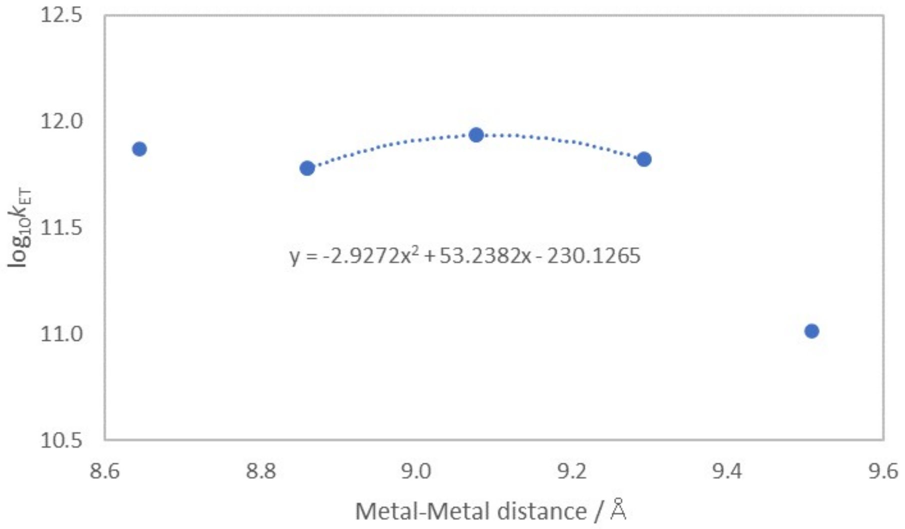

Fig. S11 Plots of $\log_{10}k_{ET}$ maximization for $[Fe(bpy)_3]^{3+/2+}$ (Cycle 1).

$[Co(bpy)_3]^{3+/2+}$

Cycle 1 (Converged)

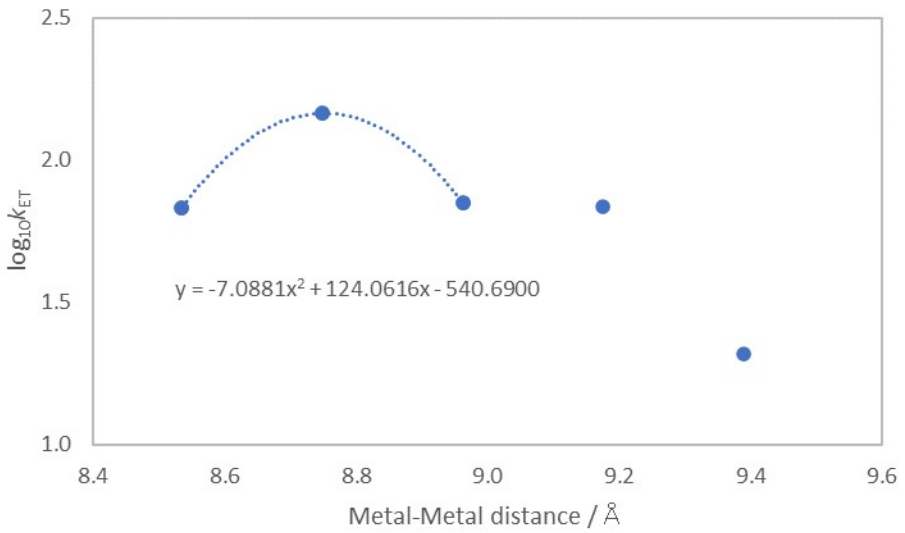

Fig. S12 Plots of $\log_{10}k_{ET}$ maximization for $[Co(bpy)_3]^{3+/2+}$ (Cycle 1).

$[Ru(bpy)_3]^{3+/2+}$

Cycle 1 (Converged)

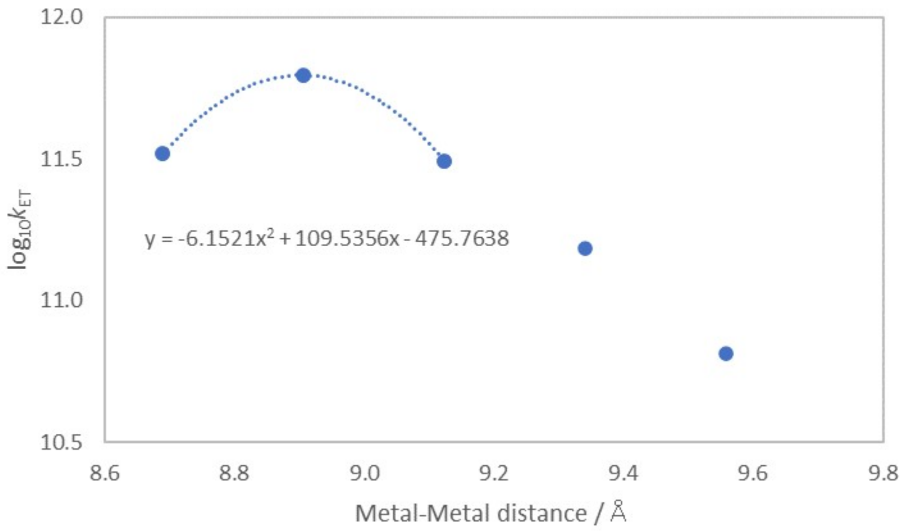

Fig. S13 Plots of $\log_{10}k_{ET}$ maximization for $[Ru(bpy)_3]^{3+/2+}$ (Cycle 1).

3. Cartesian coordinates and energies of the most stable monomer equilibrium geometries in Gibbs energy with charge 3+ and 2+ at the UωB97X-D/Def2-TZVP//Def2-SV(P) level

Extrapolated Gibbs energies of the most stable monomers in Gibbs energy were used for calculation of Gibbs energy barrier $\Delta G^{\ddagger}$. In the following, energy values are given in Hartree.

$[V(H_2O)_6]^{3+}$

E(Def2-SV(P)) = -1401.55157805

G(Def2-SV(P)) = -1401.43905650

E(Def2-TZVP) = -1402.32990322

3 3

V	-0.000000718176	-0.000000924618	-0.000002228106
O	1.580932683322	-0.003265161189	-1.238774996193
O	0.823172303842	1.558243218908	0.959936758312
O	-1.580936453772	0.003292442933	1.238764805553
O	-0.823161037544	-1.558243003639	-0.959948567999
O	-1.006794620668	1.296326192159	-1.155435761477
O	1.006797948900	-1.296332900441	1.155428504369
H	1.970960155507	-1.442452512138	1.108726060928
H	1.820480127231	-0.729429052910	-1.845611239358
H	1.618398399720	1.519310937544	1.525231181247
H	-2.173055327015	-0.757349907817	1.394005334417
H	-0.471146478525	-2.468954263157	-0.956448028659
H	-1.970959569380	1.442430586524	-1.108745624546
H	2.173045052159	0.757357354406	-1.393987046076
H	0.471165163167	2.468959587524	0.956438687096
H	-1.820429336593	0.729450987559	1.845627699535
H	-1.618436089872	-1.519346388539	-1.525178693933
H	-0.623457836223	1.871655571113	-1.844541140428
H	0.623462346713	-1.871660103893	1.844537409332

$[Cr(H_2O)_6]^{3+}$

E(Def2-SV(P)) = -1501.98179229

G(Def2-SV(P)) = -1501.87136056

E(Def2-TZVP) = -1502.78864689

3 4

Cr	-0.000018496649	-0.000008380572	-0.000002867889

O	0.826454683020	-1.427046760033	-1.076690369239
O	-0.850473090002	-1.402861617219	1.092451404480
O	-0.826516912746	1.427066177070	1.076631401998
O	0.850430778486	1.402840271373	-1.092466815249
O	1.590563261952	-0.016237296661	1.162403150993
O	-1.590598958243	0.016201190015	-1.162407274280
H	-1.668798707455	-0.470206304851	-2.005163118880
H	1.330321103385	-1.283071103893	-1.900357801268
H	-1.788128803631	-1.657050766847	0.993008389282
H	-0.573466865902	2.366209465879	0.990105080040
H	0.499723496103	1.725774225134	-1.944422085634
H	1.668750934643	0.470068736151	2.005203466852
H	0.573582162010	-2.366234743513	-0.990201787849
H	-0.499834935140	-1.725710934601	1.944452232147
H	-1.330011350326	1.283153113674	1.900531017878
H	1.788040224219	1.657174180060	-0.992984107676
H	2.291971898444	-0.691718108451	1.090286473673
H	-2.292011251046	0.691687471120	-1.090363736402

$[Fe(H_2O)_6]^{3+}$

E(Def2-SV(P)) = -1721.16334277

G(Def2-SV(P)) = -1721.05654896

E(Def2-TZVP) = -1721.98416054

3 6

Fe	-0.000068055920	0.000053986346	0.000089367004
O	1.037879442145	-1.571330561020	0.702479001137
O	1.095446233417	1.237697745136	1.143602186576
O	-1.038010884309	1.571414473525	-0.702328259659
O	-1.095346934310	-1.237622926447	-1.143637670919
O	1.329474124631	0.206025467883	-1.492595756776
O	-1.329482009891	-0.205957066771	1.492825812200
H	-1.290269324999	-0.886593729806	2.191883147212
H	0.891687586125	-2.502004376093	0.445219664914
H	0.872063834691	1.508202398590	2.054918566926
H	-1.758396621985	1.519496581180	-1.359615919394
H	-1.930714035839	-1.662414368158	-0.868243312807

H	1.289930565037	0.886137617829	-2.192147708547
H	1.758410984908	-1.519408573354	1.359610526042
H	1.930734089755	1.662444499769	0.867883950300
H	-0.891599643311	2.502125593647	-0.445324541967
H	-0.871594925947	-1.508154954679	-2.054848504285
H	2.105830514724	-0.369145771795	-1.634204902679
H	-2.105974871227	0.369034005953	1.634434340045

$[Co(H_2O)_6]^{3+}$

E(Def2-SV(P)) = -1840.15558935

G(Def2-SV(P)) = -1840.04677124

E(Def2-TZVP) = -1840.99306444

3 5

Co	-0.000014427622	0.000151980670	0.000035349072
O	-0.427381389260	1.092451960986	-1.598083318806
O	-1.370298639218	0.956390847425	1.062692356888
O	0.427661310194	-1.091899483087	1.598325786032
O	1.370443285020	-0.956362749854	-1.062214132805
O	-1.344223851932	-1.353742679612	-0.544124318189
O	1.344206336704	1.354047418861	0.544004667362
H	1.316094109174	2.297543565645	0.293160912243
H	0.191042396882	1.246087248471	-2.338332788544
H	-1.174925249119	1.561742674818	1.803963012032
H	1.204889868735	-1.677119733253	1.685624710285
H	2.334840233943	-0.818478683556	-0.990666333147
H	-1.315719309596	-2.297400679398	-0.293939682056
H	-1.205751735381	1.675941400908	-1.686658945797
H	-2.334691990609	0.818391557940	0.991455869401
H	-0.191043562980	-1.246119699074	2.338240053321
H	1.175292170023	-1.561703088399	-1.803543346986
H	-2.005811386481	-1.218650972822	-1.249931639925
H	2.005823608034	1.219028628065	1.249771988482

$[Ru(H_2O)_6]^{3+}$

E(Def2-SV(P)) = -552.45329177

G(Def2-SV(P)) = -552.33839794

E(Def2-TZVP) = -553.13265534

3 2

Ru	0.000013134768	0.000005234743	-0.000015873791
O	1.426063679585	0.259350180016	-1.438713668790
O	1.519745017563	-0.858955125152	1.110005558188
O	-1.426109173204	-0.259464547390	1.438615237767
O	-1.519702992298	0.858925369555	-1.110064425814
O	0.483184080945	1.862106429058	0.695714398504
O	-0.483141230924	-1.862103882745	-0.695689844504
H	0.095625137107	-2.372484288491	-1.294723395177
H	1.251668768833	0.173308666183	-2.395401297368
H	1.625388350654	-1.829248684304	1.075803721069
H	-2.358978653816	-0.042243269586	1.246582594509
H	-1.692205354429	0.564829371688	-2.024803951875
H	-0.095639224249	2.372479737612	1.294704544017
H	2.359020670862	0.042553257507	-1.246694351698
H	1.692359361919	-0.564746510861	2.024685976699
H	-1.251719378119	-0.173280098085	2.395298387735
H	-1.625606100687	1.829180505115	-1.075689612325
H	1.407098903302	2.159616628023	0.803356058745
H	-1.407058455704	-2.159709216674	-0.803092696279

$[Co(NH_3)_6]^{3+}$

E(Def2-SV(P)) = -1721.27123844

G(Def2-SV(P)) = -1721.06074309

E(Def2-TZVP) = -1721.92945932

3 1

Co	-0.039039321148	-0.031934969229	-0.013944293531
N	1.349662908716	0.695036838358	1.204121032490
H	2.203686050075	0.127770818683	1.244994073766
H	1.038616549932	0.771020047574	2.178835155095
H	1.666903686429	1.636705816175	0.948427115981
N	-0.208063119398	-1.576576967395	1.222223810181
H	-0.625720951147	-2.414672031937	0.803476707447
H	-0.780876838062	-1.379520663446	2.050611624620
H	0.698237482102	-1.884254665936	1.591699434575

N	-1.438567976307	-0.768206591704	-1.216411104785
H	-2.331233179429	-0.265753350448	-1.156403408706
H	-1.674130400960	-1.747349207272	-1.022667770234
H	-1.185966350442	-0.750888231351	-2.210361096101
N	-1.453717273509	0.946833516847	0.976597137645
H	-2.225060351472	0.342736016192	1.283244701022
H	-1.897836960754	1.689449164987	0.425289431026
H	-1.120749663851	1.413996086947	1.827638850885
N	0.195106319463	1.536090975435	-1.208007679133
H	-0.517016240962	1.613393714541	-1.942877096909
H	1.094006666374	1.538917488758	-1.703963250713
H	0.161113237842	2.430579029618	-0.706083603039
N	1.333867929646	-1.018203328590	-1.051454522600
H	1.373739934433	-2.017054115358	-0.819958536655
H	2.290157032980	-0.670991706755	-0.917805241973
H	1.177660693040	-0.986642940789	-2.065547105792

$[Ru(NH_3)_6]^{3+}$

E(Def2-SV(P)) = -433.55132867

G(Def2-SV(P)) = -433.34621320

E(Def2-TZVP) = -434.05161202

3 2

Ru	-0.010921595185	-0.031620917362	-0.034512243692
N	1.618631182376	-0.121950413188	1.346117063025
H	1.338176033695	0.098939460749	2.307707845236
H	2.374192152428	0.537589773333	1.129636964828
H	2.058334787036	-1.047669641337	1.391684109117
N	-0.964315454513	-1.591259866532	1.071630106387
H	-0.629934954894	-1.672222531277	2.037998900355
H	-0.842596194602	-2.519740447965	0.653855166650
H	-1.980597425318	-1.464901407753	1.142964564898
N	-1.667984463080	0.005495588196	-1.382204013728
H	-1.484502395464	0.521179927471	-2.249466935010
H	-2.500036832942	0.451453011819	-0.979096928545
H	-1.969897382721	-0.931714429291	-1.670323108647
N	-0.909674343178	1.464289498587	1.201242766658

H	-1.574666904999	1.086601664808	1.885591328571
H	-1.428313702243	2.173471567248	0.671553470890
H	-0.217732011844	1.981801595337	1.754125163393
N	0.916151910352	1.545677800543	-1.135241240642
H	0.872368637188	2.449599950581	-0.652973697277
H	0.496245575728	1.698107795386	-2.057707172902
H	1.913020726507	1.385862954916	-1.321609227331
N	0.946147760970	-1.469041267477	-1.288267339783
H	0.823015336568	-2.439045418019	-0.979439672286
H	1.962920709014	-1.331912401832	-1.336193207818
H	0.622114362905	-1.434783098150	-2.260852783522

$[Ru(NH_3)_5py]^{3+}$

E(Def2-SV(P)) = -625.07443096

G(Def2-SV(P)) = -624.82057688

E(Def2-TZVP) = -625.77056772

3 2

Ru	-0.891071808380	-0.006525875679	0.013783501321
N	-0.925866633013	1.586381026401	-1.411890418781
H	-1.681103328309	1.499697742605	-2.100794045070
H	-0.064390019131	1.630061879199	-1.968277818687
H	-1.052446869164	2.517449407308	-1.001737834337
N	-0.905341586779	1.416630194905	1.609730295818
H	-1.593795285519	1.200484000804	2.339012034946
H	-1.115023439356	2.377770891509	1.320648244296
H	-0.005872767946	1.458371021425	2.102972560630
N	-0.891927569729	-1.590768033796	1.449674971095
H	-1.055660270572	-2.523792349005	1.056765141006
H	-1.605996935277	-1.476703643823	2.177772755941
H	-0.003544720561	-1.648140157758	1.961077527103
N	-3.029260941241	-0.029542057426	0.033329568650
H	-3.431466484158	0.007087561413	-0.910081045245
H	-3.444518162235	0.764686295228	0.532834031835
H	-3.419823364501	-0.879329070392	0.455510462364
N	-0.929237313022	-1.426955348378	-1.583831193160
H	-0.031166350158	-1.484626883086	-2.077635235924

H	-1.612877789138	-1.194139128958	-2.312794902431
H	-1.162927815822	-2.382939547108	-1.296510124451
C	3.998698024258	-0.010740243429	-0.016215586504
C	3.292611575789	1.189897648029	0.019924403192
C	1.907844606770	1.153009623833	0.023316046718
N	1.214654526808	-0.003363044049	-0.006827291634
C	1.901887366205	-1.163362597191	-0.043069766281
C	3.286250085868	-1.207777497722	-0.049060865795
H	5.091204007360	-0.013658526350	-0.018363481825
H	3.801906015550	2.154349977489	0.046372259483
H	1.337122569474	2.082593320336	0.051358406726
H	1.326050161402	-2.089992124399	-0.066733603886
H	3.790150199901	-2.174996063527	-0.078137274600

$[Co(en)_3]^{3+}$

E(Def2-SV(P)) = -1953.27969345

G(Def2-SV(P)) = -1952.95604309

E(Def2-TZVP) = -1954.16123756

3 1

Co	0.004768655305	0.001303462327	0.007297407188
N	1.229685065693	1.138681629088	1.089042267389
N	-0.271379885743	1.647938624257	-1.073865932785
N	1.506961967658	-0.615237791826	-1.138691411562
N	0.431777725557	-1.586141572249	1.120158257372
N	-1.570408504847	0.429968118187	1.144729557429
N	-1.296763474647	-1.009259247419	-1.102678997030
C	1.524451213453	2.394760684963	0.351430126248
C	0.249399374469	2.825666756078	-0.333233360817
C	1.844446398621	-2.022663899945	-0.798580644038
C	1.737221638982	-2.163783557835	0.702636402661
C	-2.713687844621	-0.442785268843	0.768468599345
C	-2.681897797860	-0.621093922997	-0.731836854455
H	1.309766465909	-0.549284785215	-2.141949532882
H	2.339355835309	-0.032524304210	-1.008467595561
H	-0.294615866851	-2.305876060385	1.048878592096
H	0.464240655409	-1.368275976164	2.120863889122

H	0.228763324778	1.576028322488	-1.965207507814
H	-1.244636782207	1.817738201569	-1.346383738940
H	2.109832028734	0.681822348765	1.346649516507
H	0.803214536466	1.375428203361	1.990914573056
H	-1.847370475199	1.410371409845	1.036045248497
H	-1.394523901480	0.330845481265	2.149129462690
H	-1.190274351534	-2.020241442762	-0.972146823372
H	-1.161823412836	-0.871411204809	-2.109517638888
H	-3.400577954454	-1.384193375713	-1.063964430473
H	-2.931576324747	0.314871317740	-1.252767605741
H	-3.663275064836	-0.002500913830	1.105264843171
H	-2.593348419483	-1.407764393855	1.283259971297
H	2.313741865574	2.190325633206	-0.387370360129
H	1.902463936060	3.168258156173	1.035319160337
H	-0.505009988410	3.143406209818	0.402123778901
H	0.419969195400	3.668837486106	-1.017959940930
H	2.540136213946	-1.606054917683	1.207753085824
H	1.815325513099	-3.213774109996	1.019245876981
H	1.135650050359	-2.684549332437	-1.318757581999
H	2.853451348068	-2.275337250000	-1.155460817187

$[Ru(en)_3]^{3+}$

E(Def2-SV(P)) = -665.55523862

G(Def2-SV(P)) = -665.23579066

E(Def2-TZVP) = -666.27945288

3 2

Ru	-0.002477139515	-0.006755221349	-0.012409173744
N	-1.097905674956	-1.478470111362	-1.111508378302
N	-1.820889215130	-0.000290355077	1.120336668463
N	0.926730056829	-1.559077978025	1.126297090549
N	1.799092542236	-0.235306581907	-1.145646931912
N	-0.740925613440	1.682288117192	-1.109841462699
N	0.930127826740	1.563296548094	1.097653946941
C	-2.537135488786	-1.400428451697	-0.742895953628
C	-2.643240333351	-1.184555119424	0.751034497641
C	2.367759023824	-1.639632474963	0.765010482633

C	2.494752532402	-1.485386915305	-0.734604895928
C	0.033748902244	2.895968493644	-0.734430632612
C	0.292742243999	2.863740062839	0.755971246724
H	0.841578659398	-1.399873780967	2.136004902377
H	0.487938130537	-2.474196144663	0.983261658778
H	2.433372972210	0.561093955910	-1.032275530623
H	1.620570482527	-0.266683035073	-2.155016391240
H	-1.650458900892	-0.012341787486	2.131636125033
H	-2.368946549033	0.852364155538	0.973029973813
H	-0.751657068101	-2.428340554501	-0.946906861464
H	-1.004122752056	-1.354259806428	-2.125310972276
H	-1.737064606396	1.850779915905	-0.941913610223
H	-0.683044489442	1.548508583748	-2.125094081941
H	1.937521744117	1.621197214811	0.919326294172
H	0.866026651433	1.412878694204	2.110089140310
H	0.937630023130	3.699905124558	1.063821520102
H	-0.648455506644	2.940515087716	1.321110377002
H	-0.515185815933	3.805441587503	-1.020021394702
H	0.978482672999	2.883694262838	-1.297932022994
H	-3.063387037596	-2.316724588037	-1.048477720570
H	-2.981961076180	-0.557170133648	-1.292929769325
H	-3.689690599528	-1.033294345869	1.054032996466
H	-2.257128798304	-2.053893112273	1.303545480344
H	2.016545174075	-2.328421973883	-1.254924086275
H	3.550254094186	-1.455940587107	-1.042581843206
H	2.896437377548	-0.830908426695	1.291891392468
H	2.795520277892	-2.595126774293	1.102618769760

$[Fe(bpy)_3]^{3+}$

E(Def2-SV(P)) = -2747.82188920

G(Def2-SV(P)) = -2747.38176418

E(Def2-TZVP) = -2749.60294216

3 2

Fe	0.006046074133	0.005225288415	0.004324106429
C	4.118603387767	0.728187356143	-2.240210063942
C	0.122395598837	2.156615710714	2.039103690106

C	0.677535968143	3.262887797649	2.668164716523
C	1.897846296191	3.749318787749	2.211054051758
C	2.526374349837	3.115075386848	1.142164468533
C	1.916586499177	2.010147423831	0.559711386464
C	2.473775362163	1.251955676028	-0.575491225402
C	3.701301367736	1.517495689613	-1.171233135964
C	3.300079553265	-0.305833897409	-2.682710688500
C	2.087305477147	-0.518220398997	-2.040974367551
H	-0.829831192301	1.743835969995	2.374003171683
H	0.150595492397	3.727443257841	3.502635963286
H	3.480816181389	3.485535765404	0.768954367125
H	4.331744122788	2.329637367652	-0.809745505946
H	3.587010421653	-0.948501911199	-3.516048786047
H	1.417070328832	-1.315336964388	-2.364396490337
N	0.730734313922	1.547008010627	1.014701062836
N	1.685972970159	0.246417607110	-1.018339916702
C	-1.498087909556	-1.542423935100	-2.027531439954
C	-1.938941241920	-2.701922819773	-2.651229362022
C	-1.470389806966	-3.928110722419	-2.191044401899
C	-0.573961351632	-3.958363475357	-1.125535903771
C	-0.169482531152	-2.759569150594	-0.549661268963
C	0.778717430375	-2.651738583250	0.574464106579
C	1.431653018717	-3.730709516453	1.159475864891
C	2.312684636473	-3.498207167329	2.212893879318
C	2.518489094685	-2.194572605073	2.652305463036
C	1.835284447819	-1.163040907967	2.022530583430
C	-0.601168578560	2.074092234313	-2.030345817316
C	-1.402769724302	3.007003417717	-2.674393321414
C	-2.711642044463	3.179584648452	-2.236275585822
C	-3.178180560993	2.417171988447	-1.168114522436
C	-2.322849799751	1.499669678618	-0.569123933927
C	-2.690715495758	0.635958702594	0.567857055526
C	-3.952190032217	0.598334583075	1.150657231373
C	-4.175775125389	-0.259410577986	2.224872487360
C	-3.133690878128	-1.056509473441	2.686848810623
C	-1.899800050080	-0.972470228895	2.056113063696

H	-1.838640832564	-0.563440496180	-2.366326161285
H	-2.639505297265	-2.630704788619	-3.484285381051
H	-0.198784039597	-4.910730017183	-0.751718123553
H	1.262752062353	-4.745664544450	0.800669595208
H	3.198554517727	-1.965710906573	3.473875940483
H	1.966108578960	-0.129479724302	2.344469666617
H	0.427166290022	1.907005055639	-2.351929005977
H	-0.995123392832	3.582422025223	-3.506582130549
H	-4.199453610382	2.542909812448	-0.809540203573
H	-4.758659309047	1.227248512588	0.774640681390
H	-3.262429221462	-1.741424088724	3.525962702740
H	-1.058389028970	-1.578334338765	2.394656996671
N	-0.634053824725	-1.574815463307	-1.005910775127
N	0.985852109534	-1.390138536306	1.013534593463
N	-1.054533313263	1.337907461651	-1.008656608743
N	-1.686731496288	-0.144663129463	1.026175225942
H	5.079374781366	0.923197019884	-2.721133979920
H	2.362659062940	4.619067768240	2.680000567737
H	-5.160716498062	-0.301391989721	2.694498269033
H	-3.370738152641	3.904018860382	-2.719198313607
H	-1.797781194194	-4.860427129141	-2.655844604117
H	2.834069733361	-4.334255271282	2.683617514493

$[Co(bpy)_3]^{3+}$

E(Def2-SV(P)) = -2866.88356163

G(Def2-SV(P)) = -2866.44192421

E(Def2-TZVP) = -2868.68092148

3 1

Co	-0.001497542019	-0.005442556684	-0.003108999360
C	0.102372875412	4.156623478490	2.195860834509
C	2.060806013210	0.378232688369	-2.067913975925
C	3.066364528864	1.084735379328	-2.714701155786
C	3.373866931487	2.366774145552	-2.272813471551
C	2.667250384999	2.903241166519	-1.199070197460
C	1.672129366650	2.141606438287	-0.597803563042
C	0.849607957543	2.591476792447	0.539388219968

C	0.934352528322	3.850519489596	1.121615717620
C	-0.790809070875	3.195991675805	2.657522127935
C	-0.822992569638	1.956960864324	2.030808502556
H	1.790930446758	-0.626223391794	-2.393806226444
H	3.591382306578	0.623502207077	-3.552194782178
H	2.895701324599	3.905499046358	-0.837181749303
H	1.638509116731	4.591359848239	0.743850668344
H	-1.460627770030	3.390079938259	3.496243206662
H	-1.503070896432	1.177552995683	2.374170703074
N	1.382524681719	0.897587271560	-1.039116993769
N	-0.020026741645	1.666024809449	1.001495248013
C	-1.277838081225	-1.659134914275	2.071233649408
C	-2.363290519512	-2.235079320652	2.718512262057
C	-3.645107107999	-1.940914613735	2.266875240967
C	-3.802848872336	-1.081727488305	1.182075952043
C	-2.673361822261	-0.540636806936	0.579448685785
C	-2.701569949571	0.376411822392	-0.574102555392
C	-3.861871670076	0.862219334331	-1.165345400267
C	-3.756574174039	1.717956224444	-2.259219521050
C	-2.495230836540	2.061958679132	-2.732652886603
C	-1.376853529011	1.542061888031	-2.094136581089
C	2.080189189808	-0.231859298647	2.066379473596
C	3.136434548199	-0.860368183067	2.713021466298
C	3.539675449361	-2.115963133073	2.271972111082
C	2.875840500445	-2.705090724031	1.198563507948
C	1.826685295841	-2.020805263583	0.596259385035
C	1.040155224118	-2.532424701130	-0.540674744325
C	1.215805777229	-3.783832156170	-1.119007090365
C	0.406802965238	-4.153562423221	-2.190876409445
C	-0.554602529037	-3.262190907399	-2.654285109715
C	-0.676628940590	-2.027039454773	-2.031099410280
H	-0.260334356318	-1.862042649118	2.404937783672
H	-2.190920679653	-2.901632239541	3.564539806552
H	-4.799621568495	-0.841710894292	0.812933707751
H	-4.842188908653	0.581899289255	-0.780780380734
H	-2.363759504003	2.726053643063	-3.587894348220

H	-0.373976677953	1.786884011170	-2.443256550897
H	1.733442640116	0.748836390735	2.392306428502
H	3.625555968084	-0.360984773091	3.550358384997
H	3.179670045243	-3.687413148315	0.837251057205
H	1.972149650649	-4.470359199733	-0.739467825898
H	-1.210100585003	-3.507741524665	-3.490830398987
H	-1.414449329034	-1.302013516200	-2.374373330781
N	-1.433532584744	-0.832251542062	1.031555694355
N	-1.482091810797	0.721502179485	-1.043325277439
N	1.443271142420	-0.802286641900	1.037688173921
N	0.104667554788	-1.674444334821	-1.004526818183
H	0.155393443364	5.141164095899	2.665118855333
H	4.159118600631	2.950544721314	-2.757424186924
H	0.530545239961	-5.133316020430	-2.656768821903
H	4.366533619789	-2.638855320972	2.756961230926
H	-4.521577303297	-2.375468262676	2.751920948891
H	-4.657559592531	2.110504204204	-2.735076482419

$[Ru(bpy)_3]^{3+}$

E(Def2-SV(P)) = -1579.15775736

G(Def2-SV(P)) = -1578.71940928

E(Def2-TZVP) = -1580.80258700

3 2

Ru	-0.003716769593	-0.003592363058	0.000701362438
C	-1.831263851366	3.923459494443	2.177307492818
C	1.765549372639	1.456478370704	-2.007835259347
C	2.351475673087	2.552351094100	-2.625340661292
C	2.015107642857	3.826629146326	-2.179646755201
C	1.105771728294	3.966616130958	-1.134387780621
C	0.551739207200	2.828293795491	-0.557822211892
C	-0.417398773829	2.853221477070	0.557463586940
C	-0.911432030262	4.017977032883	1.136097104409
C	-2.239331526799	2.667780215771	2.615971152216
C	-1.710450217406	1.544052886442	1.997075278707
H	1.995540375257	0.438657967948	-2.327275350176
H	3.058678900130	2.397045082815	-3.441085024526

H	0.836684931344	4.960021937638	-0.776865826506
H	-0.587953274572	4.996770692169	0.783189148024
H	-2.958345223856	2.548166394219	3.427381945762
H	-1.996280758863	0.538749187361	2.311083522370
N	0.887520244054	1.594716018680	-1.005517841905
N	-0.820673264553	1.638462757343	1.000189555978
C	-0.491689671712	-2.234057404352	2.018346501955
C	-1.197549832886	-3.253900052822	2.640750146343
C	-2.481902809038	-3.545343743382	2.192004402431
C	-3.021331223607	-2.809756563010	1.139741616183
C	-2.264056609714	-1.796968961927	0.560349796780
C	-2.728365668555	-0.953281758481	-0.560072339474
C	-3.988793707453	-1.052124718775	-1.140202829354
C	-4.323719431027	-0.200261238212	-2.189641653385
C	-3.392973657663	0.733545357388	-2.634161089315
C	-2.153771111343	0.784535111432	-2.011586526094
C	2.151927365522	0.667156113729	2.046205917841
C	3.382965884224	0.560672795339	2.678026157167
C	4.286973852735	-0.392292385793	2.219170370765
C	3.933420501130	-1.209676447999	1.148504956468
C	2.681925421800	-1.056812666403	0.561189937613
C	2.195059657804	-1.866845419959	-0.574266605624
C	2.924432107416	-2.888598170399	-1.173308167899
C	2.361349894970	-3.594920570437	-2.233335604808
C	1.081002915110	-3.267490416469	-2.668493312806
C	0.404388947404	-2.239122092585	-2.027858228583
H	0.516478762500	-1.967290794635	2.339810321812
H	-0.737134733437	-3.803913835971	3.462480031618
H	-4.025185100128	-3.030870991479	0.778121575314
H	-4.711143233321	-1.784372628695	-0.780756175307
H	-3.613727181880	1.419995871587	-3.452583211449
H	-1.390867549036	1.497811569844	-2.328159298373
H	1.409559677994	1.397217129923	2.373043334930
H	3.618501540955	1.219790093110	3.514578995334
H	4.633471176671	-1.958671706161	0.779525168972
H	3.924726660119	-3.139161769856	-0.820885591940

H	0.601942784875	-3.795225820465	-3.494152717107
H	-0.599140382583	-1.943363391070	-2.338690398915
N	-1.015060580351	-1.525712757170	1.008867199880
N	-1.832819380852	-0.040988609982	-1.006644892067
N	1.813867623697	-0.123866005007	1.019474735849
N	0.951851104273	-1.556574704670	-1.013501771058
H	-2.226299113258	4.830560648894	2.639455217057
H	2.456889681727	4.712873645044	-2.639925397049
H	2.923935976951	-4.398542478046	-2.713055379674
H	5.266152129474	-0.503495680608	2.689596877447
H	-3.066286052593	-4.342776123264	2.655690811637
H	-5.309856251773	-0.268589209222	-2.653697040393

$[V(H_2O)_6]^{2+}$

E(Def2-SV(P)) = -1401.76024677

G(Def2-SV(P)) = -1401.65649109

E(Def2-TZVP) = -1402.53907882

2 4

V	-0.009483051014	-0.017714094034	0.010551185517
O	-0.008831344580	-2.116568462797	-0.397030411124
O	1.527017234098	-0.208094709746	1.480108982820
O	0.080512140750	2.090213573514	0.318732874646
O	-1.542101739423	0.311111412510	-1.437050492490
O	1.555654481253	0.190119934985	-1.422971257061
O	-1.590354386945	-0.229000064553	1.434418023245
H	-1.778897093440	-1.084026320169	1.853898205845
H	-0.767453010069	-2.559179486088	-0.810446171429
H	1.404826285763	-0.664371544324	2.327880037303
H	-0.653445548836	2.674150917515	0.069272402778
H	-2.460470847710	0.087671735599	-1.215193144965
H	1.730015254768	1.052354689050	-1.833522558170
H	0.412097119384	-2.757780844554	0.197883170877
H	2.435309779103	-0.379546851184	1.182646324103
H	0.492966373694	2.455889993152	1.117643617637
H	-1.423715549826	0.161272265489	-2.388604551169
H	1.700754891849	-0.492611091638	-2.097694150736

H	-1.773344572545	0.461018988090	2.092520562944

$[Cr(H_2O)_6]^{2+}$

E(Def2-SV(P)) = -1502.18487173

G(Def2-SV(P)) = -1502.08009592

E(Def2-TZVP) = -1502.98439553

2 5

Cr	-0.007533862192	-0.020045596470	-0.039202418370
O	-1.009396779682	0.896499610139	1.533120693487
O	0.809889346167	1.761310592131	-0.770454260945
O	0.994276909139	-0.936487931612	-1.611729478723
O	-0.825136927131	-1.801275953999	0.692076434418
O	2.189265466547	0.400421227316	0.969119129599
O	-2.204142477610	-0.442646712027	-1.049064962893
H	-2.900800780268	0.181917892867	-0.790132102762
H	-1.300260109008	0.322001450647	2.261455814521
H	0.332083254034	2.597424929551	-0.892796801752
H	0.730250148342	-1.791699712440	-1.989260461583
H	-1.625006897296	-1.956091101900	0.150509800321
H	2.885042615077	-0.224717491323	0.709108689080
H	-0.744595304881	1.751172487285	1.911331689365
H	1.610410035781	1.915627336881	-0.229758050361
H	1.284239117736	-0.362183800067	-2.340564417643
H	-0.347148457542	-2.637125489102	0.815434587338
H	2.232996412213	0.469624415574	1.936437287735
H	-2.246818484127	-0.512415944296	-2.016373775861

$[Fe(H_2O)_6]^{2+}$

E(Def2-SV(P)) = -1721.41250616

G(Def2-SV(P)) = -1721.31131787

E(Def2-TZVP) = -1722.23747852

2 5

Fe	0.001712187692	0.030841357059	0.015303509437
O	-1.645798949710	0.730608793716	-1.128565786625
O	-0.202740826049	-1.908835347583	-0.834718238099
O	1.649142252792	-0.669191650701	1.159207717217

O	0.206400235407	1.970577752793	0.865069494394
O	1.358182503882	0.542514360351	-1.526066674228
O	-1.354578285816	-0.480945799214	1.556795980755
H	-2.311603380868	-0.389219294455	1.420532309927
H	-2.012159005498	1.617237725674	-0.980023284514
H	-1.062373642365	-2.348808708930	-0.936315999571
H	1.791476670659	-0.447200726608	2.093281104270
H	-0.317387175986	2.172491500414	1.657704452348
H	2.315177497339	0.450634603319	-1.389659737183
H	-1.787902136201	0.508934836566	-2.062772941567
H	0.321177764288	-2.110456370701	-1.627334725302
H	2.014806319445	-1.556185819996	1.011082039566
H	1.066034317834	2.410668177941	0.966332835532
H	1.217724273378	1.284273749612	-2.136468361432
H	-1.213903692694	-1.222684521737	2.167188653376

$[Co(H_2O)_6]^{2+}$

E(Def2-SV(P)) = -1840.45625965

G(Def2-SV(P)) = -1840.35244690

E(Def2-TZVP) = -1841.29835455

2 4

Co	0.004614989805	0.046279633442	-0.036584331338
O	-1.331871477961	0.067570726730	-1.635443341706
O	0.977083976980	-1.457683795656	-1.134038062028
O	1.427092334216	0.063417526508	1.486506416710
O	-1.042802778749	1.560636844663	0.932358489073
O	1.294171326617	1.324532960995	-1.082473716509
O	-1.261500091717	-1.277906485375	0.985961019808
H	-1.762473133171	-1.959094214857	0.508459288574
H	-1.188012856387	-0.593729294477	-2.332277923753
H	0.973441470331	-2.393237611826	-0.874948803577
H	1.231106608378	0.224419195606	2.423264239158
H	-1.777119155697	1.302209902747	1.512950943643
H	0.967385731562	1.786064749120	-1.872104158898
H	-2.288837816293	0.195780899168	-1.535367980693
H	1.856481072636	-1.250984183765	-1.490640478966

H	2.266601620857	-0.421041332506	1.434902084604
H	-0.612577104176	2.337174845636	1.324834505272
H	1.852821182236	1.946273455076	-0.588559901778
H	-1.006050516955	-1.653832565494	1.844197500056

$[Ru(H_2O)_6]^{2+}$

E(Def2-SV(P)) = -552.68239320

G(Def2-SV(P)) = -552.56978285

E(Def2-TZVP) = -553.36027291

2 1

Ru	-0.000004304091	-0.000022888024	-0.000035853048
O	-1.310847451856	1.565158966681	0.624584448420
O	-1.384777877332	-0.239683605374	-1.609501925893
O	1.310826214147	-1.565221250279	-0.624682325157
O	1.384795100255	0.239673691866	1.609381863926
O	1.342231305995	1.278396057224	-1.060696242885
O	-1.342233488691	-1.278406421540	1.060671993004
H	-1.928838929656	-1.781492035143	0.469224421144
H	-0.916910185437	2.427971380777	0.836991673424
H	-1.958100462391	0.537694438455	-1.728651141441
H	1.867792531121	-1.310687024933	-1.380999116917
H	1.957997433071	-0.537738130055	1.728803227971
H	1.929115867689	1.781043323299	-0.469136488366
H	-1.867959069208	1.310531173051	1.380779822960
H	-1.026875906114	-0.467686002809	-2.484010612410
H	0.916876754420	-2.428071525143	-0.836905190584
H	1.027065219177	0.468148984659	2.483815605805
H	0.962240976871	1.911963485747	-1.692148351334
H	-0.962310647133	-1.911537096491	1.692571399361

$[Co(NH_3)_6]^{2+}$

E(Def2-SV(P)) = -1721.45672100

G(Def2-SV(P)) = -1721.26754069

E(Def2-TZVP) = -1722.12019000

2 4

Co	-0.004203599558	0.008610248212	0.003543233768

N	1.118954556260	-1.258047588373	-1.406973855238
H	2.097484118322	-0.969298936285	-1.490028484210
H	0.735991409995	-1.237172059587	-2.355921264627
H	1.140762433692	-2.244864369484	-1.135657066812
N	1.712275197410	-0.072472897030	1.376360193040
H	2.515972534123	0.479166029695	1.064552365685
H	2.056457180724	-1.031250739541	1.482042844703
H	1.492136692195	0.251852210977	2.321810666829
N	-1.042963832495	1.338193820273	1.417384795747
H	-1.459373443230	0.838046730235	2.207174914423
H	-1.806906288701	1.864580133179	0.984464298080
H	-0.416127062212	2.038285594280	1.822876147839
N	0.802380644030	1.786854820741	-1.006701335282
H	1.482025480805	2.303145879926	-0.442046759864
H	0.060615306048	2.456333355006	-1.230690568722
H	1.267546822964	1.577201162322	-1.893880480785
N	-1.710212649272	-0.058728964768	-1.389588688679
H	-1.903633259667	-1.018641634896	-1.688223776823
H	-1.559731351870	0.478536332400	-2.247804800841
H	-2.584210474979	0.290130727007	-0.986931089497
N	-0.932654977369	-1.709126079236	1.031349571734
H	-0.585415907399	-1.845357909284	1.984711491873
H	-0.795287485749	-2.601675564213	0.549594029270
H	-1.946088830087	-1.591060212427	1.114506549631

$[Ru(NH_3)_6]^{2+}$

E(Def2-SV(P)) = -433.74130385

G(Def2-SV(P)) = -433.54287745

E(Def2-TZVP) = -434.23945955

2 1

Ru	0.000176293369	-0.009434897836	0.006823578649
N	1.252567851984	-1.216480130959	-1.291026202138
H	2.242045808348	-1.189637863599	-1.027947315155
H	1.219781768005	-0.914062170892	-2.268747163702
H	1.000351925649	-2.208720041343	-1.302648626160
N	1.189522708947	1.738981420857	-0.478867791198

H	0.734627395135	2.363725137574	-1.150593656576
H	2.100720370956	1.511190813268	-0.886780769570
H	1.390303771843	2.319039205681	0.340976537814
N	-1.242825928659	1.219016086916	1.293911336185
H	-0.829212889524	1.387574552464	2.215256012407
H	-2.165385945386	0.813632601346	1.475974997055
H	-1.421690352892	2.147954863839	0.901423385621
N	-1.322064705982	0.410669984818	-1.661848872972
H	-0.840741240124	0.811756124015	-2.472011736478
H	-2.068457515227	1.071994973428	-1.429936551095
H	-1.793583087128	-0.428552142955	-2.010876470299
N	-1.199292507764	-1.743899260313	0.509993344368
H	-1.048086554996	-2.545134624542	-0.109593466939
H	-2.205733243598	-1.557688745886	0.472705590767
H	-1.018630551434	-2.090021018827	1.456562539809
N	1.321865990523	-0.450349041851	1.670866602643
H	2.097365740246	0.212134061502	1.761421757324
H	1.755923173653	-1.374735613639	1.593504394338
H	0.848978030419	-0.446421645462	2.579332870099

$[Ru(NH_3)_5py]^{2+}$

E(Def2-SV(P)) = -625.27008441

G(Def2-SV(P)) = -625.02154901

E(Def2-TZVP) = -625.96333331

2 1

Ru	0.893031786526	0.000474886699	-0.001410778198
N	0.911311375080	1.466601881197	1.593017307740
H	1.579379226159	1.238877213028	2.335032611604
H	0.004417760222	1.553657391434	2.062006025500
H	1.162029507732	2.411300634566	1.287078350039
N	0.901965787618	1.548223575568	-1.518175371162
H	1.650246634190	1.424144005106	-2.206533064625
H	1.025510786567	2.498210345515	-1.155246288963
H	0.033358250635	1.567040749914	-2.061370259205
N	0.904018195886	-1.463272502672	-1.598710481413
H	1.151332033711	-2.411161159150	-1.299359732721

H	1.570821886338	-1.232548029040	-2.341133880617
H	-0.004466991327	-1.544522437355	-2.065631178058
N	3.066816487901	0.001926849709	0.003016914659
H	3.471334600996	0.819032747735	-0.462977015119
H	3.469963548147	-0.815166727840	-0.464166997441
H	3.468380499668	0.002351007537	0.944927985218
N	0.911054457172	-1.549997168823	1.513042266014
H	0.077416281637	-1.520774110802	2.108020343757
H	1.706612400024	-1.474018020657	2.153380500806
H	0.957112856786	-2.503908326673	1.142840951200
C	-4.021688618176	0.000685001312	-0.000772285150
C	-3.306472121092	1.195784934974	0.005903147594
C	-1.917644257916	1.150182282639	0.006947100962
N	-1.218998071184	0.000134867414	0.001256392758
C	-1.918063379234	-1.149587195102	-0.003718964703
C	-3.306861430329	-1.194684402932	-0.005218270234
H	-5.114530859910	0.000852480418	-0.002211427912
H	-3.811438742075	2.163828526458	0.009986579157
H	-1.337638867132	2.076140699059	0.012174427856
H	-1.338526773846	-2.075670913745	-0.006169268803
H	-3.812138904804	-2.162568841489	-0.009711805047

$[Co(en)_3]^{2+}$

E(Def2-SV(P)) = -1953.46417136

G(Def2-SV(P)) = -1953.15577488

E(Def2-TZVP) = -1954.34396285

2 4

Co	0.002374750383	0.004387076986	0.000755485175
N	-0.387612516088	-1.838392986517	1.141229577720
N	1.236470721612	-1.412102426565	-1.140796549436
N	-1.807138003421	-0.299222990094	-1.211578305176
N	-1.421636651536	1.200045958293	1.168202520016
N	1.729402199424	0.611159027267	1.218515692777
N	0.675470899360	1.742112988852	-1.163293758646
C	0.057279107539	-2.978992764633	0.325432121376
C	1.378341013462	-2.643550405876	-0.347776956133

C	-2.793969695912	0.711915721009	-0.797430953771
C	-2.778113588236	0.848355342391	0.716596283824
C	2.098030836709	1.977645024066	0.813562301542
C	2.027432175820	2.096579778562	-0.700393524789
H	-1.625851179326	-0.220139334820	-2.214525251035
H	-2.195492269562	-1.236451364302	-1.081655564495
H	-1.266969322118	2.204577317620	1.055383906180
H	-1.328386784435	1.024029540240	2.170996733556
H	0.757560009604	-1.620503905096	-2.020951113548
H	2.157104526344	-1.062345284956	-1.412810702824
H	-1.356375592928	-1.973954485677	1.436491160323
H	0.154826750573	-1.803062203211	2.008494468316
H	2.524880484636	-0.017591936850	1.083891454941
H	1.531054930982	0.584637545755	2.220982870631
H	0.050056747446	2.544034779665	-1.056157874263
H	0.688196052331	1.542715904802	-2.165917152866
H	2.319489125312	3.112624422645	-1.014832882769
H	2.741966535768	1.395107845343	-1.161261253406
H	3.104451877875	2.257695705335	1.167841626976
H	1.382030587211	2.675767043499	1.277437292126
H	-0.712568067478	-3.172969913333	-0.439583542541
H	0.164238702089	-3.900540154272	0.922319391646
H	2.150091084287	-2.468181072783	0.419664998356
H	1.714929911307	-3.496306581055	-0.961190397990
H	-3.053388131035	-0.114163536804	1.177588905490
H	-3.527574620664	1.590679984257	1.038700068927
H	-2.515813492651	1.671674673970	-1.263158868776
H	-3.811467970968	0.463483470085	-1.143510691375

$[Ru(en)_3]^{2+}$

E(Def2-SV(P)) = -665.74721794

G(Def2-SV(P)) = -665.43219115

E(Def2-TZVP) = -666.46727108

2 1

Ru	0.000395579515	0.014936405775	-0.007077812290
N	-1.343995798137	1.330237485846	1.064572750287

N	-1.761380298845	-0.230825968638	-1.239745993011
N	0.775488115010	1.678227161278	-1.158445954814
N	1.716353680583	0.512118894379	1.220192529976
N	-0.530160301929	-1.729868713652	1.158253893499
N	1.158372867724	-1.468582460425	-1.078276710240
C	-2.723234283927	1.023415306558	0.627184220683
C	-2.738890770377	0.819426222760	-0.876527374038
C	1.756686599612	2.405215929474	-0.324895491123
C	2.601633092392	1.407385475752	0.444594453256
C	0.410533751470	-2.819184042630	0.815601821964
C	0.670395180916	-2.806627637181	-0.679463722234
H	1.240521245786	1.327959049702	-2.000004554341
H	0.075687287275	2.337947441164	-1.505064872344
H	2.259082371820	-0.284148365592	1.561803014759
H	1.404977910637	0.996354528040	2.066230422180
H	-1.552117199748	-0.168937598541	-2.238563958201
H	-2.198990432986	-1.148494214497	-1.121891395738
H	-1.149678691888	2.319562262083	0.887717512851
H	-1.282919516629	1.227393926305	2.080239034993
H	-1.487262290254	-2.047255104804	0.981079013241
H	-0.495972969169	-1.555726753209	2.165148738499
H	2.158323689318	-1.403395161768	-0.870830557098
H	1.093818515863	-1.375984157105	-2.094517787781
H	1.388096721976	-3.598581805566	-0.951188788317
H	-0.264703387498	-3.003165360830	-1.228194513119
H	0.026092051736	-3.803039046432	1.131811454242
H	1.351173378908	-2.641044507600	1.361073024854
H	-3.427990157865	1.821679048786	0.913996315991
H	-3.041808651375	0.099785879655	1.136521170500
H	-3.755176549511	0.563655674195	-1.219394429129
H	-2.444404503928	1.751344272257	-1.385203055133
H	3.319304954108	1.934112737948	1.095448516056
H	3.184035226401	0.788349422470	-0.256926828972
H	2.398804045294	3.064035914307	-0.932961468031
H	1.198164265518	3.043329846828	0.378949172714

$[Fe(bpy)_3]^{2+}$

E(Def2-SV(P)) = -2748.02366289

G(Def2-SV(P)) = -2747.58450044

E(Def2-TZVP) = -2749.80275749

2 1

Fe	-0.002700511509	0.001544107961	0.002538398494
C	-4.117442900169	-0.915096710833	2.242153296239
C	-1.002085480039	1.901586111950	-2.079745419761
C	-1.950272066836	2.694998391866	-2.715172930717
C	-3.259215178521	2.673615925769	-2.243757071586
C	-3.571519384426	1.860885003440	-1.157971927185
C	-2.565804571580	1.092217519022	-0.574158910155
C	-2.782108839210	0.188097996541	0.578017179795
C	-4.027709247162	-0.046734676636	1.158201468081
C	-2.959324328758	-1.523700945168	2.715790302185
C	-1.753795592941	-1.240584693579	2.084176214974
H	0.033704670425	1.888047871356	-2.423996129377
H	-1.657362971019	3.315333886109	-3.563879263344
H	-4.590758460797	1.834047124607	-0.772460354243
H	-4.924921354772	0.436381242924	0.770997234612
H	-2.978873716810	-2.211070177551	3.563178947361
H	-0.823858871628	-1.695710698489	2.430046305680
N	-1.299282463048	1.119717997915	-1.037385189688
N	-1.664515439316	-0.407010738103	1.043314828839
C	1.943511479630	-0.892578066046	2.089323075585
C	2.785497692643	-1.796343101320	2.726964732440
C	2.838252376607	-3.103914536850	2.254233291734
C	2.048232165940	-3.459345166520	1.164955359609
C	1.227571940391	-2.496908135987	0.578963937831
C	0.340770011550	-2.761716893733	-0.576614755583
C	0.187726073725	-4.015051588149	-1.167384340202
C	-0.670393539602	-4.151465475462	-2.254610233805
C	-1.351431920150	-3.031096955108	-2.720443917586
C	-1.147076445806	-1.814885031214	-2.078992568945
C	-0.192020797914	2.142688359235	2.081894516929
C	0.176788297801	3.322692649922	2.717600347462

C	1.288822865020	4.013060622616	2.245455114334
C	1.989612249970	3.499224272987	1.158338443397
C	1.558858025501	2.309160014810	0.574174571310
C	2.227338295907	1.668251008801	-0.580844284422
C	3.395534182472	2.151129433455	-1.168559478843
C	3.936871732852	1.473505014900	-2.257032757673
C	3.294514727409	0.332425780304	-2.727447539924
C	2.134176898494	-0.088225574812	-2.088033099663
H	1.871205113809	0.140285873485	2.435044946613
H	3.385038009940	-1.470554045032	3.578635993833
H	2.078358383407	-4.478351811273	0.779077914819
H	0.727844115498	-4.882215723113	-0.787074346592
H	-2.033865160534	-3.087802033373	-3.570171459982
H	-1.658440408946	-0.912771798793	-2.419856730646
H	-1.055241975309	1.570496244575	2.426800256394
H	-0.404259154857	3.685184616177	3.567265795654
H	2.861205903069	4.028119353969	0.772473138088
H	3.885697515540	3.045624083873	-0.784127572704
H	3.679127474346	-0.232239615469	-3.578402737072
H	1.600200166654	-0.975959268325	-2.431781349524
N	1.183439739311	-1.231316699371	1.043459124252
N	-0.322887038183	-1.680778063975	-1.035547904117
N	0.479625665244	1.646527394171	1.038434932870
N	1.612196689477	0.560270124610	-1.042549270989
H	-5.085087347912	-1.112485984047	2.708930649296
H	-4.033953255976	3.283666992303	-2.713644333035
H	4.852051202111	1.836719831643	-2.730020457438
H	1.611629169728	4.944713592540	2.715644419175
H	3.488023420624	-3.844398818870	2.726100032653
H	-0.803189171866	-5.125718317640	-2.730391295003

$[Co(bpy)_3]^{2+}$

E(Def2-SV(P)) = -2867.07050715

G(Def2-SV(P)) = -2866.63868222

E(Def2-TZVP) = -2868.86350428

2 4

Co	-0.004796973917	0.002681799058	-0.002492841212
C	1.430913286100	-4.194271019693	-2.101162534918
C	2.308667718550	0.101582788460	2.055190799831
C	3.518627878851	-0.252474375244	2.642277649468
C	4.181851742257	-1.375099763023	2.155312442903
C	3.615530877253	-2.100754297520	1.110075276684
C	2.395060059743	-1.683245920822	0.578233550104
C	1.707339136233	-2.388490485840	-0.539992075365
C	2.140200799099	-3.611230065284	-1.053902949557
C	0.308863103193	-3.545079143415	-2.607979151591
C	-0.061330469103	-2.331791116563	-2.037828265888
H	1.749516192257	0.976611963888	2.398371023647
H	3.926858910922	0.344915541325	3.459313107588
H	4.132532157861	-2.975426663979	0.715354176665
H	3.014289922022	-4.117287634350	-0.644005270082
H	-0.275758063422	-3.966680805839	-3.427469386076
H	-0.936682142573	-1.783082635000	-2.396789312887
N	1.766462436823	-0.595719759615	1.055966214310
N	0.620345868246	-1.773660364064	-1.036576491944
C	-1.981000021582	1.216144472971	-2.052361976595
C	-3.211506177669	1.501220362038	-2.634280546496
C	-4.338613795538	0.850527544542	-2.140741939916
C	-4.197433838316	-0.058290427484	-1.094780769724
C	-2.926678054863	-0.294108709103	-0.569076442993
C	-2.668066940049	-1.244303382776	0.549290481982
C	-3.641043853688	-2.100652728955	1.065731686364
C	-3.304335388172	-2.952280477596	2.115112297009
C	-2.007952603736	-2.929542945367	2.621107020826
C	-1.093992164379	-2.052420092040	2.047160815531
C	2.056718701431	1.090670111545	-2.044799265741
C	2.929872586577	2.006832996612	-2.621238708876
C	2.937252719568	3.308237598827	-2.127832881867
C	2.075032681777	3.647419736176	-1.087947935152
C	1.223715033650	2.671819879620	-0.568142499679
C	0.263521264841	2.932508737199	0.541163201555
C	0.016701736468	4.205345070692	1.056671907795

C	-0.900192880109	4.348202658119	2.095319750719
C	-1.547944370284	3.220680187503	2.591758243567
C	-1.251648455147	1.987994956271	2.020138022180
H	-1.065597356743	1.703057261279	-2.400416104906
H	-3.277576353243	2.220651792071	-3.452309150704
H	-5.076027783881	-0.564460797861	-0.694505679884
H	-4.651054862184	-2.118567986355	0.656035229481
H	-1.702098518335	-3.579580045913	3.442689447454
H	-0.061918183523	-1.999295583811	2.405063288153
H	2.015380063406	0.054758947737	-2.393035301405
H	3.588615419054	1.698844527722	-3.435049458321
H	2.080742642113	4.661569716553	-0.688269457032
H	0.521021342721	5.084105124095	0.654459523559
H	-2.273330664943	3.288013082451	3.404400804110
H	-1.735446655114	1.072051172867	2.371100853343
N	-1.847243340448	0.343962834774	-1.052339548494
N	-1.418194580179	-1.236391107357	1.043357884391
N	1.230410051865	1.417283728361	-1.050210708369
N	-0.371666079263	1.852652081177	1.027224364716
H	1.755706349800	-5.152401478941	-2.513749341243
H	5.138020525054	-1.687616605392	2.581690457841
H	-1.106368575465	5.337883273486	2.509655474091
H	3.611666372505	4.059534439725	-2.545399179686
H	-5.326717852684	1.048372136957	-2.562849593895
H	-4.053579013610	-3.630506418023	2.530203335351

$[Ru(bpy)_3]^{2+}$

E(Def2-SV(P)) = -1579.36709892

G(Def2-SV(P)) = -1578.92924469

E(Def2-TZVP) = -1581.00735538

2 1

Ru	0.003551721976	0.000655354421	-0.000006763313
C	-3.680337448550	2.201384425280	2.282173251457
C	0.488563138584	2.180373707645	-2.092778505275
C	0.288685975421	3.395128271246	-2.736070264843
C	-0.713408406091	4.239922943927	-2.267701577360

C	-1.476988149754	3.840221915940	-1.175575948762
C	-1.222297158310	2.606790264121	-0.576767210109
C	-1.977534007365	2.088272820200	0.587109176746
C	-3.036629370610	2.768960266437	1.187144264772
C	-3.248021980008	0.965044533860	2.753122646774
C	-2.187383900045	0.341968907834	2.108092247477
H	1.259506592987	1.484133984012	-2.428677519146
H	0.912984077068	3.664778728817	-3.589528579443
H	-2.264481475890	4.491471328440	-0.796438826411
H	-3.363777531183	3.736658493682	0.806835604327
H	-3.720076757658	0.481113560232	3.609753831717
H	-1.810995633323	-0.625633774943	2.445741018005
N	-0.246399032792	1.793850579552	-1.042563323313
N	-1.567585912041	0.886595373236	1.053735619572
C	0.803220492033	-2.088646829952	2.089383534211
C	0.786035178195	-3.321883548990	2.728277629636
C	-0.080924231193	-4.303847483267	2.257698989774
C	-0.896938989883	-4.017216183412	1.168034337126
C	-0.828270562968	-2.757448949117	0.573773403719
C	-1.654384199273	-2.352050483567	-0.586693255056
C	-2.604833468927	-3.179271052410	-1.184200157735
C	-3.330422836289	-2.709770239596	-2.274387944922
C	-3.086986052532	-1.421811503691	-2.742548426302
C	-2.125972659746	-0.651511262775	-2.100303601527
C	1.406172427045	1.727593512681	2.101854711123
C	2.479985513645	2.332657978573	2.742290450722
C	3.764753070102	2.083161340851	2.268603241756
C	3.928262164988	1.239222059825	1.174744781724
C	2.805604888890	0.665417080464	0.578731650457
C	2.871116971974	-0.247948479524	-0.585382866182
C	4.064200187856	-0.652090495371	-1.183852749375
C	4.023305642991	-1.513604670384	-2.275454682622
C	2.787789999158	-1.950954603015	-2.743983942148
C	1.638602510857	-1.508771913715	-2.101199153755
H	1.462806075266	-1.286993882063	2.427308925102
H	1.444615745731	-3.499299665552	3.580138578270

H	-1.580012784338	-4.776232478337	0.786805702670
H	-2.784042323406	-4.185248332123	-0.805062169624
H	-3.629625103025	-1.009593892301	-3.594900252616
H	-1.899372260397	0.362304505103	-2.436095964532
H	0.381627726350	1.890599731691	2.441869891677
H	2.301462170859	2.986348823796	3.597677484853
H	4.928071811255	1.034084991435	0.792281313194
H	5.024068144458	-0.301651888826	-0.804621012706
H	2.704706471453	-2.626475571792	-3.596993184866
H	0.648022305724	-1.822072058194	-2.436777470764
N	0.017836350607	-1.811054921018	1.041129603988
N	-1.426935576325	-1.102040658893	-1.050966594752
N	1.561985686786	0.915428892262	1.048858662770
N	1.676422853774	-0.679128154634	-1.050992158888
H	-4.511495121358	2.723695099074	2.761127466538
H	-0.902395835199	5.203489128951	-2.746065972514
H	4.950454176929	-1.838193138416	-2.753016339785
H	4.635684737567	2.539623783573	2.744153476895
H	-0.124712207705	-5.286698197709	2.732174835094
H	-4.078165824524	-3.347280960722	-2.751335536120

4. Cartesian coordinates and energies of SX geometries obtained from the initial SX geometry generation at the UωB97X-D/Def2-SV(P) level

The initial SX geometries and energy components are presented below. These energy components are:

$$\Delta E_{1}^{MM} = \left(E_{n+m+1}^{\mathrm{C}\text{-}MM\text{-}Initial} - E_{n+1}^{D\text{-}MM} - E_{m}^{A\text{-}MM}\right) - \left(E_{n+m}^{\mathrm{C}\text{-}MM} - E_{n}^{D\text{-}MM} - E_{m}^{A\text{-}MM}\right)$$

$$\Delta E_{2}^{MM} = \left(E_{n+m+1}^{\mathrm{C}\text{-}MM\text{-}\mathrm{Final}} - E_{n}^{D\text{-}MM} - E_{m+1}^{A\text{-}MM}\right) - \left(E_{n+m}^{\mathrm{C}\text{-}MM} - E_{n}^{D\text{-}MM} - E_{m}^{A\text{-}MM}\right)$$

$$E_{1}^{'} = E_{n+m}^{\mathrm{C}} - E_{n}^{\mathrm{D}} + E_{n+1}^{\mathrm{D}} + \Delta E_{1}^{MM}$$

$$E_{2}^{'} = E_{n+m}^{\mathrm{C}} - E_{m}^{\mathrm{A}} + E_{m+1}^{\mathrm{A}} + \Delta E_{2}^{MM}$$

In the following, energy values at the UωB97X-D/Def2-SV(P) level are given in Hartree.

$[V(H_2O)_6]^{3+/2+}$

ΔE_1^{MM} = -0.01410198 = (2788.86751406 - 1393.98382572 - 1394.69624301)
- (2789.02029933 - 1394.12250901 - 1394.69624301)

ΔE_2^{MM} = -0.01278579 = (2788.96714950 - 1394.12250901 - 1394.65587897)
- (2789.02029933 - 1394.12250901 - 1394.69624301)

E'_1 = -2803.30972991 = (-2803.08550116) - (-1401.54173731) + (-1401.75186408) + (-0.01410198)

E'_2 = -2803.30972402 = (-2803.08550116) - (-1401.54012910) + (-1401.75156618) + (-0.01278579)

G(Def2-SV(P)) = -2803.06978602

V	-2.677379515715	-0.066131263419	-0.060739331285
O	-1.183698986749	-1.409341655657	-0.855464564496
O	-2.542195748090	-1.117972289537	1.713635273232
O	-4.093104490340	1.242879871059	0.650693272583
O	-2.735025339656	0.956057766164	-1.856116522669
O	-1.140264235641	1.111727134822	0.589108578086
O	-4.203042673452	-1.258239215631	-0.757469807907
H	-4.191226102674	-2.219470183888	-0.912916156090
H	-1.337982255126	-1.811333584478	-1.734059339856
H	-3.099721747666	-1.887347141004	1.928359794870
H	-4.433023991378	1.977241365788	0.108422560687
H	-3.091871568753	0.571340666432	-2.676412340977
H	-1.204303600663	2.071887659824	0.735287909893
H	-0.933467372300	-2.138818080787	-0.252809357021
H	-2.235239623863	-0.719908444879	2.547860841450
H	-4.228301212595	1.458261377032	1.590498360645
H	-2.139248495473	1.689882946564	-2.089345083394
H	-0.287472320968	0.787287411799	0.974770231701

H	-5.128782049716	-0.954700532705	-0.762318242006
V	2.672290522087	0.120317552525	0.044332162557
O	4.086139867670	0.127445185011	-1.455205901992
O	2.603216151699	-1.948110452985	0.155932912182
O	1.220915760669	0.166055257375	1.654001048811
O	2.703628126180	2.176971809464	-0.051917366005
O	4.281929348423	0.086375119738	1.324642201312
O	1.090888172264	0.065643747704	-1.241510108536
H	1.053237299072	0.524411666263	-2.098716900225
H	4.157068953647	0.859971857233	-2.093080502486
H	2.171238863773	-2.512085345953	-0.509982934323
H	1.388640183455	0.773725998731	2.402107211409
H	1.927072451113	2.750355397753	-0.179359643916
H	4.308018119392	-0.161569002281	2.265826161862
H	4.421250848406	-0.683800169380	-1.877030497223
H	3.318429564146	-2.462466188391	0.571859988897
H	1.021225742160	-0.709077653102	2.044406394686
H	3.452559758683	2.724876104862	0.243038143494
H	5.192165214856	0.227533534603	1.007491032331
H	0.255573288643	-0.454152543463	-1.123170109254

$[Cr(H_2O)_6]^{3+/2+}$

ΔE_1^{MM} = -0.01460320 = (2756.32818219 - 1378.41756027 - 1377.61270435)
- (2756.49097123 - 1378.56574611 - 1377.61270435)

ΔE_2^{MM} = -0.01457846 = (2756.30880476 - 1378.56574611 - 1377.44511635)
- (2756.49097123 - 1378.56574611 - 1377.61270435)

E'_1 = -3004.15405798 = (-3003.93001608) - (-1501.96131572) + (-1502.17075442) + (-0.01460320)

E'_2 = -3004.15405946 = (-3003.93001608) - (-1501.96187526) + (-1502.17134018) + (-0.01457846)

G(Def2-SV(P)) = -3003.91415195

Cr	-2.656543978072	-0.350718209000	0.013503254378
O	-1.034647989367	0.097864654531	-1.482056448435
O	-2.292967766162	-2.334913957495	-0.125985669523
O	-4.260579816119	-0.663753961371	1.346034585286
O	-2.906728196889	1.651558168671	0.029906148102
O	-1.239303896758	-0.135829521591	1.419175714664
O	-4.118488004141	-0.459986455705	-1.382840211199

H	-4.013828170665	-0.621247498596	-2.338179622896
H	-1.280800725559	0.748839193728	-2.169429782454
H	-2.833941920400	-2.925033763124	-0.681814541423
H	-4.611607670588	0.062872511719	1.893099171606
H	-3.707724978138	2.084328360463	-0.317678131815
H	-1.471929524545	-0.060678617254	2.362108204589
H	-0.669512058657	-0.678144822427	-1.952426060768
H	-1.880972314010	-2.860695103355	0.583549426631
H	-4.323390682378	-1.486832332535	1.864589251409
H	-2.480216351231	2.231774136934	0.686176385919
H	-0.325548461041	-0.528200259828	1.349877032815
H	-5.000853530538	-0.762187315272	-1.098340155995
Cr	2.602936767348	0.410870983190	0.556887281691
O	3.956215776683	1.920295311046	-0.014777027883
O	2.830013624503	-0.646432748401	-1.154513195759
O	1.217281816344	-1.215341185613	1.259031136159
O	2.346565409728	1.435047430055	2.286400637937
O	4.235774914240	-0.472105554334	1.363181832319
O	0.975722069002	1.322972450937	-0.179961704479
H	0.996429946031	2.258077997144	-0.450521319164
H	3.941850816400	2.767694517253	0.466677261474
H	2.496750094209	-0.325456279823	-2.012690868101
H	1.366135992750	-1.576676397198	2.156152495221
H	1.487592879615	1.819346806952	2.540030194282
H	4.341034572374	-1.404646481822	1.624393738313
H	4.166714287900	2.103897874904	-0.948282616465
H	3.627385251011	-1.184442642040	-1.312415092529
H	1.184658146576	-1.984380657241	0.654736183204
H	2.832647319382	1.180579790284	3.092062219525
H	5.112466800650	-0.084858235357	1.183204561961
H	0.229656252459	0.867123390065	-0.658991586609

$[Fe(H_2O)_6]^{3+/2+}$

ΔE_1^{MM} = -0.01346685 = (2774.37970420 - 1386.23933161 - 1387.91193106)

- (2774.42529675 - 1386.27145731 - 1387.91193106)

ΔE_2^{MM} = -0.01335348 = (2774.40223481 - 1386.27145731 - 1387.90222260)

- (2774.42529675 - 1386.27145731 - 1387.91193106)

E'_1 = -3442.57740070 = (-3442.30941512) - (-1721.15196186) + (-1721.40648059) + (-0.01346685)

E'_2 = -3442.57739801 = (-3442.30941512) - (-1721.15179503) + (-1721.40642443) + (-0.01335348)

G(Def2-SV(P)) = -3442.34028312

Fe	-2.653830416987	-0.020018274619	-0.232500451317
O	-1.079227878546	1.424251350616	-0.721779128396
O	-2.390267485978	-0.903051043506	-2.058207467770
O	-4.208116336598	-1.246376208450	0.320970715337
O	-2.867401326607	0.999552062281	1.532535026310
O	-1.204230490743	-1.211090174132	0.551426726754
O	-4.076098183356	1.235346888926	-1.042131750990
H	-3.969440904450	1.901149202940	-1.745085843216
H	-1.255282221832	2.370960356903	-0.551557977484
H	-2.923111939107	-0.686897770999	-2.845005769484
H	-4.550391762166	-1.238260512320	1.233340791711
H	-3.447941167897	1.775279003516	1.635839511593
H	-1.319371334835	-1.768443195061	1.340887562346
H	-0.725380868329	1.363322874473	-1.631830152540
H	-1.941985079651	-1.754865556512	-2.209658449104
H	-4.394674983034	-2.116970053497	-0.075525199592
H	-2.297732256195	0.922452684743	2.318955836842
H	-0.304276617319	-1.385945488927	0.168301941683
H	-5.024991442594	1.037069127326	-0.943289348535
Fe	2.614223786779	-0.270923254579	0.505823757061
O	3.910086418297	0.921369223449	1.566625173981
O	2.901392665263	0.809724182482	-1.208270113526
O	1.244321677240	-1.583377793984	-0.591438514974
O	2.308306607928	-1.441275737348	2.156996941776
O	4.232471771416	-1.466743275341	0.060548389513
O	0.988426120526	0.863100172513	0.947452541492
H	0.881428626741	1.365908055113	1.773777278993
H	3.986051214599	0.874721294481	2.536887742579
H	2.452973885982	1.645092281024	-1.433157964136
H	1.404321405846	-2.547654479860	-0.558581476614
H	1.540944182382	-1.414935422438	2.756359001181
H	4.371741992750	-2.046339045412	-0.709707555635

H	4.203401248378	1.801844262518	1.270360684437
H	3.640653440284	0.673468874046	-1.828263882095
H	1.132243061513	-1.351016817503	-1.535081286237
H	2.847245379812	-2.225878970213	2.364249226948
H	5.093864669651	-1.303225276802	0.485902732651
H	0.219362801841	1.064567615147	0.351729844467

$[Co(H_2O)_6]^{3+/2+}$

ΔE_1^{MM} = -0.01380583 = (2760.68323729 - 1380.38217056 - 1380.05429515)
- (2760.96441481 - 1380.64954226 - 1380.05429515)

ΔE_2^{MM} = -0.01346286 = (2760.71229661 - 1380.64954226 - 1379.81563980)
- (2760.96441481 - 1380.64954226 - 1380.05429515)

E'_1 = -3680.61588511 = (-3680.29905441) - (-1840.14684551) + (-1840.44987038) + (-0.01380583)

E'_2 = -3680.61588695 = (-3680.29905441) - (-1840.14687274) + (-1840.45024241) + (-0.01346286)

G(Def2-SV(P)) = -3680.37224723

Co	-2.638734439171	-0.046474449081	-0.060421456864
O	-1.210887113487	0.159879783727	-1.671175986674
O	-2.511393902548	-2.050938463443	-0.213872424892
O	-3.862101737813	-0.281199581425	1.565790921501
O	-2.651263157350	1.963787023502	0.079552328132
O	-1.098997574629	-0.058647653324	1.223965171563
O	-4.162015138564	-0.072100208951	-1.375018279769
H	-4.218265417652	0.418986314229	-2.214885648038
H	-1.001922440668	1.090874768214	-1.887970349573
H	-2.857790336146	-2.570038538039	-0.962913727877
H	-4.449707663332	0.439699889921	1.856825940555
H	-3.277019039216	2.554092448168	-0.379581819310
H	-1.211362447999	-0.393035349181	2.131645626857
H	-1.394960984806	-0.289634349860	-2.520018837766
H	-1.853644161895	-2.585030699512	0.268367543176
H	-4.333559666058	-1.125291072085	1.688390484942
H	-2.275396736159	2.436372848324	0.845238396583
H	-0.248887492855	0.452219417715	1.166392059610
H	-5.022263060130	-0.499217498762	-1.209093006050
Co	2.626160145029	-0.009274064082	0.061895873188
O	3.984550348205	-1.285620937525	-0.778881562181

O	2.533274765520	-1.236891683890	1.662679675612
O	1.224342231175	1.335298721253	0.994660305348
O	2.631364435066	1.295400703748	-1.481523081943
O	4.183041489150	0.922066103145	0.946603984218
O	1.082726661699	-0.963816547131	-0.776266960095
H	1.106137225803	-1.901095758587	-1.037803189043
H	4.007395645879	-1.470653805201	-1.735004125614
H	1.813751482804	-1.875672504706	1.819119059615
H	1.023448287320	2.120606836815	0.446848001742
H	2.240973394917	1.092239403751	-2.351663146458
H	4.249593277062	1.852858026566	1.226418222445
H	4.199401484122	-2.109001283682	-0.303403128094
H	2.967547427159	-1.031074800515	2.511074273148
H	1.405786610265	1.661892850176	1.898343718134
H	3.338702253182	1.955527066305	-1.604568054727
H	5.079521071220	0.541819766939	0.902735493755
H	0.236791720753	-0.560440702180	-1.111049005309

$[Ru(H_2O)_6]^{3+/2+}$

ΔE_1^{MM} = -0.01312151 = (2752.83764279 - 1372.38694452 - 1380.24644281)
- (2752.87733592 - 1372.41351614 - 1380.24644281)

ΔE_2^{MM} = -0.01200464 = (2752.81050307 - 1372.41351614 - 1380.19161461)
- (2752.87733592 - 1372.41351614 - 1380.24644281)

E'_1 = -1105.14058262 = (-1104.89962492) - (-552.44798991) + (-552.67582610) + (-0.01312151)

E'_2 = -1105.14058092 = (-1104.89962492) - (-552.44643726) + (-552.67538861) + (-0.01200464)

G(Def2-SV(P)) = -1104.88413656

Ru	-2.690204144839	0.047992392758	-0.151650344202
O	-1.149408382141	-0.497509934512	-1.552329776293
O	-2.478412253974	-1.981093662035	0.411236030592
O	-4.213034871297	0.569160079438	1.171990152981
O	-2.875688206545	2.069154529986	-0.714089111746
O	-1.158085140747	0.609428386353	1.105144616966
O	-4.248182351327	-0.373753324917	-1.459211749699
H	-4.232058903822	-1.105394717949	-2.102977113829
H	-1.321011870203	-0.282506701626	-2.491260584312
H	-3.210500492305	-2.583414783055	0.181530136677

H	-4.338401299378	1.530254205675	1.289552454351
H	-3.243752894572	2.226525081420	-1.604252094536
H	-1.327148273897	1.145436403213	1.899810888638
H	-0.986216397994	-1.462212904323	-1.505041067849
H	-2.242704797540	-2.163968902394	1.339763080870
H	-4.270177652022	0.139484323246	2.045178699734
H	-2.065384537347	2.606210416870	-0.633464163979
H	-0.344462658623	0.059537582370	1.250415619610
H	-5.151585133347	-0.287579599807	-1.099821497430
Ru	2.673165374671	0.004911489959	0.164853736661
O	4.188777550001	0.801677860806	-1.040430786273
O	2.786268127822	-1.617822232256	-1.152087556285
O	1.143616522321	-0.791079843093	1.489658419746
O	2.577079085379	1.623492067318	1.479562409549
O	4.253314770639	-0.718564962463	1.308411915231
O	1.135527833216	0.898630774871	-0.914697089200
H	1.373845337908	1.470041953037	-1.665844339928
H	4.293837804828	1.769351777393	-1.094948796457
H	1.998775947961	-2.148482657057	-1.368217349327
H	1.354992246912	-0.615279001279	2.429399817689
H	1.926457208389	2.320332025923	1.272685463592
H	4.221980892876	-1.584100314011	1.755408440933
H	4.238351226383	0.445668965083	-1.947847773376
H	3.545383923084	-2.226140375221	-1.081350855630
H	0.962459271217	-1.749936918477	1.421308760595
H	3.399078906173	2.050177426802	1.784724912072
H	5.145978413076	-0.584113573810	0.938159274863
H	0.322423556131	0.389183279787	-1.159708901991

$[Co(NH_3)_6]^{3+/2+}$

ΔE_1^{MM} = -0.00292513 = (3260.86599396 - 1629.95474305 - 1630.90778227)
- (3261.35409649 - 1630.43992045 - 1630.90778227)

ΔE_2^{MM} = -0.00291342 = (3260.85961495 - 1630.43992045 - 1630.41621415)
- (3261.35409649 - 1630.43992045 - 1630.90778227)

E'_1 = -3442.69936436 = (-3442.51023468) - (-1721.25767356) + (-1721.44387810) + (-0.00292513)

E'_2 = -3442.69936445 = (-3442.51023468) - (-1721.25766074) + (-1721.44387709) + (-0.00291342)

G(Def2-SV(P)) = -3442.27490070

Co	3.568130782296	0.133968334103	0.002149204446
N	3.603009749406	1.698058524800	-1.379235978629
H	3.284634302783	2.591652205703	-0.991641962276
H	3.021335060349	1.527697607166	-2.205775831212
H	4.548051632295	1.874375912105	-1.735525587529
N	2.299293059102	1.199624812457	1.270830274133
H	1.596809012181	0.588361828029	1.699054280127
H	1.769344095065	1.939765103777	0.800398921546
H	2.785586241610	1.653441590891	2.050461640323
N	3.639395938895	-1.432003198350	1.385937480205
H	2.983468593978	-2.192794761360	1.182885311929
H	3.437041487997	-1.137796710179	2.346877657558
H	4.565906233196	-1.869193113799	1.417060018084
N	1.873600556772	-0.648162936628	-0.938017832711
H	1.196743311631	0.073878211529	-1.203285340212
H	1.355500111886	-1.311164205474	-0.353430029368
H	2.094362129090	-1.154523902161	-1.801101206341
N	4.762578172921	-1.024771502709	-1.262852004766
H	4.738846671379	-0.712677975164	-2.238753458929
H	4.473798005961	-2.008351439883	-1.276594333058
H	5.752556130876	-1.032860006554	-0.998669170441
N	5.231729759436	0.983309578661	0.938043636839
H	5.135346979414	1.993569347766	1.081274992852
H	6.102581198758	0.863647063271	0.411294649921
H	5.413832774822	0.584430723456	1.864287759871
Co	-3.567658897670	-0.129317564957	-0.002855939139
N	-3.198616907967	1.011823993259	-1.714390146482
H	-4.068970930156	1.299449232609	-2.172870869691
H	-2.657624192316	0.519930910424	-2.432359007662
H	-2.684854626054	1.878241230377	-1.524643898606
N	-4.833683859855	1.387044209849	0.677141529952
H	-5.825896143347	1.202527779216	0.499986728308
H	-4.640644347632	2.293280169038	0.239065308279
H	-4.759324679349	1.541601020933	1.687618260189
N	-4.040956711215	-1.235022644787	1.700992721871

H	-3.745214442637	-2.214863761191	1.647965895559
H	-5.051551322830	-1.256981017779	1.872223434223
H	-3.624027641148	-0.860915802044	2.559044792021
N	-5.112623344926	-1.072297744349	-1.049017692972
H	-5.945489277232	-0.489704996266	-1.180187465089
H	-5.443189345333	-1.927965622341	-0.592063655504
H	-4.823780445890	-1.353647312975	-1.991345608050
N	-2.258308516902	-1.640398017670	-0.614370874865
H	-1.618857475020	-1.342205659591	-1.357421212359
H	-2.733256516549	-2.473416707394	-0.976280967088
H	-1.658793022120	-1.970667034878	0.148344105555
N	-1.963451454813	0.774494653141	0.985627892681
H	-2.213736857961	1.647496334994	1.460536508141
H	-1.202975590717	1.017590033873	0.343545591274
H	-1.538785412577	0.177086922139	1.701535790343

$[Ru(NH_3)_6]^{3+/2+}$

ΔE_1^{MM} = -0.00295088 = (3252.58414206 - 1625.07077183 - 1627.51019818)

- (3252.51122754 - 1624.99490643 - 1627.51019818)

ΔE_2^{MM} = -0.00293668 = (3252.54923814 - 1624.99490643 - 1627.55114545)

- (3252.51122754 - 1624.99490643 - 1627.51019818)

E'_1 = -867.29049198 = (-867.09789410) - (-433.55103603) + (-433.74068302) + (-0.00295088)

E'_2 = -867.29049195 = (-867.09789410) - (-433.55118214) + (-433.74084330) + (-0.00293668)

G(Def2-SV(P)) = -866.86696131

Ru	3.510336687685	0.088039151964	-0.128177149946
N	4.508883710001	-0.076820897807	-2.021402624646
H	3.937195943539	0.265516155872	-2.801171150457
H	4.752723310637	-1.041195764504	-2.268581359037
H	5.385427635753	0.452470997903	-2.068407228127
N	2.645396715292	1.971233119596	-0.710021871790
H	1.630820229881	1.937711807511	-0.849394323016
H	3.032043228522	2.347358296675	-1.581725289447
H	2.800644898215	2.701810883817	-0.007324473225
N	2.485785298044	0.234878107929	1.757982858716
H	1.945848109314	-0.604076296896	1.993564907061
H	1.817730205310	1.011526115660	1.790411708824

H	3.113710808554	0.384374049023	2.554841260912
N	1.846345972985	-1.008811585604	-0.938380389580
H	1.225182768746	-0.434557313039	-1.517277934641
H	1.243948086103	-1.421839671324	-0.219668754391
H	2.135158270420	-1.786658165310	-1.540226927411
N	4.390625364907	-1.768973670620	0.495407033154
H	5.284665970733	-1.976338625403	0.038477127657
H	3.790511135752	-2.580804530156	0.318578511821
H	4.585780659326	-1.792854289092	1.502165797280
N	5.196043669373	1.183486214492	0.640421660605
H	5.455231019638	1.975518009347	0.043165993177
H	6.043475514571	0.613731326308	0.728482002173
H	5.037139902810	1.587546394311	1.568971439288
Ru	-3.519245869589	-0.088959642997	0.130914770142
N	-3.995176864022	0.298067441937	-1.933016676946
H	-4.970615072371	0.582452362441	-2.068343391797
H	-3.856742704799	-0.518229925876	-2.537476719904
H	-3.432438861760	1.045964792380	-2.350878374693
N	-4.653279650395	1.640382285659	0.715572928694
H	-5.668149181030	1.510572448820	0.658430571209
H	-4.451515318241	2.454580220648	0.125048828047
H	-4.464627491118	1.944725444283	1.676224905982
N	-3.087981214784	-0.478805896091	2.201684097493
H	-2.721924960490	-1.420943723185	2.372790808070
H	-3.916018479541	-0.396790266044	2.800029128758
H	-2.393061857485	0.160775513405	2.599638857785
N	-5.272493453593	-1.323303613045	0.267489521644
H	-6.045653372304	-0.887845433144	0.780167856188
H	-5.091864108386	-2.207471734739	0.755119581545
H	-5.656573845278	-1.585325857337	-0.645924703084
N	-2.353399498253	-1.789835652151	-0.489070402525
H	-1.714603319505	-1.562807042933	-1.257860008038
H	-2.920254484234	-2.575160701389	-0.824604674661
H	-1.759567648785	-2.175283564785	0.251742731316
N	-1.734573581421	1.114768737954	0.021393399954
H	-1.838040407540	2.038525947759	0.453381921892

H	-1.406051752756	1.291428952887	-0.933060816016
H	-0.946296381942	0.673181660804	0.504000609341

$[Ru(NH_3)_5py]^{3+/2+}$

ΔE_1^{MM} = -0.00400124 = (3811.70168952 - 1904.96781037 - 1906.73872468)

- (3811.59593981 - 1904.85805941 - 1906.73872468)

ΔE_2^{MM} = -0.00400202 = (3811.69008612 - 1904.85805941 - 1906.83687302)

- (3811.59593981 - 1904.85805941 - 1906.73872468)

E'_1 = -1250.35041792 = (-1250.15074153) - (-625.07367618) + (-625.26935132) + (-0.00400124)

E'_2 = -1250.35041773 = (-1250.15074153) - (-625.07380680) + (-625.26948098) + (-0.00400202)

G(Def2-SV(P)) = -1249.82699624

Ru	-4.529621865167	-0.082186080665	0.018152907471
N	-4.077184017300	-1.483391546839	-1.546221804210
H	-4.358760319672	-2.441944744293	-1.316589328631
H	-3.075663129274	-1.545669654179	-1.754841890282
H	-4.535844279055	-1.275121761667	-2.438854705051
N	-6.523365105712	-0.876650460543	0.086956221223
H	-6.850194413006	-1.076142380209	1.037744527510
H	-6.618581844586	-1.755730065062	-0.431759130171
H	-7.223215093376	-0.250556901211	-0.324702586487
N	-3.949638726278	-1.555422543366	1.471186646386
H	-2.943655935805	-1.554279931153	1.666067680795
H	-4.161859784128	-2.515328186417	1.180218672208
H	-4.408304127785	-1.441175894461	2.380837446975
N	-5.043836452417	1.298641898047	1.582976020848
H	-4.506235819386	2.170651055593	1.530543472987
H	-4.906916192033	0.938542041944	2.532710616992
H	-6.027662364656	1.586455152945	1.549928473986
N	-5.165438182257	1.387678912946	-1.416217227432
H	-5.379612897777	1.012489734400	-2.345902939035
H	-4.465564777129	2.123348751210	-1.560489293767
H	-6.012717072907	1.886428310882	-1.125786695650
C	-0.045440388131	1.901761142613	-0.126764700167
C	-0.645929906615	1.588438915394	1.090007615298
C	-1.900989159785	0.996226112811	1.085702567324
N	-2.570940330326	0.714023943950	-0.048104115923

C	-1.976518664762	1.003935996216	-1.222179843221
C	-0.723425852687	1.594532020525	-1.304075755954
H	0.935431717534	2.381294911877	-0.158106600501
H	-0.153655684600	1.796412748327	2.041510948655
H	-2.389273878328	0.744368190982	2.029011484895
H	-2.526422228008	0.761170876477	-2.134093511043
H	-0.292884711775	1.807199078850	-2.283923727570
Ru	4.530079763643	0.086050139963	0.031643702242
N	6.530556977854	0.878795593051	0.126865977621
H	7.211704127449	0.332793441099	-0.410548731656
H	6.896686785138	0.915853191839	1.083412083367
H	6.606215016229	1.833938709684	-0.237856892531
N	5.110919496290	-1.241119551378	-1.559628205114
H	6.064780885013	-1.600844658912	-1.453020887466
H	5.090365135057	-0.812678347112	-2.490423420954
H	4.516974130411	-2.075058737056	-1.616165338284
N	5.094834782683	-1.431239123532	1.448574116988
H	5.993403777428	-1.867325404409	1.217235784461
H	4.422125260640	-2.204635855081	1.482066900554
H	5.199099907223	-1.102632478588	2.413929697154
N	3.987956672105	1.430364351102	1.618850158199
H	4.486551768166	2.324558088412	1.570276836182
H	4.173597513547	1.071851130777	2.560705212779
H	2.992888932223	1.675334526658	1.606828340923
N	4.028322670129	1.606228324755	-1.407254081292
H	4.692434009594	1.656040009207	-2.186834743630
H	4.003668876925	2.549711662614	-1.007421546389
H	3.108334354006	1.474323378885	-1.839030425558
C	0.047796504721	-1.907287806149	-0.089010166079
C	0.706190220292	-1.576740680171	-1.270855493147
C	1.958655141758	-0.982265523512	-1.197240585380
N	2.570676166001	-0.709558901792	-0.029409526447
C	1.918707254918	-1.013104043364	1.109219226604
C	0.666359879788	-1.611104369723	1.123140336726
H	-0.931522404838	-2.391011147021	-0.111863738635
H	0.262186604853	-1.775157690064	-2.247774712146

H	2.494913912588	-0.723600398327	-2.112534974118
H	2.420624179052	-0.773468545563	2.048427088019
H	0.190040665321	-1.836737075188	2.078807066536

$[Co(en)_3]^{3+/2+}$

ΔE_1^{MM} = -0.00276063 = (3931.63382252 - 1964.66871821 - 1966.96422761)
- (3931.68481840 - 1964.71695346 - 1966.96422761)

ΔE_2^{MM} = -0.00274591 = (3931.63289172 - 1964.71695346 - 1966.91504685)
- (3931.68481840 - 1964.71695346 - 1966.96422761)

E'_1 = -3906.71804502 = (-3906.52964351) - (-1953.26639181) + (-1953.45203269) + (-0.00276063)

E'_2 = -3906.71804498 = (-3906.52964351) - (-1953.26615655) + (-1953.45181211) + (-0.00274591)

G(Def2-SV(P)) = -3906.06488154

Co	-3.873543706532	0.067386073654	-0.012046321930
N	-1.836110727345	0.440231967345	0.274149141893
N	-4.071599912227	1.612115531453	1.383109374027
N	-3.936565501707	1.359794569962	-1.656336795930
N	-3.478028945329	-1.363543349036	-1.484070527367
N	-3.965119407180	-1.379583819498	1.492005680101
N	-5.937718378917	-0.256429266773	-0.085362712638
C	-1.679758375583	1.734541153837	0.968903246210
C	-2.761281157465	1.845800588953	2.024797437389
C	-4.054646069174	0.539397125160	-2.880509163367
C	-3.143563739002	-0.663418879846	-2.742721660837
C	-5.285036548973	-2.042467340922	1.424356327066
C	-6.338085786359	-0.993044263161	1.132054913703
H	-4.709410507640	2.029925250753	-1.613258575634
H	-3.093513334404	1.938275850006	-1.713866460320
H	-4.282377653849	-1.977907550325	-1.637915714143
H	-2.714800047357	-1.994445365956	-1.223812282585
H	-4.370893978665	2.461880155326	0.895886950340
H	-4.787907689378	1.458052699718	2.097418300719
H	-1.290421448649	0.436452971468	-0.589946230068
H	-1.429852016742	-0.306337111745	0.845881480145
H	-3.838413002308	-0.959361065866	2.417028652810
H	-3.224675257384	-2.082558615651	1.420339709146
H	-6.204820145382	-0.796387962792	-0.913129410639

H	-6.472814094332	0.613453636943	-0.159904650384
H	-7.330700244232	-1.455232852010	1.013514951208
H	-6.407608124231	-0.275321832828	1.964542766086
H	-5.513092294509	-2.578517498806	2.359053668468
H	-5.252300836511	-2.788092285519	0.614651907328
H	-1.783303900789	2.541872524337	0.227036058919
H	-0.680270243473	1.832250916122	1.420977114295
H	-2.615079411952	1.078081005025	2.801119549683
H	-2.725148686280	2.828310914310	2.521147199840
H	-2.090663694679	-0.343200776220	-2.692396699067
H	-3.241555421000	-1.331912548453	-3.612333331823
H	-5.102903658749	0.216294302468	-2.978942495498
H	-3.802395010154	1.121868222776	-3.780611263788
Co	3.891531311702	-0.069908494471	0.010985466454
N	5.332444552514	-1.331442827832	0.850889025043
N	5.484960600220	0.475389237110	-1.229805898164
N	3.221995753148	-1.582294462301	-1.265407181307
N	2.395626812097	-0.823719584378	1.263107684026
N	4.293321133399	1.506232947960	1.320258325210
N	2.604870700791	1.328703371503	-0.860082123512
C	6.499924535439	-1.393199349822	-0.053779318328
C	6.743673192462	-0.012676893421	-0.629241117826
C	1.897202639294	-2.030826647035	-0.788064635866
C	1.936219861460	-2.121676779649	0.724421071946
C	3.231604072329	2.523471804958	1.165950826970
C	2.908306173021	2.666466014977	-0.308285915167
H	3.155246469624	-1.280516020109	-2.240991197334
H	3.867532257340	-2.377170911725	-1.279782315058
H	1.601378948519	-0.183085297119	1.336332510527
H	2.724381110861	-0.939325153838	2.225437778444
H	5.357054603491	0.043759875897	-2.149929900762
H	5.557164503531	1.477890954110	-1.420384011232
H	4.999842657927	-2.277454300548	1.054537147633
H	5.623047902168	-0.957086859105	1.759084295198
H	5.204097463040	1.929824094632	1.122044401006
H	4.348582556277	1.210434523960	2.298581510502

H	1.625585084018	1.098151976449	-0.677317250695
H	2.679746789463	1.345002682555	-1.881020587598
H	2.068367049840	3.362612718424	-0.458886173190
H	3.774133237224	3.076511199423	-0.851039981823
H	3.539016502924	3.491281916393	1.592592942297
H	2.346476091946	2.184951688039	1.726700499067
H	6.273962536773	-2.106670543261	-0.861642037716
H	7.395385113850	-1.762617674329	0.470324960576
H	7.042789169559	0.689117360558	0.165383530198
H	7.559058675720	-0.037554060197	-1.369471691141
H	2.648572099606	-2.899444091051	1.041537404779
H	0.948194602322	-2.396314997025	1.126248563452
H	1.147574379376	-1.291607970711	-1.110199474063
H	1.615783075432	-2.999085647620	-1.231193835824

$[Ru(en)_3]^{3+/2+}$

ΔE_1^{MM} = -0.00272102 = (3919.45001371 - 1960.14005767 - 1959.30890249)

- (3919.27699143 - 1959.96431437 - 1959.30890249)

ΔE_2^{MM} = -0.00270202 = (3919.44005606 - 1959.96431437 - 1959.47466914)

- (3919.27699143 - 1959.96431437 - 1959.30890249)

E'_1 = -1331.30142475 = (-1331.10707623) - (-665.55489989) + (-665.74652740) + (-0.00272102)

E'_2 = -1331.30142478 = (-1331.10707623) - (-665.55494792) + (-665.74659446) + (-0.00270202)

G(Def2-SV(P)) = -1330.64877477

Ru	-3.907936323779	0.039055352007	-0.026228322528
N	-1.856398292366	0.579343685478	0.314169709045
N	-4.197263128601	1.546071335675	1.480039348281
N	-3.971327727454	1.410658467065	-1.680553092906
N	-3.458190410653	-1.328738588752	-1.620137250338
N	-3.960266515508	-1.470344985755	1.504802040104
N	-5.984739479441	-0.495978836245	-0.147062934160
C	-1.804548308398	1.847403627495	1.080928074957
C	-2.896969226539	1.838569585826	2.131471696632
C	-4.048146904346	0.634221045265	-2.942244298412
C	-3.118357309637	-0.557882311930	-2.841597012796
C	-5.219176305242	-2.244316175782	1.373614635964
C	-6.351993858943	-1.291245490255	1.050518621059

H	-4.766533171998	2.054229546338	-1.632015479156
H	-3.149743243320	2.021688182983	-1.710123434493
H	-4.239307278616	-1.957977296082	-1.826098154169
H	-2.681439658945	-1.953399086323	-1.385894303635
H	-4.558497749528	2.398746069815	1.041370214876
H	-4.891073607108	1.316010879633	2.195962971063
H	-1.293303709713	0.673103438730	-0.533936366211
H	-1.396514816152	-0.162709997549	0.849022144207
H	-3.906574986570	-1.068668471458	2.445479021203
H	-3.164373417473	-2.113156333994	1.454812700887
H	-6.197774829476	-1.039813987716	-0.988476641980
H	-6.595744563735	0.323519766101	-0.210717742040
H	-7.291059447137	-1.842734318086	0.886205335802
H	-6.519029353278	-0.593801040496	1.885750290875
H	-5.434105717052	-2.808849040858	2.294556083633
H	-5.086574736999	-2.972713291119	0.558661878662
H	-1.959478713816	2.678428634513	0.375287901303
H	-0.816392646406	1.988902048819	1.547031808316
H	-2.703713722341	1.051780659252	2.877650584830
H	-2.928751952305	2.801041556961	2.665855188864
H	-2.072217871331	-0.224447441977	-2.756287237784
H	-3.192995805759	-1.188945704094	-3.740867289249
H	-5.088765874073	0.298315000634	-3.072335951469
H	-3.788319955629	1.261278095694	-3.809540800134
Ru	3.912579234808	-0.035757615661	0.023214435901
N	5.390399593571	-1.387132807815	0.815558982568
N	5.508165325155	0.341135062806	-1.370027264551
N	3.054461696095	-1.655062899346	-1.098357994359
N	2.371885226191	-0.623708291956	1.400584855784
N	4.547754450789	1.647547938282	1.197789726440
N	2.611962409327	1.461427141163	-0.799388587289
C	6.475029726540	-1.559251411127	-0.181235868612
C	6.769766874394	-0.223247896446	-0.830903561701
C	1.739608615120	-2.008470006251	-0.508123346027
C	1.842676140211	-1.951732394137	1.002779786785
C	3.534375598428	2.723524512746	1.073785215240

C	3.080497297717	2.802295174540	-0.369509791365
H	2.925940837190	-1.429738740097	-2.089171034869
H	3.658105901335	-2.482298863079	-1.102822614870
H	1.602570360323	0.050929319121	1.435443816261
H	2.712404920326	-0.670696117695	2.365440147598
H	5.297079950499	-0.107697619848	-2.266894437463
H	5.654085991984	1.325506772870	-1.609035120084
H	5.038850654389	-2.307385706435	1.093738590137
H	5.782275461201	-0.993517976373	1.676899858095
H	5.460481029394	2.008699815679	0.905733466391
H	4.670558423336	1.412003877139	2.187036562800
H	1.639262287042	1.337806290728	-0.507252499914
H	2.576778869368	1.428165775060	-1.822304766623
H	2.286342356450	3.555475985350	-0.490032310464
H	3.918874642488	3.094786251326	-1.020735372003
H	3.940942831548	3.690331228433	1.409533118515
H	2.688283380492	2.469933884990	1.731516631310
H	6.132694654817	-2.283300728562	-0.936906313987
H	7.381799046388	-1.972423327732	0.287592795618
H	7.172657953709	0.484081490922	-0.089171247638
H	7.523622874205	-0.335619678419	-1.625936198134
H	2.537979143721	-2.720911200970	1.372892098871
H	0.862691682801	-2.141935816622	1.467920293964
H	0.999004504864	-1.279222848553	-0.869968733365
H	1.413825918709	-3.006836279456	-0.840053837530

$[Fe(bpy)_3]^{3+/2+}$

ΔE_1^{MM} = -0.00497619 = (5891.86548625 - 2946.21599025 - 2945.66463635)

- (5892.03809686 - 2946.38362467 - 2945.66463635)

ΔE_2^{MM} = -0.00494282 = (5891.83838080 - 2946.38362467 - 2945.46986310)

- (5892.03809686 - 2946.38362467 - 2945.66463635)

E'_1 = -5495.85915442 = (-5495.65176133) - (-2747.82011491) + (-2748.02253181) + (-0.00497619)

E'_2 = -5495.85915455 = (-5495.65176133) - (-2747.82017404) + (-2748.02262444) + (-0.00494282)

G(Def2-SV(P)) = -5494.96263975

Fe	-4.267531369281	0.669611604322	0.073403486406
C	-6.928587510489	-2.996049809512	1.504697354968

C	-2.688411687645	1.152916849902	2.549718793863
C	-2.241593306457	0.868737052537	3.833562361252
C	-2.769288696150	-0.238498855961	4.492539548666
C	-3.739938580401	-1.006731162789	3.856319777408
C	-4.150514706938	-0.652742648006	2.573298946636
C	-5.206348230103	-1.354246497838	1.814712571385
C	-5.940120596823	-2.429531936261	2.305920682113
C	-7.159532516322	-2.468289147482	0.238569924031
C	-6.386997814805	-1.394614974283	-0.187182972226
H	-2.300814847910	2.012202362887	2.000019222099
H	-1.488630362909	1.507692872504	4.297725134557
H	-4.171404057469	-1.870539182323	4.361778979248
H	-5.751213836597	-2.824416415268	3.304067133593
H	-7.925861898759	-2.876402824452	-0.422128780219
H	-6.532824422168	-0.958962466230	-1.176785978839
N	-3.611146146951	0.405933314462	1.933047455606
N	-5.431573153147	-0.857567787637	0.578670120677
C	-4.564972288612	0.431107741348	-2.890234121059
C	-5.112007841440	0.742385347173	-4.128305070084
C	-6.189316785997	1.620807764868	-4.179302790905
C	-6.685934401097	2.151060428404	-2.991921311700
C	-6.089413961878	1.791104400663	-1.786839777495
C	-6.535754400615	2.278016371689	-0.466163800677
C	-7.655606994520	3.080899347551	-0.268950562703
C	-7.990315031816	3.471150135101	1.024684771569
C	-7.197850205303	3.045209315842	2.085725288308
C	-6.096504021946	2.243578059156	1.814684857367
C	-2.736147175363	-1.806968559817	-0.602789754255
C	-1.686066326928	-2.522063916184	-1.164693087645
C	-0.614111611656	-1.818458532131	-1.705060147256
C	-0.620143597287	-0.428836751229	-1.641618244032
C	-1.701691845573	0.222748585964	-1.054268841035
C	-1.819416062956	1.690251435217	-0.934339031421
C	-0.835447022416	2.587410955381	-1.338248041862
C	-1.051309308644	3.952355038547	-1.166966771358
C	-2.247940404349	4.382217017588	-0.603306508255

C	-3.187877176270	3.429405982196	-0.228302736009
H	-3.724043763104	-0.259254590208	-2.815926941380
H	-4.690751542173	0.297132253528	-5.030850068912
H	-7.528516480057	2.841936028909	-3.012318941024
H	-8.270620501775	3.397124526375	-1.111322022228
H	-7.421296233596	3.323452017500	3.116747275068
H	-5.452703852438	1.894444710644	2.622892184899
H	-3.602214355571	-2.326775358955	-0.191692573540
H	-1.722171855486	-3.612126010548	-1.179018756879
H	0.206878753502	0.140447924538	-2.062026138020
H	0.095508403295	2.235256685060	-1.781883026822
H	-2.462152587407	5.440928241318	-0.450302548963
H	-4.136062701270	3.727170686013	0.222524730070
N	-5.039033905012	0.942776026237	-1.748486794056
N	-5.770867398274	1.873283543877	0.570799284399
N	-2.750012350286	-0.468925094637	-0.561349688809
N	-2.975633856265	2.118405042784	-0.383864688167
H	-7.514323030996	-3.841060625757	1.872821500301
H	-2.435843691160	-0.501005754027	5.498786666366
H	-0.286742993309	4.669452669228	-1.473520470381
H	0.221174216966	-2.346883804272	-2.170402747155
H	-6.644110369146	1.893747007809	-5.133975642468
H	-8.866128296063	4.100170092368	1.198037296184
Fe	4.271389037086	-0.670403518253	-0.073830936095
C	8.640245081870	0.872716597097	-1.138464308904
C	4.500960371385	-0.818369215225	2.900406010861
C	5.121479979255	-0.613025748824	4.126016318378
C	6.408886537577	-0.086454229717	4.141399074805
C	7.032756272943	0.209924280883	2.932768339023
C	6.350298601157	-0.024451429763	1.742268266513
C	6.912491954088	0.235548443585	0.401541544355
C	8.213010038722	0.675766677867	0.171757253474
C	7.756762831930	0.620658395777	-2.183086220892
C	6.473706380894	0.184047944292	-1.879277037216
H	3.495494977549	-1.238364764923	2.852970803810
H	4.593528471277	-0.866245807450	5.046598287988

H	8.041741953501	0.621765554293	2.925386588978
H	8.894393688956	0.860665533140	1.002028349325
H	8.046967276347	0.757285371014	-3.225918545105
H	5.753444561516	-0.017724827671	-2.672994558338
N	5.097865898214	-0.530328344009	1.738364287610
N	6.060262066314	0.002386511878	-0.619896916442
C	2.897349657928	0.108608655166	-2.595920703538
C	2.367090850943	-0.112176592833	-3.860430877412
C	2.528978085633	-1.367251445346	-4.441085568509
C	3.236347107559	-2.346879576148	-3.750432095740
C	3.751415705557	-2.049306514202	-2.490880269718
C	4.558728410980	-2.984268395140	-1.680167481442
C	4.940308629235	-4.255035621426	-2.099497096020
C	5.719918856385	-5.040556610390	-1.254021750423
C	6.099956117323	-4.532380211634	-0.016248104049
C	5.681063641252	-3.255316404012	0.337030892471
C	4.061678178882	2.300880114514	0.062780874449
C	3.453186898600	3.509360051819	0.381122788955
C	2.188864983582	3.486805830120	0.959942311223
C	1.577097647503	2.259340327584	1.200606927933
C	2.243403527286	1.089143828021	0.849700501759
C	1.691425705995	-0.267681329659	1.043196619959
C	0.463071373917	-0.534688937577	1.643082946575
C	0.040587878616	-1.853627028610	1.773569128433
C	0.853538834618	-2.872730060377	1.287393833484
C	2.070601517326	-2.534340320545	0.709799737024
H	2.792569248650	1.078895711066	-2.107718489922
H	1.833945005495	0.691384820782	-4.371102519696
H	3.383340413007	-3.330698028632	-4.195389415282
H	4.640403280254	-4.635108724697	-3.076087136949
H	6.712833792599	-5.110103654586	0.677281417317
H	5.953218684655	-2.825390850333	1.302378452562
H	5.051099763396	2.278633089781	-0.397382476665
H	3.972290236901	4.446170674398	0.173546145005
H	0.588759324523	2.224617141862	1.657862549078
H	-0.157667442807	0.276450874140	2.020004411125

H	0.560973669813	-3.921360816810	1.355134607796
H	2.741592985419	-3.310103590788	0.339227028482
N	3.560087085500	-0.839433568997	-1.924327940798
N	4.925874545701	-2.504613693598	-0.471740370358
N	3.469702192707	1.122634127225	0.285266424811
N	2.485117022464	-1.266084429535	0.603006101533
H	9.657351066696	1.217415797923	-1.337065905942
H	6.928913388285	0.092782977783	5.084890562369
H	-0.915938734541	-2.083055603036	2.248533332683
H	1.676610204909	4.414421484688	1.224464300671
H	2.116441786454	-1.582739959654	-5.428953763672
H	6.028319636919	-6.040671448987	-1.566304769989

$[Co(bpy)_3]^{3+/2+}$

ΔE_1^{MM} = -0.00651924 = (5893.93355501 - 2947.20758379 - 2946.74067446)

- (5893.89176814 - 2947.15927768 - 2946.74067446)

ΔE_2^{MM} = -0.00593131 = (5893.92332160 - 2947.15927768 - 2946.77815922)

- (5893.89176814 - 2947.15927768 - 2946.74067446)

E'_1 = -5733.94673429 = (-5733.76311764) - (-2866.87609765) + (-2867.05319506) + (-0.00651924)

E'_2 = -5733.94673528 = (-5733.76311764) - (-2866.87568249) + (-2867.05336882) + (-0.00593131)

G(Def2-SV(P)) = -5494.96263975

Co	-4.224691311278	0.561805332032	0.091775640689
C	-6.813372512285	-3.148729485464	1.446184210879
C	-2.699153214180	1.076148486099	2.674633305181
C	-2.269777835371	0.767552718724	3.960178762719
C	-2.771475445683	-0.379549986805	4.569745360676
C	-3.700642545410	-1.160932390456	3.887877049504
C	-4.096700938581	-0.775928894265	2.608184728447
C	-5.122812256049	-1.481993240976	1.809033690650
C	-5.850276303587	-2.573926737884	2.272107247202
C	-7.026438860616	-2.613563061014	0.180699548001
C	-6.262889021991	-1.523380543071	-0.218339579105
H	-2.330941694760	1.964921672635	2.157149588023
H	-1.550089506028	1.415730258429	4.462759662479
H	-4.108095888951	-2.056696955429	4.355997539075
H	-5.677653798399	-2.974962423301	3.270654878598

H	-7.771979223258	-3.026633467027	-0.500257956826
H	-6.394566378056	-1.079348420290	-1.205707764865
N	-3.578674048676	0.316821015335	2.016525337363
N	-5.334407507516	-0.977796404367	0.573201288734
C	-4.506065404996	0.346930372886	-2.933477822931
C	-5.018629460801	0.704992707331	-4.174858530040
C	-6.077658129725	1.605739667386	-4.220382870125
C	-6.591834841671	2.109457112519	-3.028206418492
C	-6.028186451330	1.699679270073	-1.822307202923
C	-6.502861044983	2.152313405934	-0.494852459699
C	-7.637689964972	2.937140495479	-0.307630930079
C	-8.009507869184	3.297875626149	0.984551676333
C	-7.238779737544	2.861558301797	2.056959116346
C	-6.119982722900	2.080653220247	1.796049805103
C	-2.664267432605	-1.887295311744	-0.631538722458
C	-1.602912178352	-2.568498835861	-1.214541653033
C	-0.546980054312	-1.831213431188	-1.741109345974
C	-0.581269739027	-0.442855494217	-1.652366429272
C	-1.673246907354	0.172067727825	-1.045245954628
C	-1.820817076768	1.633020384249	-0.887835107231
C	-0.858783201300	2.560316908311	-1.273656800819
C	-1.093902296736	3.915266313923	-1.053062515096
C	-2.287653121191	4.304662682155	-0.456439571803
C	-3.208180073357	3.324436282781	-0.103735050512
H	-3.679422726173	-0.361913134732	-2.858050057573
H	-4.587148756242	0.279134869554	-5.082053955553
H	-7.419581072453	2.818311480868	-3.048064246748
H	-8.237942137623	3.260546291582	-1.157804020271
H	-7.490989710713	3.116142471313	3.087440119042
H	-5.489402263015	1.722879103928	2.610990486856
H	-3.516497392233	-2.433338861767	-0.225787973812
H	-1.616845396665	-3.658340465580	-1.253637433521
H	0.232699658720	0.151708896223	-2.064990957517
H	0.071980328151	2.237406653445	-1.739010011985
H	-2.516921827799	5.353166465215	-0.261240840004
H	-4.154216939664	3.592283287683	0.368789370278

N	-4.996989338709	0.833983958807	-1.791713092225
N	-5.760603945387	1.740893854214	0.554167283117
N	-2.700592916753	-0.552040941387	-0.556123734297
N	-2.978660676911	2.024179403853	-0.311558073635
H	-7.393417819712	-4.005602935867	1.795332724548
H	-2.448092070294	-0.664801619931	5.573063137512
H	-0.345598634346	4.655217816386	-1.345030255353
H	0.298082167674	-2.332378417792	-2.218485387048
H	-6.505560345421	1.917455724275	-5.175726873808
H	-8.898031661401	3.911738490302	1.147546325687
Co	4.231366779531	-0.583975210894	-0.089517927302
C	8.770263566573	0.561092710139	-1.168721603256
C	4.475065215861	-0.722627313786	2.868183067535
C	5.104058722298	-0.533964491642	4.092167502488
C	6.421424688529	-0.088431532123	4.100795533939
C	7.065199050693	0.141963279333	2.888177831319
C	6.374053477133	-0.072636173018	1.698642644130
C	6.971643911396	0.119389008770	0.357409828763
C	8.313393185595	0.423483584363	0.139880354365
C	7.877600985806	0.388579337484	-2.221856041462
C	6.553173125624	0.087652567168	-1.925329258229
H	3.444712746523	-1.076960193842	2.825685914271
H	4.558754383466	-0.735951961450	5.015277335311
H	8.097575894911	0.490762158234	2.876979615837
H	9.004431940854	0.544658648067	0.974079901638
H	8.192405455186	0.483726394012	-3.262309019784
H	5.818937511138	-0.051459554215	-2.721590892358
N	5.091317414974	-0.494230453449	1.703975793453
N	6.116796058082	-0.036264771997	-0.670443169371
C	2.949753387593	0.312283611016	-2.616649031422
C	2.422962596344	0.129859204193	-3.889306522857
C	2.480743241136	-1.138824364254	-4.458962335788
C	3.081470709335	-2.174093081331	-3.747720820032
C	3.600758369670	-1.913790691172	-2.481903125157
C	4.307217759573	-2.909668193024	-1.650604214890
C	4.564744435475	-4.218400543957	-2.045782179502

C	5.263538284867	-5.060942624893	-1.184462585198
C	5.689340636854	-4.570329730742	0.044968814876
C	5.394208593935	-3.253157728785	0.376097834830
C	4.244970452342	2.363371384413	-0.028031459064
C	3.733852539366	3.626102478070	0.246919715355
C	2.473242074980	3.722348142080	0.825812290383
C	1.770081078708	2.555156423925	1.112540909509
C	2.342428022995	1.323363960374	0.807748566539
C	1.675394744955	0.025748795637	1.058355384517
C	0.444435540487	-0.102139973731	1.697907395246
C	-0.096348061626	-1.371105137504	1.880999602472
C	0.604210068365	-2.479179886455	1.415120312162
C	1.835050262192	-2.273268864140	0.800749353177
H	2.918120375266	1.291622782580	-2.137062904280
H	1.971679773753	0.974386316086	-4.412323133070
H	3.144018127729	-3.171348207820	-4.182921344107
H	4.230429373844	-4.584220153901	-3.016529646606
H	6.243233021537	-5.191582989927	0.750302257419
H	5.705196588598	-2.835437826957	1.334357130517
H	5.227149118297	2.247379211212	-0.488634397882
H	4.323822496882	4.511041006640	0.004367136018
H	0.780138400012	2.614464120658	1.565602388876
H	-0.081363693586	0.777945724547	2.064273162439
H	0.214426604029	-3.491771824880	1.527168249706
H	2.424766083063	-3.118338279088	0.439762524517
N	3.513330551123	-0.686053879091	-1.929206473412
N	4.716455172569	-2.448563817425	-0.448243503736
N	3.564931658486	1.244245896998	0.240302452382
N	2.355887512800	-1.054903204510	0.638564934977
H	9.819516675053	0.797140181668	-1.359350407982
H	6.949735375083	0.078666534719	5.041954848716
H	-1.057144295339	-1.496248226434	2.387065049148
H	2.033897991718	4.695829218201	1.053966758634
H	2.067465604510	-1.324381411281	-5.452561902117
H	5.473986260616	-6.091511405298	-1.478399049495

$[Ru(bpy)_3]^{3+/2+}$

ΔE_1^{MM} = -0.00582571 = (5873.26201520 - 2937.73456527 - 2935.54212263)

- (5873.65823693 - 2938.12496128 - 2935.54212263)

ΔE_2^{MM} = -0.00533916 = (5873.27164958 - 2938.12496128 - 2935.16087443)

- (5873.65823693 - 2938.12496128 - 2935.54212263)

E'_1 = -3158.53999257 = (-3158.32426459) - (-1579.15608269) + (-1579.36598496) + (-0.00582571)

E'_2 = -3158.53999267 = (-3158.32426459) - (-1579.15593882) + (-1579.36632774) + (-0.00533916)

G(Def2-SV(P)) = -5494.96263975

Ru	-4.299861001233	0.601235307592	0.077220758899
C	-6.935903982017	-3.206007459038	1.506519246750
C	-2.745433749266	0.982360019117	2.681284657811
C	-2.319662587143	0.669007940581	3.964529824603
C	-2.843472567431	-0.464764817932	4.581408847097
C	-3.793333262614	-1.226962396981	3.908335052835
C	-4.190827724242	-0.846738115277	2.627651428109
C	-5.235907035163	-1.542845681825	1.846219188443
C	-5.943073722540	-2.648628111431	2.308464428011
C	-7.202391827105	-2.640692258509	0.263596456580
C	-6.458029682663	-1.540206641894	-0.140378179000
H	-2.364885749284	1.859766797972	2.154612353892
H	-1.584252076171	1.303983191513	4.460786374382
H	-4.221895474075	-2.107521990631	4.385954186418
H	-5.730308868193	-3.075933752353	3.288028027533
H	-7.974547551436	-3.040520799213	-0.395161146165
H	-6.623989330180	-1.070178255756	-1.111407016650
N	-3.645472746615	0.235754043314	2.026772023685
N	-5.496325253537	-1.011889102219	0.627219395739
C	-4.659661562061	0.477238769393	-2.959879770535
C	-5.232441433955	0.791493032144	-4.184677386918
C	-6.333419151313	1.641847108586	-4.206901438449
C	-6.826876142684	2.143923695107	-3.006304508989
C	-6.208405716473	1.786096719121	-1.811209613322
C	-6.657964628081	2.252491541613	-0.482250806264
C	-7.774518298231	3.059593227646	-0.280447634651
C	-8.121670030202	3.432105857106	1.014936820903
C	-7.346315578699	2.986009575953	2.080978618992

C	-6.247514344146	2.181874714951	1.812748050407
C	-2.649840400771	-1.870104768256	-0.658573797648
C	-1.590385026280	-2.567627324212	-1.221325798230
C	-0.522147033616	-1.846203491737	-1.746470865972
C	-0.545251463916	-0.456968405343	-1.679351352087
C	-1.637683993485	0.183420910315	-1.098629417667
C	-1.761323677500	1.652124587104	-0.974504737117
C	-0.777204551395	2.548364612807	-1.381570254097
C	-0.982928836159	3.914199857358	-1.206269017956
C	-2.172552694076	4.351709420225	-0.633730562116
C	-3.118216259770	3.405642467706	-0.258466692969
H	-3.798782577332	-0.190624855590	-2.898838692389
H	-4.813445386105	0.370474900745	-5.099720856852
H	-7.688195799776	2.811282611248	-3.008994730095
H	-8.378237300434	3.396236346022	-1.122798931209
H	-7.581898750091	3.251429516207	3.112549038108
H	-5.609797994764	1.811306408736	2.617087342807
H	-3.513797269932	-2.395549374455	-0.249252076887
H	-1.612025593979	-3.657746495341	-1.243193535785
H	0.281216135864	0.121685812210	-2.088339957005
H	0.149275757365	2.193370434616	-1.831370634920
H	-2.378387534705	5.411434444894	-0.477013241963
H	-4.066793634439	3.703284295140	0.192660573059
N	-5.134481092919	0.961181998020	-1.804520633129
N	-5.910313945837	1.829629160590	0.564460122503
N	-2.676741715460	-0.530502346310	-0.608333997308
N	-2.914888884080	2.092297549303	-0.419110905893
H	-7.498262872536	-4.073959080077	1.857059842619
H	-2.522641162915	-0.752424166156	5.584738806255
H	-0.215443551552	4.626526081194	-1.516446292637
H	0.324798446461	-2.362208047296	-2.204112026165
H	-6.810011530178	1.914839377832	-5.150795673623
H	-8.995180851367	4.064850358699	1.185998691516
Ru	4.306571056001	-0.591551261419	-0.061732799135
C	8.879599623141	0.658021420175	-1.081279611218
C	4.614465022919	-0.681350753151	2.984531438271

C	5.266031951986	-0.526369015802	4.200555281512
C	6.597254261967	-0.121402040253	4.196339167735
C	7.230995091596	0.109373954916	2.979009758141
C	6.519818345590	-0.067203751476	1.794292749121
C	7.099572247633	0.142286730150	0.449770710540
C	8.427891970730	0.496614980173	0.225171451101
C	7.992695358210	0.458459669307	-2.135036153836
C	6.682113684385	0.106531933881	-1.842936995432
H	3.572177834496	-1.002124873535	2.943468573675
H	4.728550362314	-0.722694520425	5.129451477077
H	8.273703118282	0.425511601349	2.960471686393
H	9.113190513469	0.644607988210	1.059394276964
H	8.302390432365	0.571867788951	-3.174990279801
H	5.950479401495	-0.054961784456	-2.636515293417
N	5.223230411283	-0.457161605518	1.812793849992
N	6.246195741249	-0.042366523062	-0.584921676626
C	2.952510748756	0.181427420093	-2.690237574502
C	2.425115664916	-0.024958134105	-3.957461385948
C	2.522774249210	-1.295084452205	-4.520520526600
C	3.164711464896	-2.304140220211	-3.809347017542
C	3.684495264143	-2.025725978799	-2.545977459358
C	4.423846259449	-3.008356603464	-1.722974188450
C	4.705104545116	-4.308926102377	-2.133450673382
C	5.434406359012	-5.146097009688	-1.293470225453
C	5.869637312935	-4.661778779412	-0.063889230147
C	5.553088908847	-3.355756435318	0.286248731087
C	4.218919954022	2.464331248965	0.064837305447
C	3.665401183249	3.711742997833	0.325257774724
C	2.384232635369	3.773676070531	0.864000851121
C	1.701514903522	2.589084467982	1.126223541676
C	2.312489464860	1.371559809712	0.836624703300
C	1.672986434562	0.057194017086	1.065290354177
C	0.427595492514	-0.100780445348	1.670043535469
C	-0.094724995790	-1.377736213951	1.841900448432
C	0.639530382738	-2.472917574326	1.397181324103
C	1.878075576078	-2.247974838302	0.810714522559

H	2.897686146893	1.159440454537	-2.208031941881
H	1.942059557941	0.800398753474	-4.482726873886
H	3.258282476043	-3.299370043230	-4.243068755223
H	4.364875883343	-4.674064986999	-3.102236663744
H	6.446976855587	-5.280732442584	0.624471464494
H	5.865694876462	-2.935068963882	1.243882317293
H	5.219070084473	2.368105439896	-0.362161130092
H	4.238533624388	4.613328814561	0.104390257052
H	0.696625876669	2.624077685962	1.547639955496
H	-0.124149722897	0.770095793012	2.019027102000
H	0.267044794618	-3.492738778237	1.500933382428
H	2.493968281166	-3.079176665005	0.463630277349
N	3.554146165824	-0.795526280317	-1.998454764526
N	4.844259204595	-2.553871884704	-0.518551747590
N	3.557242421598	1.325969490185	0.307563444717
N	2.385841448633	-1.017201010496	0.659381486328
H	9.918756874409	0.935819498274	-1.270239388903
H	7.143640669901	0.014509633905	5.132103648940
H	-1.067224213299	-1.519416171567	2.320042532022
H	1.913486880663	4.735143773543	1.080169447661
H	2.109424359031	-1.500526755930	-5.510135446269
H	5.661359495864	-6.168417244822	-1.603421288641

5. Cartesian coordinates and energies of MESX geometries by EDEEL with metal-metal distance fixed to r_{DA}(ini) at the UωB97X-D/Def2-SV(P) level

The MESX geometries by EDEEL with metal-metal distance fixed to r_{DA}(ini) and energy components in these geometries are presented below. These energy components are:

$V_{11} = E_{n+m}^{C} - E_{n}^{D} + E_{n+1}^{D}$

$V_{22} = E_{n+m}^{C} - E_{m}^{A} + E_{m+1}^{A}$

In the following, energy values at the UωB97X-D/Def2-SV(P) level are given in Hartree. Note that the Gibbs energy values are before adding the entropy correction of −4.3 kcal/mol.

$[V(H_2O)_6]^{3+/2+}$

V_{11}(Def2-SV(P)) = -2803.297414124407624

= (-2803.085572666943790) - (-1401.540601727514968) + (-1401.752443184979029)

V_{22}(Def2-SV(P)) = -2803.297414110872523

= (-2803.085572666943790) - (-1401.539710866888981) + (-1401.751552310817942)

G(Def2-SV(P)) = -2803.055279092743

E(Def2-SV(P)) = -2803.306609364252836

5 6

V	-2.677379515715	-0.066131263419	-0.060739331285
O	-1.203046867012	-1.420457733580	-0.891102140834
O	-2.559959758203	-1.114777578517	1.718571875567
O	-4.120016492462	1.219721555709	0.652560150214
O	-2.795695980336	0.950840569793	-1.855176434022
O	-1.169630376153	1.154263894100	0.603425397973
O	-4.204719716966	-1.272121356885	-0.748125108700
H	-4.199135804998	-2.236963187553	-0.880085446935
H	-1.398178423866	-1.830961723984	-1.757829676327
H	-3.115943164543	-1.889095959948	1.919091442451
H	-4.477949581528	1.942400605704	0.106430129128
H	-3.181951384555	0.558683546583	-2.658526348414
H	-1.287808208000	2.111897350238	0.736719796424
H	-0.953012326399	-2.152608824053	-0.290826725462
H	-2.272992160610	-0.721351826995	2.562051767730
H	-4.267611524096	1.433426411085	1.590800633358
H	-2.244234360594	1.710876500571	-2.112449670864
H	-0.345067067150	0.873348219596	1.064827835173
H	-5.130826482385	-0.971452295735	-0.715977113516

V	2.672290522087	0.120317552525	0.044332162557
O	4.079112999590	0.137105162720	-1.460542651757
O	2.621991566794	-1.948503815325	0.143631367549
O	1.243004444237	0.181357923588	1.674319013608
O	2.715977457531	2.178075591612	-0.061442126973
O	4.287752533597	0.094961300062	1.316618192772
O	1.122772127249	0.064328530243	-1.279414667665
H	1.168468218007	0.462100312970	-2.167418702804
H	4.186769454002	0.889553683478	-2.069644231803
H	2.192938157283	-2.515754669609	-0.521491984905
H	1.461957616870	0.766288574544	2.427860264722
H	1.939607985335	2.753518817066	-0.182976217813
H	4.330090853069	-0.173949914098	2.251567512623
H	4.428240747619	-0.664124483895	-1.889878544774
H	3.339862288159	-2.459431608441	0.559480802622
H	1.044863138076	-0.697188357060	2.059153815475
H	3.464598093192	2.723551755633	0.239438565599
H	5.193201441932	0.245557509669	0.989619398437
H	0.306962448018	-0.486635437320	-1.222178708070

$[Cr(H_2O)_6]^{3+/2+}$

V_{11}(Def2-SV(P)) = -3004.140574157818264

= (-3003.929446892889246) - (-1501.960164617147029) + (-1502.171291882076048)

V_{22}(Def2-SV(P)) = -3004.140574111951537

= (-3003.929446892889246) - (-1501.960808751714922) + (-1502.171935970776985)

G(Def2-SV(P)) = -3003.899951939371

E(Def2-SV(P)) = -3004.151957943513025

5 8

Cr	-2.656543978072	-0.350718209000	0.013503254378
O	-1.081436897286	0.135502584371	-1.526359759486
O	-2.303955955925	-2.331835672743	-0.145753341731
O	-4.264993251391	-0.665727249449	1.348633104477
O	-2.957937019549	1.642566326357	0.069062460095
O	-1.262868602134	-0.140956301278	1.452567391140
O	-4.142160379138	-0.449918623797	-1.364106552419
H	-4.064674019359	-0.624758068435	-2.319556669841

H	-1.370267971564	0.791171775731	-2.192880752158
H	-2.853206039943	-2.910757240781	-0.705315041318
H	-4.618780157694	0.064670342468	1.888821040988
H	-3.748830803897	2.069511051680	-0.307176339098
H	-1.533351932780	-0.041170049946	2.383999664195
H	-0.748313454633	-0.635469617249	-2.028563901949
H	-1.884554467137	-2.872023532255	0.548586419431
H	-4.338644713339	-1.487209161408	1.868189647448
H	-2.522582769495	2.248421863064	0.695432243944
H	-0.371117196425	-0.571041351287	1.431360610943
H	-5.020267372630	-0.738352468906	-1.052972585127
Cr	2.602936767348	0.410870983190	0.556887281691
O	3.982800500335	1.899090819086	-0.027219569301
O	2.830240154683	-0.666257060606	-1.139191263453
O	1.256340290306	-1.241219191642	1.291018686337
O	2.373861080464	1.449488501302	2.279873811769
O	4.237781824945	-0.470769063383	1.367999807480
O	1.006044910873	1.361163137022	-0.211525215197
H	1.068151238701	2.299015905052	-0.469094626420
H	3.988847390926	2.749544379297	0.448846297173
H	2.503001275666	-0.361391497921	-2.005465087861
H	1.446842118529	-1.601067719871	2.181068875321
H	1.524126355571	1.844762651538	2.547644730508
H	4.351219556517	-1.399554334050	1.638610370051
H	4.203909557675	2.070644900442	-0.960527489836
H	3.625091976021	-1.212261992447	-1.282947100346
H	1.239457594272	-2.010390191636	0.685586261331
H	2.872312506986	1.195085614492	3.078049489434
H	5.111431257029	-0.078609007398	1.183842502485
H	0.282018088758	0.935160295135	-0.735885952951

$[Fe(H_2O)_6]^{3+/2+}$

V_{11}(Def2-SV(P)) = -3442.565443258951746

= (-3442.309747902589152) - (-1721.151270697494056) + (-1721.406966053856877)

V_{22}(Def2-SV(P)) = -3442.565443245694951

= (-3442.309747902589152) - (-1721.151189253019993) + (-1721.406884596125792)

G(Def2-SV(P)) = -3442.325225038344

E(Def2-SV(P)) = -3442.574685019875233

5 10

Fe	-2.653830416987	-0.020018274619	-0.232500451317
O	-1.105976560323	1.460764842602	-0.679352646663
O	-2.377790122898	-0.885539920619	-2.067256101536
O	-4.235372321002	-1.238960380963	0.288374642580
O	-2.940676106311	0.984960512057	1.532929962093
O	-1.263645508654	-1.277979521584	0.558846526416
O	-4.086718026097	1.223780234036	-1.050178511285
H	-3.995076632798	1.888678134646	-1.756149210620
H	-1.339695724308	2.399127237679	-0.530246799255
H	-2.913713090180	-0.662469322624	-2.850186483573
H	-4.596275812269	-1.224412206024	1.193390300850
H	-3.528946945265	1.757567840685	1.615349249273
H	-1.442974936965	-1.868760352303	1.312328361295
H	-0.758754050539	1.406746378954	-1.593356373470
H	-1.938327088649	-1.740511628938	-2.227301329630
H	-4.422890415167	-2.108741789347	-0.109042393195
H	-2.386381644433	0.922121416133	2.331716332796
H	-0.380039176096	-1.506188355959	0.181220880320
H	-5.029062161605	0.990586560340	-0.965446999987
Fe	2.614223786779	-0.270923254579	0.505823757061
O	3.936450460763	0.954916904901	1.509511520457
O	2.882232884907	0.792234455238	-1.223782993356
O	1.261563523415	-1.621808944465	-0.566453401539
O	2.405688722707	-1.417563238270	2.185303451677
O	4.280959854492	-1.397110302262	0.031139868288
O	1.002189711858	0.865158601257	1.016583151299
H	0.979673593894	1.399250315485	1.831139401026
H	4.037914477627	0.935141477255	2.478225107911
H	2.439880850344	1.622734486714	-1.476879823641
H	1.470517052679	-2.577206157378	-0.535722816165
H	1.624758237178	-1.445879160982	2.767062555189
H	4.424377176964	-2.036956397512	-0.688805122723
H	4.230160691722	1.823562984582	1.180891033788

H	3.624175280094	0.634698474837	-1.835462361495
H	1.165300733610	-1.394641501550	-1.513759799545
H	2.982717435445	-2.175102426090	2.390697190871
H	5.141358799581	-1.186652280139	0.437169166746
H	0.278380423376	1.173548447885	0.419622498599

$[Co(H_2O)_6]^{3+/2+}$

V_{11}(Def2-SV(P)) = -3680.603458192495054

= (-3680.299209946947940) - (-1840.146212528100023) + (-1840.450460773646910)

V_{22}(Def2-SV(P)) = -3680.603457908308883

= (-3680.299209946947940) - (-1840.146417066722051) + (-1840.450665028082994)

G(Def2-SV(P)) = -3680.360882698170

E(Def2-SV(P)) = -3680.613526318694312

5 8

Co	-2.638734439171	-0.046474449081	-0.060421456864
O	-1.237974641763	0.136329172067	-1.689844701895
O	-2.625162763552	-2.061406330287	-0.177705983362
O	-3.862945896637	-0.246734258167	1.577888362623
O	-2.671578086937	1.971460439084	0.053106237997
O	-1.108613852260	-0.060744615155	1.227992468783
O	-4.202628481544	-0.057356378766	-1.336845099880
H	-4.278548359003	0.412408677828	-2.187152038406
H	-1.048899730625	1.071380878372	-1.910624861732
H	-3.011091058617	-2.559885784542	-0.921573464191
H	-4.460031882855	0.470769179548	1.857009429218
H	-3.329308784187	2.529303282475	-0.402336659991
H	-1.258102962385	-0.373302412325	2.139254136694
H	-1.463965135761	-0.303361344654	-2.534365441950
H	-1.984212922568	-2.631607155349	0.285748004228
H	-4.317240758933	-1.095673079386	1.727265115496
H	-2.326971849851	2.463066851942	0.821658601806
H	-0.289952233048	0.493512814651	1.206672792355
H	-5.068851500851	-0.449322391434	-1.122773503787
Co	2.626160145029	-0.009274064082	0.061895873188
O	4.004759739909	-1.252654078666	-0.804101470834
O	2.617744793124	-1.261751317606	1.645951295057

O	1.252620547016	1.337072396549	1.024393430650
O	2.640325736871	1.319407226703	-1.466039754703
O	4.200691989524	0.924785596548	0.928503938327
O	1.106708261989	-1.000657680404	-0.775316154675
H	1.183401760567	-1.931737505768	-1.052426894279
H	4.046744143213	-1.413661739219	-1.763938936295
H	1.903932532987	-1.897699114620	1.837068345856
H	1.093067511257	2.140874252386	0.488207588245
H	2.241372482112	1.148642416915	-2.339413780196
H	4.274474483465	1.851751518179	1.219191526035
H	4.236986728521	-2.080215109752	-0.344340151818
H	3.089578614635	-1.054896139859	2.474046527225
H	1.465988555346	1.644978522823	1.928325090203
H	3.368342453316	1.958742384400	-1.580133355725
H	5.097372377115	0.552527219941	0.840558677625
H	0.291441128369	-0.616027078803	-1.183064671996

$[Ru(H_2O)_6]^{3+/2+}$

V_{11}(Def2-SV(P)) = -1105.128766467893001

= (-1104.899865079838037) - (-552.447279350184658) + (-552.676180738239509)

V_{22}(Def2-SV(P)) = -1105.128766469069660

= (-1104.899865079838037) - (-552.446046368063207) + (-552.674947757294831)

G(Def2-SV(P)) = -1104.869946861811

E(Def2-SV(P)) = -1105.138715075217078

5 2

Ru	-2.690204144839	0.047992392758	-0.151650344202
O	-1.170415715980	-0.512177101560	-1.578336040548
O	-2.491016684875	-1.983529446457	0.408186791017
O	-4.205741080222	0.565554262895	1.182666704655
O	-2.893655042208	2.071888557133	-0.710386863659
O	-1.172237473503	0.629315992082	1.121609345156
O	-4.256160178968	-0.373374725957	-1.458072789408
H	-4.251937715554	-1.107941256616	-2.098418087640
H	-1.369897956923	-0.302229512466	-2.513254698871
H	-3.227647176386	-2.577960624745	0.172703601400
H	-4.344445907937	1.525091055704	1.297254868805

H	-3.266786221106	2.227894995760	-1.598570643122
H	-1.384942984190	1.161095438100	1.909621531367
H	-1.026245085368	-1.480339037226	-1.531079516077
H	-2.259747071314	-2.177178713786	1.335561902992
H	-4.265743141216	0.134893375273	2.055046541176
H	-2.093465419750	2.623672451636	-0.629362710696
H	-0.387105846642	0.062218793805	1.320467495022
H	-5.156849957885	-0.278785993013	-1.094271561233
Ru	2.673165374671	0.004911489959	0.164853736661
O	4.190512937764	0.792607553793	-1.042150060390
O	2.781072694513	-1.623694477641	-1.143878295867
O	1.154841780414	-0.782294365578	1.510105891505
O	2.609696330353	1.626971726845	1.474291417823
O	4.250452077511	-0.723622152668	1.310731816989
O	1.144786009757	0.901392915159	-0.926186950150
H	1.399514788692	1.515115908770	-1.638786169700
H	4.307496294705	1.759005994178	-1.095677160653
H	1.997789784430	-2.161543919869	-1.358169759781
H	1.405758294727	-0.618007258451	2.442923684612
H	1.957564764153	2.331244382158	1.299983973792
H	4.226952993268	-1.597049698679	1.742964918761
H	4.249245682890	0.433936899688	-1.948034684739
H	3.547875725443	-2.223773098493	-1.085112780749
H	0.990356602157	-1.745096561987	1.441131613822
H	3.439596314146	2.042493484339	1.773848094947
H	5.144123087301	-0.574422865441	0.948071103704
H	0.366691234369	0.378908355398	-1.234006947455

$[Co(NH_3)_6]^{3+/2+}$

V_{11}(Def2-SV(P)) = -3442.696463510656031

= (-3442.510373964913015) - (-1721.257720332878080) + (-1721.443809878620868)

V_{22}(Def2-SV(P)) = -3442.696463498868980

= (-3442.510373964913015) - (-1721.257715265390971) + (-1721.443804799346935)

G(Def2-SV(P)) = -3442.270547291802

E(Def2-SV(P)) = -3442.699017642926719

5 4

Co	3.568130782296	0.133968334103	0.002149204446
N	3.605717757763	1.698144020153	-1.379007599946
H	3.287094053599	2.591784767282	-0.991753526222
H	3.025196364701	1.527791622129	-2.206326820565
H	4.551288584935	1.874210716462	-1.734104447252
N	2.302915870170	1.199883255391	1.272284763749
H	1.602587202165	0.588211192563	1.703336700223
H	1.771360889945	1.940205303985	0.803923859927
H	2.792523802381	1.653496107761	2.049995584495
N	3.640855988030	-1.432668895621	1.385239836425
H	2.984407384384	-2.193195178039	1.182830132080
H	3.440652046044	-1.139319224901	2.346902573283
H	4.567329916913	-1.870201663544	1.414110339601
N	1.879687480817	-0.650094621246	-0.942299441523
H	1.205411019092	0.071438075104	-1.214750469561
H	1.359115729211	-1.310113519324	-0.356673900723
H	2.104952671304	-1.160287247698	-1.801956193516
N	4.765514072565	-1.024959024406	-1.261770347472
H	4.743052718866	-0.712878036844	-2.237752053879
H	4.477533110420	-2.008789737584	-1.275850114051
H	5.755201664525	-1.032481774304	-0.996271424474
N	5.233226152136	0.982536503343	0.937948575753
H	5.138083969288	1.992764362189	1.082403084302
H	6.103271767448	0.862935232246	0.409808510837
H	5.416610604330	0.582456830146	1.863424830656
Co	-3.567658897670	-0.129317564957	-0.002855939139
N	-3.199088147231	1.011955428019	-1.714506763117
H	-4.069882293625	1.299836699605	-2.171974159531
H	-2.659188872753	0.519770641285	-2.433101823929
H	-2.684845822575	1.878265136897	-1.525569406923
N	-4.835572671944	1.387565142477	0.675981506635
H	-5.827554153936	1.201862797938	0.498708127308
H	-4.643419808419	2.293453213331	0.236674048468
H	-4.761966159404	1.543827093712	1.686301206249
N	-4.045091483681	-1.233953485780	1.700817049566
H	-3.750011923023	-2.214100680306	1.649299564840

H	-5.056133519000	-1.254847929692	1.869512076669
H	-3.629916407877	-0.859440052027	2.559564811738
N	-5.113608404878	-1.070881871400	-1.050315196864
H	-5.945255497841	-0.486635189752	-1.181946209420
H	-5.446120480790	-1.925545012182	-0.592899722327
H	-4.825718355762	-1.353540622448	-1.992611453401
N	-2.263419412393	-1.642753129147	-0.614095741415
H	-1.627248251031	-1.347611044825	-1.361161227513
H	-2.742072120859	-2.475756330345	-0.971059374142
H	-1.661495911907	-1.971475976619	0.147306532688
N	-1.969384105833	0.776001833208	0.989311698706
H	-2.225113285383	1.645511265370	1.467630000343
H	-1.210974891254	1.026238918165	0.347879201835
H	-1.542127135270	0.177311698125	1.702503468045

$[Ru(NH_3)_6]^{3+/2+}$

V_{11}(Def2-SV(P)) = -867.287538130126450

= (-867.097813447911449) - (-433.550944332322160) + (-433.740669014537161)

V_{22}(Def2-SV(P)) = -867.287538117054282

= (-867.097813447911449) - (-433.551143413336604) + (-433.740868082479381)

G(Def2-SV(P)) = -866.860900293242

E(Def2-SV(P)) = -867.290207556746736

5 2

Ru	3.510336687685	0.088039151964	-0.128177149946
N	4.480273475469	-0.107691275111	-2.037037228113
H	3.894719521159	0.205856744170	-2.817853226767
H	4.737549871584	-1.074632066708	-2.259591300990
H	5.351308814214	0.428344950415	-2.108987423222
N	2.647005080701	1.958533699873	-0.740100727121
H	1.639345307280	1.908996670092	-0.918465479357
H	3.058293925080	2.327854271715	-1.603762225849
H	2.765815801225	2.699879527912	-0.041806683807
N	2.511513584722	0.263012408936	1.767313060274
H	1.957296383203	-0.567547713163	2.001788983412
H	1.852412056034	1.047288907642	1.802823503205
H	3.145012808496	0.396115374744	2.562359109786

N	1.845655651458	-0.995993764863	-0.952103780183
H	1.211136024683	-0.407449405291	-1.500461305040
H	1.257442189534	-1.442629756494	-0.241277149392
H	2.131925735085	-1.749221566102	-1.585536038098
N	4.409729517949	-1.758844346602	0.513127454734
H	5.299357474144	-1.956278412433	0.043158608005
H	3.821239394518	-2.580274312809	0.340106263764
H	4.617498422597	-1.777589750235	1.516768823019
N	5.192404890155	1.167615878849	0.658069524187
H	5.467250362503	1.959564428930	0.066879668982
H	6.035256166399	0.592955430982	0.757719381914
H	5.019895627706	1.572843150590	1.583525738457
Ru	-3.519245869589	-0.088959642997	0.130914770142
N	-4.001214121571	0.315445878330	-1.929210896381
H	-4.975841559968	0.605988435705	-2.057371691184
H	-3.870455999528	-0.496644152335	-2.540996374700
H	-3.436740819000	1.063446445809	-2.344273757284
N	-4.653392778652	1.635970135404	0.732205753445
H	-5.668238015330	1.502614719448	0.683137569942
H	-4.460283278587	2.454008393812	0.144158045574
H	-4.458131269419	1.936536808838	1.692709579362
N	-3.085319840923	-0.492189068015	2.198340110797
H	-2.716513813600	-1.433889484047	2.365392109991
H	-3.914466469837	-0.415682122248	2.795958444538
H	-2.393382454882	0.147878116413	2.600587013914
N	-5.272230614388	-1.325749078886	0.261940448507
H	-6.047299824453	-0.890005373513	0.771433905009
H	-5.093212758886	-2.209287243747	0.751121020108
H	-5.653707371000	-1.588743072193	-0.652236075579
N	-2.359400217725	-1.786505579700	-0.506706536182
H	-1.726197724274	-1.555121985438	-1.278703469593
H	-2.929546305332	-2.569080807032	-0.843107802736
H	-1.760661083310	-2.177059064686	0.227345558072
N	-1.739313358490	1.121858785216	0.026738099764
H	-1.854150816008	2.051352644312	0.443159488210
H	-1.401879931823	1.286638402503	-0.926549972039

H	-0.953235150373	0.693793349705	0.525077400206

$[Ru(NH_3)_5py]^{3+/2+}$

V_{11}(Def2-SV(P)) = -1250.346858971574875

= (-1250.151098990277887) - (-625.073141064543393) + (-625.268901045840380)

V_{22}(Def2-SV(P)) = -1250.346858971454822

= (-1250.151098990277887) - (-625.073626527178817) + (-625.269386508355865)

G(Def2-SV(P)) = -1249.821063282066

E(Def2-SV(P)) = -1250.349972976428035

5 2

Ru	-4.529621865167	-0.082186080665	0.018152907471
N	-4.098085751651	-1.469456973721	-1.566566782099
H	-4.244878140394	-2.448239170638	-1.299751656546
H	-3.128662590117	-1.430738190955	-1.896229676453
H	-4.678482117254	-1.326672430763	-2.399394950994
N	-6.490656007607	-0.952630748944	0.143167134455
H	-6.889804861540	-0.942999269348	1.087327956871
H	-6.506957602563	-1.934479844016	-0.151779491904
H	-7.176283295717	-0.482159394859	-0.456490691831
N	-3.876702610490	-1.517986276972	1.477222561385
H	-2.955427824518	-1.312160051067	1.875812671913
H	-3.799892856214	-2.469771846823	1.105110019568
H	-4.513113199667	-1.590566948186	2.277733487528
N	-5.041105725126	1.285412984230	1.591907087027
H	-4.514857678383	2.164615545602	1.544715000851
H	-4.897076606592	0.917990779591	2.538059348273
H	-6.028511338396	1.561121797080	1.561037254691
N	-5.255633410777	1.356820887497	-1.401345889711
H	-5.432456258214	0.983854264632	-2.339654642841
H	-4.609381522201	2.143351677619	-1.525811611142
H	-6.140276430974	1.786847193096	-1.110992281274
C	-0.104307728550	2.007475564363	-0.308142352896
C	-0.657674956454	1.710472849475	0.934735017778
C	-1.903163995793	1.100493579200	0.986313127414
N	-2.603479650458	0.777542074427	-0.117407853109
C	-2.056033757618	1.055452892917	-1.317090138273

C	-0.817445527555	1.664795676058	-1.454626317670
H	0.866023537996	2.503014259825	-0.381925177943
H	-0.139771190029	1.951643359169	1.864705991350
H	-2.358038089249	0.872688970984	1.952082006407
H	-2.634012299294	0.787543390791	-2.204246148949
H	-0.427722552466	1.868046216883	-2.453516036827
Ru	4.530079763643	0.086050139963	0.031643702242
N	6.551617270158	0.823858362536	0.184549754307
H	7.225071593909	0.283376752262	-0.367825079427
H	6.907960507785	0.813046864130	1.145717934754
H	6.656306137448	1.791149687642	-0.138599255796
N	5.117517157410	-1.238003426979	-1.560937093010
H	6.052479898521	-1.635445334757	-1.424820174216
H	5.147348859764	-0.792431307780	-2.483374179249
H	4.495936523055	-2.047881750446	-1.655955508741
N	5.013674619920	-1.470566294587	1.437174840083
H	5.939305176382	-1.876686648166	1.265225199153
H	4.360309954766	-2.259392093579	1.386834150198
H	5.028985716024	-1.178189135564	2.419374405946
N	3.994056977361	1.431434226826	1.620535055644
H	4.506427574511	2.317783065204	1.572497167318
H	4.174645120954	1.071065307893	2.562581128209
H	3.002631161281	1.690936395092	1.606847041766
N	4.105972368008	1.638992707552	-1.398620385786
H	4.808844206848	1.702248673724	-2.142334345261
H	4.064519832742	2.573501616195	-0.979717856284
H	3.209463821290	1.522724549592	-1.880691589308
C	0.036456670819	-1.857451321866	-0.205726535381
C	0.714478546418	-1.505069322600	-1.369984480221
C	1.966659817224	-0.915184109837	-1.264635694845
N	2.559179325221	-0.665905622694	-0.081860924193
C	1.884900055436	-0.982223056738	1.040057052335
C	0.630895459866	-1.576607972949	1.022049401434
H	-0.937117310758	-2.349715173184	-0.254228735931
H	0.289534992884	-1.690878809125	-2.358055741508
H	2.522193600306	-0.646262294236	-2.165544366858

H	2.373662789230	-0.761610466803	1.991045550568
H	0.139301252817	-1.821161990048	1.965458593257

$[Co(en)_3]^{3+/2+}$

V_{11}(Def2-SV(P)) = -3906.715313136320219

= (-3906.529880136416068) - (-1953.266476957097893) + (-1953.451909957002044)

V_{22}(Def2-SV(P)) = -3906.715314421212952

= (-3906.529880136416068) - (-1953.266262240199012) + (-1953.451696524995896)

G(Def2-SV(P)) = -3906.061098153951

E(Def2-SV(P)) = -3906.717253507189980

5 4

Co	-3.873543706532	0.067386073654	-0.012046321930
N	-1.841840968073	0.443739017762	0.274256422314
N	-4.077773824360	1.612370762429	1.382563836433
N	-3.938018339659	1.359805623230	-1.657340201658
N	-3.476666093987	-1.363208483148	-1.484583364256
N	-3.966479144451	-1.380452126094	1.490999619343
N	-5.938498643143	-0.257056170790	-0.087585774465
C	-1.686549489430	1.738227415963	0.968762646149
C	-2.768210576201	1.848675938218	2.024549834912
C	-4.054143188524	0.539380101462	-2.881757332212
C	-3.142925278643	-0.663265745331	-2.743523013749
C	-5.286583597601	-2.043036606212	1.422770190942
C	-6.339585999004	-0.993921199723	1.129409119421
H	-4.712057873248	2.028644968589	-1.615195970546
H	-3.096213786021	1.940080898897	-1.714696968106
H	-4.281126579760	-1.977525489330	-1.638029503995
H	-2.713352573931	-1.994442529161	-1.225302026859
H	-4.378193442905	2.461242425563	0.894524290624
H	-4.794158812192	1.457687345336	2.096633367852
H	-1.300990651606	0.440772986818	-0.592547808472
H	-1.433861814271	-0.303050747944	0.844195409030
H	-3.840363424405	-0.960043027860	2.415992210063
H	-3.226589129200	-2.084120146950	1.420284154509
H	-6.205576670566	-0.796608201783	-0.915637685442
H	-6.473783510088	0.612710933766	-0.161908853368

H	-7.331854898527	-1.456638158346	1.009999822022
H	-6.410243078163	-0.276339650177	1.961873793899
H	-5.515271824954	-2.578444310042	2.357659486604
H	-5.253474169724	-2.789091554277	0.613504628257
H	-1.790814526146	2.545309669855	0.226789317469
H	-0.687552387634	1.837055606654	1.421115065296
H	-2.621101474473	1.081775912293	2.801468327635
H	-2.733486272090	2.831716134147	2.519924713693
H	-2.090035331152	-0.342988895227	-2.693817419009
H	-3.241284988632	-1.331984956917	-3.612926289577
H	-5.102159976758	0.216107112334	-2.981760524561
H	-3.800665900015	1.122060421719	-3.781370589917
Co	3.891531311702	-0.069908494471	0.010985466454
N	5.333591151497	-1.329985357322	0.852866225815
N	5.486652672181	0.476436760106	-1.228123525268
N	3.225805631187	-1.583142113830	-1.264183927007
N	2.400874372643	-0.826291680476	1.265324252198
N	4.295866686149	1.505096357056	1.321620847738
N	2.609798624038	1.330766138276	-0.860175959587
C	6.502471634298	-1.390632112126	-0.049899856701
C	6.745245799093	-0.010262479941	-0.626043102639
C	1.902227377568	-2.034144166064	-0.786026792669
C	1.941309440812	-2.124227313899	0.726554223368
C	3.235029683635	2.523229842754	1.168146652782
C	2.912607416457	2.667859003530	-0.306151363960
H	3.158416444454	-1.282473464173	-2.240121864656
H	3.873462180638	-2.376220053074	-1.277477631728
H	1.607035476921	-0.185861699770	1.341930037110
H	2.732155550825	-0.943010940264	2.226620847374
H	5.360238256466	0.044638441553	-2.148407106513
H	5.558632172005	1.478929851447	-1.418744337982
H	5.002995066487	-2.276496819270	1.057587324759
H	5.622605032558	-0.953917422995	1.760790479895
H	5.206912197866	1.928016994655	1.122945179014
H	4.351575134167	1.209309951876	2.299879993618
H	1.630697104036	1.100212137780	-0.678672646859

H	2.685572593256	1.348136382986	-1.881089056859
H	2.072383404546	3.363784131734	-0.455946197177
H	3.778719603881	3.078946565853	-0.847612668789
H	3.542810183830	3.490350738373	1.596100835018
H	2.349747747286	2.184714057786	1.728514775209
H	6.278635947558	-2.105119203386	-0.857421859524
H	7.397571390178	-1.758090246197	0.476087443022
H	7.043403753241	0.692143350952	0.168354986316
H	7.561497477456	-0.035037869289	-1.365372331513
H	2.653542866678	-2.901905136527	1.044068358686
H	0.953457060037	-2.398819095031	1.128737698430
H	1.151631708608	-1.296377744945	-1.108538974611
H	1.622759498802	-3.003454840985	-1.228041482225

$[Ru(en)_3]^{3+/2+}$

V_{11}(Def2-SV(P)) = -1331.298731632575709

= (-1331.107100323488112) - (-665.554889362532549) + (-665.746520671620033)

V_{22}(Def2-SV(P)) = -1331.298731701906718

= (-1331.107100323488112) - (-665.554947127917103) + (-665.746578506335709)

G(Def2-SV(P)) = -1330.643924392442

E(Def2-SV(P)) = -1331.301071943684065

5 2

Ru	-3.907936323779	0.039055352007	-0.026228322528
N	-1.860142158307	0.583442169090	0.313743621962
N	-4.200558232199	1.547491043933	1.478773080855
N	-3.971712660555	1.411176349058	-1.680970957701
N	-3.457964798867	-1.328431530664	-1.620968670257
N	-3.960886523300	-1.469943807178	1.505142609743
N	-5.985359137104	-0.496232726451	-0.148303313666
C	-1.808898741098	1.852232122708	1.079282823054
C	-2.900965107123	1.842951455376	2.130252510241
C	-4.047884812242	0.634820713133	-2.942713037002
C	-3.117693783041	-0.556949023666	-2.841835146093
C	-5.220142633442	-2.243305297415	1.373708038716
C	-6.352621330445	-1.289997016282	1.050097163598
H	-4.767270922546	2.054311902947	-1.632706126130

H	-3.150592082813	2.022860045988	-1.710865733456
H	-4.240227483823	-1.956147047769	-1.827373243729
H	-2.682203650000	-1.954760964596	-1.387991773857
H	-4.562823619534	2.398939204313	1.038512913685
H	-4.894458783150	1.317613639409	2.194671392885
H	-1.299580365046	0.677256672967	-0.535633218321
H	-1.399297058555	-0.157939183860	0.848325758473
H	-3.907604501119	-1.067536991478	2.445534728473
H	-3.165511141507	-2.113401588159	1.456088600167
H	-6.198568647739	-1.040867051665	-0.989198708992
H	-6.596145135458	0.323332002812	-0.212787350263
H	-7.292063600551	-1.841267480935	0.887017994457
H	-6.518600151821	-0.591529598909	1.884705711651
H	-5.435768309845	-2.807689856273	2.294584667764
H	-5.087459070976	-2.971595165664	0.558646683310
H	-1.965614243017	2.682354940241	0.372973006182
H	-0.820410639226	1.995050719218	1.544184351397
H	-2.706561672956	1.057051514058	2.877069244391
H	-2.934231011644	2.806013761534	2.663508414195
H	-2.071820014335	-0.222943444483	-2.755412094050
H	-3.191280811528	-1.187454930756	-3.741617141893
H	-5.088356602475	0.298560507358	-3.073070047819
H	-3.787806162939	1.261830536879	-3.810001644539
Ru	3.912579234808	-0.035757615661	0.023214435901
N	5.390070825737	-1.386854520707	0.817203413325
N	5.510094756558	0.342007835112	-1.367994876833
N	3.058836334596	-1.658210598028	-1.096671812184
N	2.376173845327	-0.627266630555	1.402490323401
N	4.548373899219	1.645319384544	1.200200599222
N	2.613640708450	1.463345812234	-0.797041105003
C	6.476552678895	-1.557946614185	-0.177689408203
C	6.771222940000	-0.221511028982	-0.826351021516
C	1.745752205488	-2.015201435116	-0.504859157822
C	1.850034931201	-1.957016796409	1.005899460673
C	3.535446401219	2.721935859147	1.078723496174
C	3.082342845821	2.803433279740	-0.364625156865

H	2.929093844325	-1.435374408104	-2.087922224842
H	3.665875737560	-2.482943204841	-1.099464638001
H	1.605409778780	0.045347427328	1.440421594040
H	2.719853560041	-0.673864578549	2.366255116322
H	5.300985195870	-0.107255771141	-2.265151277080
H	5.656295230374	1.326300533301	-1.607088306606
H	5.039857799941	-2.307556351583	1.095780949439
H	5.780195884628	-0.992044541539	1.678842194148
H	5.461087013071	2.006713146097	0.908253419258
H	4.671969755932	1.407905512465	2.188917121715
H	1.641654225874	1.338952181974	-0.504122868171
H	2.577592996905	1.432092498262	-1.820037528616
H	2.288419122930	3.556965564258	-0.484302453639
H	3.921296493304	3.096581543037	-1.014840514013
H	3.942428786060	3.687819096335	1.416601151025
H	2.689169585980	2.466990863444	1.735662028302
H	6.135967921693	-2.282021596951	-0.934130156100
H	7.382740740603	-1.970443220167	0.292730180554
H	7.171994309582	0.485863794834	-0.083561053175
H	7.526893227971	-0.332959015612	-1.619844216282
H	2.547856111791	-2.724069445839	1.375614410257
H	0.871307470731	-2.149747773373	1.472483043563
H	1.003524825805	-1.288118279535	-0.866968944863
H	1.422487655030	-3.014843598803	-0.835397619634

$[Fe(bpy)_3]^{3+/2+}$

V_{11}(Def2-SV(P)) = -5495.854571864499121

= (-5495.652002809723854) - (-2747.819885154470740) + (-2748.022454209246007)

V_{22}(Def2-SV(P)) = -5495.854571868372659

= (-5495.652002809723854) - (-2747.819957364291895) + (-2748.022526422941155)

G(Def2-SV(P)) = -5494.952807644207

E(Def2-SV(P)) = -5495.858605522569633

5 2

Fe	-4.267531369281	0.669611604322	0.073403486406
C	-6.965057238447	-2.969282559916	1.509760801063
C	-2.719129467205	1.171529870654	2.562391484963

C	-2.303862021210	0.912071976838	3.862019593760
C	-2.855595323214	-0.176304425566	4.532649525866
C	-3.815499181692	-0.952575933470	3.889801328947
C	-4.194065340020	-0.623398515277	2.590499023302
C	-5.240593032018	-1.330789333924	1.824817649219
C	-5.985684378792	-2.397363085268	2.318130853602
C	-7.175990860231	-2.455396714225	0.234446875049
C	-6.392822247885	-1.390119583768	-0.192834371229
H	-2.313945256452	2.016379533209	2.002838473252
H	-1.558637615280	1.556924488264	4.330653851668
H	-4.266455448575	-1.801282276048	4.403779144616
H	-5.812204832408	-2.781611129338	3.323187462613
H	-7.934685048572	-2.868052872943	-0.432229089667
H	-6.522825060139	-0.965884135678	-1.189563126627
N	-3.633257703961	0.418239731351	1.940788518843
N	-5.445990688633	-0.847837379168	0.580061793675
C	-4.566566393412	0.411325090052	-2.891792236729
C	-5.110676214437	0.717780550990	-4.132381627990
C	-6.179326329997	1.606186701509	-4.190611570621
C	-6.670949885720	2.149800940865	-3.007326496550
C	-6.078459133357	1.793097561684	-1.799204813402
C	-6.524050765636	2.292521677458	-0.482922087184
C	-7.637140236648	3.107239128581	-0.295313069781
C	-7.972384910977	3.510799681286	0.994017017785
C	-7.187081973587	3.085833000145	2.060641155795
C	-6.092722660988	2.271607608042	1.799096004163
C	-2.752867706168	-1.816336162854	-0.584340863068
C	-1.711101247333	-2.541242410146	-1.148768225871
C	-0.642926641642	-1.847583700955	-1.708789162118
C	-0.641874050721	-0.456960796232	-1.659119493203
C	-1.714798871254	0.204954161911	-1.067649750217
C	-1.825668274113	1.674380749459	-0.959085156669
C	-0.846931486662	2.566031941949	-1.387725372705
C	-1.056512704019	3.932950468170	-1.224081047793
C	-2.241665371580	4.370617347058	-0.642465244542
C	-3.177882496363	3.423563335597	-0.244985273516

H	-3.732556871753	-0.286780948123	-2.812619393289
H	-4.693831064439	0.261031704504	-5.031209970317
H	-7.507189432425	2.848137487874	-3.033395595506
H	-8.246728134211	3.423074913398	-1.141751808545
H	-7.410690634716	3.374513703067	3.088759760760
H	-5.454865279469	1.923801717189	2.612397805464
H	-3.617066989762	-2.328006145917	-0.159162889451
H	-1.752703282395	-3.631359350128	-1.152839892234
H	0.182689321586	0.104176637179	-2.095832802755
H	0.073969837250	2.208858355328	-1.847209079554
H	-2.450637145042	5.431123573139	-0.494337782735
H	-4.118029801904	3.727722657245	0.218332788198
N	-5.035301393579	0.936302425441	-1.753799268447
N	-5.766388152678	1.888009674369	0.559376882683
N	-2.761200033220	-0.478003288185	-0.557447392628
N	-2.971843275008	2.110729933721	-0.394077732608
H	-7.559342514779	-3.807652175805	1.879331473381
H	-2.549204424717	-0.417576271467	5.552741047711
H	-0.296582971409	4.646169541633	-1.550667417454
H	0.184307563221	-2.383639569482	-2.179231887298
H	-6.631499275169	1.876420661138	-5.147304380430
H	-8.842657203701	4.149566938849	1.159511781639
Fe	4.271389037086	-0.670403518253	-0.073830936095
C	8.647633848758	0.890075734554	-1.099733535868
C	4.491023270200	-0.842128760442	2.903725431369
C	5.103918375941	-0.640070692686	4.133715216318
C	6.387015940401	-0.103658707976	4.158886720856
C	7.014524447706	0.203868273323	2.955047652405
C	6.339944829313	-0.028836370383	1.759603554468
C	6.909945422625	0.239262993076	0.423730843044
C	8.209497032658	0.688770932821	0.206129928896
C	7.775673035013	0.632193379565	-2.152468453429
C	6.492624766442	0.187445877410	-1.860567949181
H	3.489246555238	-1.269734085129	2.850012144410
H	4.573259225215	-0.903282885922	5.049910787671
H	8.020714715736	0.622574312513	2.955516980891

H	8.882088677915	0.877631096863	1.042616860385
H	8.074561902292	0.771084991157	-3.192552873659
H	5.781954033920	-0.017714428018	-2.661918542293
N	5.090716834763	-0.542388391302	1.746013773949
N	6.067919258580	0.002406783666	-0.605358918835
C	2.938120477204	0.134862861393	-2.605811457127
C	2.451407258770	-0.060471992129	-3.891724766745
C	2.637348711024	-1.302559242686	-4.492641636350
C	3.317690239777	-2.296851956847	-3.795636698447
C	3.788085741446	-2.024869997514	-2.513072449592
C	4.568884233951	-2.974804306888	-1.694105973079
C	4.939524702219	-4.249033302339	-2.112902999573
C	5.697503331703	-5.047463573404	-1.259998796887
C	6.068827981172	-4.548079614313	-0.015972223161
C	5.659883094077	-3.267823609522	0.337210684090
C	4.074419334023	2.300869443635	0.075732891046
C	3.474162374635	3.509434815519	0.408162783754
C	2.214831071595	3.488028241159	0.997829623940
C	1.599043360198	2.261536574640	1.233505441971
C	2.256726950664	1.090767419768	0.867476744285
C	1.701652815871	-0.266070308801	1.054939517385
C	0.478599268568	-0.534466674021	1.664960715523
C	0.053449434966	-1.853388258603	1.789400204715
C	0.856579325976	-2.870689680630	1.283958236058
C	2.069272110852	-2.531644806089	0.697717127167
H	2.819563692067	1.096142243136	-2.103339272618
H	1.937129673097	0.753839071632	-4.404847923469
H	3.483191297733	-3.271069469458	-4.255157893901
H	4.646343339142	-4.622656034386	-3.094005857637
H	6.666088955728	-5.135672307004	0.682870408876
H	5.923744919821	-2.845172714686	1.308065825262
H	5.060368162125	2.277988589932	-0.391873911459
H	3.996329970835	4.445395508470	0.204200792057
H	0.615260937079	2.228165794433	1.700211465606
H	-0.134765714113	0.274653222549	2.058411308041
H	0.562317983054	-3.919299615117	1.347176991429

H	2.733825586687	-3.306481723636	0.313702628801
N	3.581093431948	-0.824696682262	-1.931681363507
N	4.923381462332	-2.505394344514	-0.477900009576
N	3.478966547905	1.123646134566	0.294320504111
N	2.487400053848	-1.263971884807	0.598679469543
H	9.664001374837	1.242456617212	-1.288457587107
H	6.901154141643	0.074211328279	5.105866770725
H	-0.897164014793	-2.082823354129	2.276025077981
H	1.710072050113	4.416230564034	1.274484382890
H	2.263772776914	-1.496884775988	-5.500257348789
H	5.996206226260	-6.050779590827	-1.571383641858

$[Co(bpy)_3]^{3+/2+}$

V_{11}(Def2-SV(P)) = -5733.941444173276977

= (-5733.763712038402446) - (-2866.875828421020742) + (-2867.053560555894819)

V_{22}(Def2-SV(P)) = -5733.941444150037569

= (-5733.763712038402446) - (-2866.875564021395348) + (-2867.053296133030926)

G(Def2-SV(P)) = -5733.042023102784

E(Def2-SV(P)) = -5733.945827427990480

5 2

Co	-4.224691311278	0.561805332032	0.091775640689
C	-6.865592281047	-3.114049418270	1.469581365109
C	-2.725467090719	1.089622941486	2.684467254420
C	-2.299845597749	0.784851227225	3.972448177941
C	-2.815553408044	-0.351521525146	4.590209742855
C	-3.752385452373	-1.127895783180	3.912904973850
C	-4.141055466068	-0.748618381409	2.629505566531
C	-5.165547188405	-1.456396919747	1.828812998656
C	-5.910160347644	-2.533425722919	2.300138022268
C	-7.054533715658	-2.599552655386	0.191556213454
C	-6.275095850425	-1.523209005013	-0.214250288510
H	-2.349061029430	1.971765348076	2.161490391477
H	-1.575978562096	1.430147375650	4.473220449542
H	-4.170555161826	-2.015660853579	4.387121169700
H	-5.756609304961	-2.917888232685	3.308314059367
H	-7.793260530320	-3.018568700155	-0.493222541811

H	-6.387118080108	-1.095073177441	-1.211157348735
N	-3.612992819531	0.335709335584	2.032495454569
N	-5.353752152521	-0.972515119920	0.581793636427
C	-4.479986855107	0.299273616929	-2.944448746408
C	-4.975799439411	0.646976405693	-4.195714812163
C	-6.024174986339	1.558773651677	-4.263859102741
C	-6.545905450265	2.082425835499	-3.083626853688
C	-5.999438084088	1.680913765142	-1.866967942256
C	-6.486415285614	2.150963686093	-0.550063517936
C	-7.618733950132	2.944557723200	-0.385668511953
C	-8.007028856644	3.319258699759	0.897567844777
C	-7.254114293343	2.888212379491	1.984612291234
C	-6.136124932789	2.099062573255	1.746782185021
C	-2.666922619309	-1.888141338387	-0.562560238069
C	-1.601309115217	-2.581181818802	-1.123282538516
C	-0.546837567311	-1.855301137294	-1.667495456601
C	-0.582794953399	-0.465252513768	-1.608897807173
C	-1.677687187842	0.162804129608	-1.022076177770
C	-1.826298752380	1.626897666482	-0.898451459382
C	-0.871459184841	2.548159326785	-1.315628514105
C	-1.109530940369	3.906817261948	-1.123018598856
C	-2.297382131765	4.305777589254	-0.520524171505
C	-3.211388308942	3.330787544300	-0.138099013889
H	-3.662179377254	-0.417813996434	-2.851356215399
H	-4.540016057707	0.205382847101	-5.093291724229
H	-7.366188887560	2.799215901173	-3.121156414811
H	-8.204874516328	3.263403371111	-1.247301587199
H	-7.519738470219	3.153079906008	3.009124675529
H	-5.518609843145	1.746237207822	2.573567345625
H	-3.520066331168	-2.425795100886	-0.147790371505
H	-1.614260869242	-3.671872679735	-1.136417528372
H	0.229202632527	0.121894247454	-2.034768342142
H	0.054120216078	2.219619162929	-1.786597541662
H	-2.527041279807	5.357776696712	-0.345138915100
H	-4.153576012825	3.605345195488	0.338462156590
N	-4.977395054821	0.806062638005	-1.814334562069

N	-5.760877771419	1.745211832322	0.513283143343
N	-2.707211088617	-0.551741557186	-0.522678879643
N	-2.980214159491	2.027407461644	-0.322382381329
H	-7.458495654331	-3.959605483148	1.824740117982
H	-2.497931285730	-0.631888079050	5.596886574507
H	-0.367871611410	4.642626571551	-1.441336175370
H	0.297067416587	-2.365863947169	-2.137008670299
H	-6.438570127532	1.863234462124	-5.227479101374
H	-8.894270366998	3.939528134072	1.042186104505
Co	4.231366779531	-0.583975210894	-0.089517927302
C	8.685650750761	0.705885716406	-1.150252854567
C	4.519133101996	-0.769645266450	2.947230638658
C	5.154674273675	-0.545319779357	4.162987245499
C	6.448010565636	-0.033366282508	4.150774284288
C	7.063356538995	0.225805994915	2.928738535970
C	6.363473722755	-0.029720488938	1.751932686938
C	6.924059644843	0.185776177220	0.398486752937
C	8.248207968430	0.546210308205	0.161653811612
C	7.788278802114	0.497752818409	-2.192274559438
C	6.482176803902	0.141188533635	-1.882032057440
H	3.506610811909	-1.176290655052	2.918402929829
H	4.635053637195	-0.770611766191	5.095617559650
H	8.076115092609	0.627631107245	2.903059598990
H	8.941821436969	0.693699581416	0.989277650719
H	8.084443807271	0.607189243876	-3.236610325238
H	5.748804601752	-0.024653355899	-2.672086321039
N	5.107594179111	-0.514761148847	1.776553039657
N	6.061282508612	-0.004367703721	-0.621504912125
C	2.919206955565	0.319027966938	-2.679213162582
C	2.389834205917	0.130079354793	-3.950703079780
C	2.466225134899	-1.138429335367	-4.519303237228
C	3.088076919796	-2.164122499922	-3.811641951248
C	3.610196742480	-1.892475527582	-2.548558494136
C	4.350096636631	-2.875713238002	-1.725984972317
C	4.649212053528	-4.169033834735	-2.143702749957
C	5.378177111958	-5.004001394158	-1.300483546005

C	5.792898277558	-4.522993991965	-0.063337490589
C	5.456016060974	-3.222535205129	0.291551068872
C	4.195507879327	2.368651133050	-0.009781689998
C	3.651572784172	3.610977204094	0.294818661616
C	2.404118636809	3.661313183309	0.907034287360
C	1.742509967765	2.470051758286	1.194828033804
C	2.345970549115	1.262328994241	0.858898502233
C	1.739942205949	-0.061978253356	1.102154976481
C	0.536626101716	-0.257124751361	1.773811760984
C	0.061217384331	-1.551834476431	1.956741716528
C	0.796067536269	-2.617506700527	1.447783981220
C	1.995227106226	-2.349507865570	0.798030787537
H	2.877799379622	1.297628993068	-2.195925264642
H	1.923820855998	0.966524592625	-4.474229216314
H	3.165069096011	-3.159477124251	-4.248915508358
H	4.324727921464	-4.528732567487	-3.119981438145
H	6.368853618088	-5.138952303553	0.628790963273
H	5.753103747319	-2.810358991490	1.256738848550
H	5.168730157614	2.290467556835	-0.496231097371
H	4.207300257092	4.517037437780	0.049195819565
H	0.763069567081	2.492129475062	1.673017656547
H	-0.015769685732	0.594065393605	2.168370404097
H	0.460613016440	-3.649822763635	1.556854088812
H	2.604925140502	-3.164300769245	0.406265132015
N	3.502383980896	-0.669228872491	-1.997420718047
N	4.750331496510	-2.425702988837	-0.516588845089
N	3.557193912334	1.226259495039	0.262634602283
N	2.458748084307	-1.105135408485	0.639773059081
H	9.721791956309	0.986388294166	-1.351761359663
H	6.980279160323	0.163648261719	5.083953322125
H	-0.873413816350	-1.727763703050	2.494617060491
H	1.942617636799	4.618325129455	1.159878464965
H	2.052104521036	-1.331052800119	-5.511339382415
H	5.620769675287	-6.021461917099	-1.614626881265

$[Ru(bpy)_3]^{3+/2+}$

V_{11}(Def2-SV(P)) = -3158.534930053921926
 = (-3158.324716574271861) - (-1579.155880982988037) + (-1579.366094462637875)
V_{22}(Def2-SV(P)) = -3158.534930052838718
 = (-3158.324716574271861) - (-1579.155863825897995) + (-1579.366077304465080)
G(Def2-SV(P)) = -3157.634555606114
E(Def2-SV(P)) = -3158.539031875176079
5 2

Ru	-4.299861001233	0.601235307592	0.077220758899
C	-7.013807055345	-3.157314961610	1.499954950822
C	-2.789111948976	0.989512951560	2.706099955904
C	-2.401729468415	0.693018840971	4.005422906422
C	-2.969468994056	-0.413204105599	4.633552804305
C	-3.919131095244	-1.167455143613	3.951756885238
C	-4.273815843482	-0.806733804881	2.652792652259
C	-5.307418521679	-1.503425017382	1.856436345124
C	-6.041380341121	-2.591531889648	2.320404199124
C	-7.233130238547	-2.618054766500	0.236344264584
C	-6.464966741019	-1.533654252605	-0.166792403230
H	-2.377416380526	1.848244126475	2.172166895192
H	-1.665385133839	1.322101369534	4.508229377098
H	-4.381269265533	-2.026799846215	4.436922699285
H	-5.863879267266	-2.999650168205	3.315139994977
H	-7.986800223469	-3.025961837826	-0.438806027568
H	-6.595132043432	-1.083677362429	-1.152686915788
N	-3.691420043834	0.251815700873	2.044272207608
N	-5.523423282746	-0.996207739503	0.619163278498
C	-4.613030025309	0.453340835626	-2.967157676157
C	-5.152674230177	0.773401121153	-4.205479492875
C	-6.234827742162	1.646409498817	-4.254856095275
C	-6.743268739327	2.162562310613	-3.066673076936
C	-6.157800037551	1.797318542004	-1.856880412692
C	-6.627976812524	2.278142391051	-0.539772891097
C	-7.741015913573	3.097296404388	-0.367123752074
C	-8.110286742564	3.485158745699	0.917476993714
C	-7.358734238624	3.042901480010	2.001925825368
C	-6.262491737502	2.226177662561	1.761757636160

C	-2.668241760430	-1.889095509678	-0.621868866130
C	-1.614836973900	-2.600602907654	-1.178562664725
C	-0.543065107163	-1.892576850594	-1.714167305359
C	-0.555364354887	-0.502368013799	-1.659306309964
C	-1.642085415349	0.152569745647	-1.083894954543
C	-1.754980879461	1.624133759570	-0.974132875527
C	-0.768930330503	2.510559220222	-1.398663734233
C	-0.961342044156	3.878926907183	-1.229374259043
C	-2.139893176914	4.329650148097	-0.644162301545
C	-3.089455456925	3.393295278730	-0.254948903014
H	-3.768326936563	-0.232575084475	-2.885445515099
H	-4.722865446433	0.339554815472	-5.109520662418
H	-7.590187164370	2.847763370916	-3.090395365930
H	-8.325980079142	3.430400924237	-1.224128848583
H	-7.611048438864	3.320235782112	3.026474954440
H	-5.643836635115	1.858224091330	2.581923024751
H	-3.535986003434	-2.403615097111	-0.206441495181
H	-1.646331591769	-3.690887300683	-1.191923872958
H	0.273397245844	0.064373217426	-2.079992945135
H	0.148303091324	2.147340142889	-1.859790403300
H	-2.334425720746	5.391936166137	-0.489740888421
H	-4.030625248957	3.700471610052	0.205376168777
N	-5.100350304350	0.952505652789	-1.823620818409
N	-5.904684251880	1.856885545577	0.524429268940
N	-2.686078955584	-0.549197946343	-0.585716031918
N	-2.900064839305	2.077619631456	-0.412865414690
H	-7.596294368452	-4.011682485765	1.851195945153
H	-2.683388767370	-0.684698894663	5.651895144780
H	-0.192134813772	4.583054079452	-1.553851745802
H	0.296939545747	-2.418698887572	-2.172704063809
H	-6.685069214619	1.925818560010	-5.209796145760
H	-8.981754629302	4.126473110513	1.065730416574
Ru	4.306571056001	-0.591551261419	-0.061732799135
C	8.883132822695	0.655029332445	-1.079678820214
C	4.616328311955	-0.675752135943	2.987574142820
C	5.265367904175	-0.508245249055	4.203192079132

C	6.593185379969	-0.092809269668	4.197583996622
C	7.226423355887	0.134826108304	2.979368832404
C	6.518200770789	-0.054449141539	1.795054291624
C	7.099014695842	0.150632160222	0.450398969298
C	8.428248101657	0.501262910806	0.226635895790
C	7.998285037353	0.452229523902	-2.134358240573
C	6.686424276330	0.104692450546	-1.843343870877
H	3.577139015945	-1.006173542849	2.948923979189
H	4.728253944254	-0.703302478348	5.132562789911
H	8.266515671671	0.459280757195	2.960148416190
H	9.112580093363	0.650990970052	1.061320301807
H	8.310248355939	0.559360417311	-3.174286267651
H	5.957198925401	-0.058849565449	-2.638569156196
N	5.224429426364	-0.453568469909	1.815032388124
N	6.247579104025	-0.037347436297	-0.585384187123
C	2.970615433046	0.197064157008	-2.692758570310
C	2.480160888525	0.008043784288	-3.977210731145
C	2.613472042228	-1.248666985056	-4.562249831202
C	3.249862308234	-2.262619945211	-3.853016071038
C	3.730924163203	-2.002380169379	-2.571001487063
C	4.464734688707	-2.989301216044	-1.748782183226
C	4.764544951303	-4.281994517897	-2.170503299514
C	5.487994871472	-5.122864955600	-1.329140637132
C	5.899931592480	-4.650024575457	-0.087111419469
C	5.566376388824	-3.351293248083	0.273835869542
C	4.209597198807	2.461588353560	0.094782117228
C	3.661227896222	3.702916367736	0.392021476461
C	2.394574639583	3.752433341896	0.965305482178
C	1.719661443688	2.562452197880	1.223782281264
C	2.323925650426	1.351590724860	0.895962071385
C	1.693904944912	0.031545431242	1.117852431922
C	0.468208197947	-0.143665404992	1.756325731411
C	-0.037819029355	-1.427741491236	1.928423033331
C	0.685096561567	-2.511032310009	1.438777587882
C	1.902176086827	-2.269557727166	0.815566080945
H	2.893755319563	1.165519995043	-2.194905199436

H	2.001341048141	0.837295707584	-4.500289969842
H	3.371941532213	-3.247033245185	-4.304168525195
H	4.442417542259	-4.638609644222	-3.148600711503
H	6.471936709754	-5.272314354092	0.602679711982
H	5.860409178882	-2.939337041008	1.241101458999
H	5.198774328840	2.374438226538	-0.359064033908
H	4.227314509189	4.609412829141	0.173012833331
H	0.726897823279	2.588039233306	1.673361426102
H	-0.078182230545	0.717719811271	2.136519045043
H	0.323952193555	-3.535280048694	1.541284419522
H	2.510925221701	-3.090909321001	0.434096261275
N	3.568772095682	-0.784418254276	-2.004073062697
N	4.862273941210	-2.546544957436	-0.532402726279
N	3.554869568845	1.318590485614	0.334465252588
N	2.399317073793	-1.033299372748	0.673043274167
H	9.923505553894	0.928921304367	-1.267638965339
H	7.137528332711	0.053634132853	5.132955533166
H	-0.988705367533	-1.582712347640	2.444109393825
H	1.929043442990	4.709106432575	1.211919535933
H	2.232841011865	-1.439927667708	-5.567772035547
H	5.728672822551	-6.139388348791	-1.647777319154

6. Cartesian coordinates and energies of MESX geometries with metal-metal distances showing maximum electron self-exchange reaction rate constants at the UωB97X-D/Def2-TZVP//Def2-SV(P) level

The MESX geometries by EDEEL with the optimized metal-metal distance and energy components in these geometries are presented below. These energy components are:

$V_{11} = E_{n+m}^{\mathrm{C}} - E_{n}^{\mathrm{D}} + E_{n+1}^{\mathrm{D}}$

$V_{22} = E_{n+m}^{\mathrm{C}} - E_{m}^{\mathrm{A}} + E_{m+1}^{\mathrm{A}}$

In the following, energy values at the UωB97X-D/Def2-SV(P) and UωB97X-D/Def2-TZVP//Def2-SV(P) levels are given in Hartree. Note that the Gibbs energy values are before adding the entropy correction of −4.3 kcal/mol.

$[V(H_2O)_6]^{3+/2+}$

V_{11}(Def2-SV(P)) = -2803.296181942368094

= (-2803.084499945237894) - (-1401.541195367326054) + (-1401.752877364456026)

V_{22}(Def2-SV(P)) = -2803.296181947372133

= (-2803.084499945237894) - (-1401.540827316790001) + (-1401.752509318924012)

G(Def2-SV(P)) = -2803.059031293702

E(Def2-SV(P)) = -2803.304923570243318

V_{11}(Def2-TZVP) = -2804.846078668189875

= (-2804.636443683074049) - (-1402.320906117945015) + (-1402.530541103061068)

V_{22}(Def2-TZVP) = -2804.846077006023734

= (-2804.636443683074049) - (-1402.320562958051141) + (-1402.530196281001054)

E(Def2-TZVP) = -2804.854620017075376

5 6

V	-2.677379515715	-0.066131263419	-0.060739331285
O	-1.096520018153	-1.288518230528	-0.879393916815
O	-2.512685519260	-1.171566058420	1.678413928616
O	-4.159169304948	1.151831711172	0.689070421844
O	-2.806664010854	1.012614692366	-1.817067027713
O	-1.179886209743	1.142047644987	0.676926959424
O	-4.153039188103	-1.287948551848	-0.808393300857
H	-4.100103755466	-2.242169724013	-0.994084236977
H	-1.229829199700	-1.651925865037	-1.777343844724
H	-3.048740672167	-1.960741578710	1.874445792561
H	-4.533108693044	1.887612644956	0.171959732691
H	-3.162802960470	0.641892128383	-2.643950426656

H	-1.302831985215	2.074972148869	0.927402968765
H	-0.849315323605	-2.045315137011	-0.310903081945
H	-2.200803378476	-0.794024318374	2.520168916114
H	-4.324720483227	1.316627507810	1.634084180525
H	-2.291275782716	1.811543643000	-2.025896666495
H	-0.289702422644	0.852933741723	0.972628140721
H	-5.091451646351	-1.028510463431	-0.778606587543
V	3.073515774925	0.134301213719	0.052212524595
O	4.539693208178	0.138692859267	-1.406500294928
O	3.086619099149	-1.918616035489	0.195303597927
O	1.510734888428	0.253439724668	1.555513348301
O	3.037168131195	2.183202858348	-0.009732185067
O	4.649034240142	0.180249684988	1.373382467355
O	1.530319012898	0.049561560599	-1.313983252377
H	1.658809005916	0.178690286283	-2.270660980423
H	4.669859178638	0.884781909895	-2.018808845210
H	2.461442852797	-2.528510286933	-0.234976339910
H	1.661743481443	0.937870364420	2.237632685924
H	2.287773530769	2.722076732977	-0.319824336908
H	4.640966461132	0.001643306092	2.330108511458
H	4.860525148380	-0.672914080142	-1.838678540668
H	3.841813284820	-2.430532419262	0.536208670156
H	1.333370700289	-0.577993334120	2.039123789336
H	3.770200839406	2.770591302536	0.247066804441
H	5.570695051483	0.263496722000	1.070146595442
H	0.648789626273	-0.356875759746	-1.173214309016

$[Cr(H_2O)_6]^{3+/2+}$

V_{11}(Def2-SV(P)) = -3004.139754301838366

= (-3003.930042615800176) - (-1501.962125637149029) + (-1502.171837323186992)

V_{22}(Def2-SV(P)) = -3004.139754307990188

= (-3003.930042615800176) - (-1501.962521647953054) + (-1502.172233340143066)

G(Def2-SV(P)) = -3003.901899362690

E(Def2-SV(P)) = -3004.150332712851196

V_{11}(Def2-TZVP) = -3005.738695083419316

= (-3005.539013585035264) - (-1502.770904864552904) + (-1502.970586362936956)

V_{22}(Def2-TZVP) = -3005.738744723481432

= (-3005.539013585035264) - (-1502.770999265829914) + (-1502.970730404276082)

E(Def2-TZVP) = -3005.748685320941149

5 8

Cr	-2.656543978072	-0.350718209000	0.013503254378
O	-0.925284680509	0.116785005124	-1.342627942814
O	-2.299467515354	-2.312051529692	-0.284646790353
O	-4.266403063062	-0.716221294742	1.317691142331
O	-2.892638335710	1.635377727440	0.227978212610
O	-1.274555866767	-0.321460651181	1.497731224385
O	-4.061075276682	-0.306681581265	-1.449075547048
H	-3.912575190788	-0.410837782545	-2.406572412839
H	-1.129861721049	0.784018723588	-2.027748013614
H	-2.876509644585	-2.867041477789	-0.840898649478
H	-4.602705452683	-0.023810923628	1.915248830066
H	-3.649712957878	2.126719891311	-0.138839675969
H	-1.537108169953	-0.423070724671	2.430528565055
H	-0.601405479988	-0.671913498785	-1.822438312885
H	-1.891922396809	-2.872084686108	0.401376641161
H	-4.409268855747	-1.580846533521	1.743724611009
H	-2.484157063465	2.147055486832	0.949197840904
H	-0.320184612983	-0.552132469023	1.411956721234
H	-4.974195225562	-0.573583511824	-1.235339780838
Cr	2.997397823256	0.467990172602	0.597641083737
O	4.381895116477	1.908784052696	-0.072014699251
O	3.207403838231	-0.699363593849	-1.037613583824
O	1.506933440870	-1.043060465980	1.332832220565
O	2.747503703307	1.599687524294	2.248091355287
O	4.625516799990	-0.376840369035	1.458329178782
O	1.372660927522	1.373348257445	-0.208607833715
H	1.451495435919	2.238330256624	-0.650222204359
H	4.382466540241	2.792444290372	0.339281377676
H	2.827860697207	-0.461115456269	-1.903606708311
H	1.640078055690	-1.341905423304	2.254681684932
H	1.895018378394	2.024075195744	2.455809245474
H	4.721443275070	-1.289366788997	1.786090307427

H	4.619436646222	2.009063415414	-1.011801157245
H	4.011322071059	-1.231118344509	-1.183406603979
H	1.533649804692	-1.851986706320	0.782972239046
H	3.217074703743	1.395714995472	3.077572897713
H	5.505923875065	-0.010477841364	1.254635363178
H	0.569173465677	0.916156691630	-0.554373635679

$[Fe(H_2O)_6]^{3+/2+}$

V_{11}(Def2-SV(P)) = -3442.564059368042763

= (-3442.309547743726398) - (-1721.152055919432996) + (-1721.406567543749134)

V_{22}(Def2-SV(P)) = -3442.564059383635140

= (-3442.309547743726398) - (-1721.152221164654975) + (-1721.406732804563944)

G(Def2-SV(P)) = -3442.330578276707

E(Def2-SV(P)) = -3442.573169202407826

V_{11}(Def2-TZVP) = -3444.200836273666027

= (-3443.945178317925183) - (-1721.974195002464967) + (-1722.229852958206038)

V_{22}(Def2-TZVP) = -3444.200942746221244

= (-3443.945178317925183) - (-1721.974236785948960) + (-1722.230001214245021)

E(Def2-TZVP) = -3444.209800469885067

5 10

Fe	-2.653830416987	-0.020018274619	-0.232500451317
O	-0.954233781851	1.318655403758	-0.619523689262
O	-2.384835693331	-0.893392495909	-2.060145456693
O	-4.176569599305	-1.313925630436	0.239727937952
O	-2.867241117641	0.918327097213	1.566552999828
O	-1.195001514160	-1.251163567918	0.546694838174
O	-4.038015458047	1.266833652308	-1.035920848597
H	-3.902600573269	1.946180685197	-1.721033883289
H	-1.121920638117	2.266161734012	-0.443206467471
H	-2.905435287191	-0.683801999514	-2.856771685114
H	-4.565343567762	-1.385835558401	1.130219409427
H	-3.469966961536	1.665383482584	1.733043327864
H	-1.391808297838	-1.963103599473	1.181784649161
H	-0.631248463381	1.269833812615	-1.542191663032
H	-1.882977394391	-1.713451660730	-2.217724504448
H	-4.412828088171	-2.106184798585	-0.275694695846

H	-2.308253460051	0.776236838423	2.351507132319
H	-0.269889200834	-1.366715289685	0.232175926521
H	-4.996054421549	1.134227556433	-0.915973525266
Fe	3.009327852061	-0.289741128079	0.561198072691
O	4.291095400448	0.965388658293	1.575694871371
O	3.277661478646	0.783319656013	-1.157808902685
O	1.544618681808	-1.603058903953	-0.404370858444
O	2.736159711086	-1.408740851073	2.251042024103
O	4.648197063684	-1.444895075776	0.116311258203
O	1.364015836101	0.863618200894	1.000826438251
H	1.338875564213	1.479305173569	1.754780856984
H	4.393731342892	0.953515041613	2.544223746524
H	2.781101487814	1.581818935293	-1.412062055734
H	1.674017578154	-2.559448209003	-0.244859732589
H	1.960477963386	-1.382087310597	2.839429675102
H	4.764132064208	-2.098707658863	-0.596215884325
H	4.590283064444	1.828114353504	1.237116944110
H	3.997507607586	0.639717266306	-1.798249044820
H	1.484639180927	-1.495187876832	-1.374900105714
H	3.314705394930	-2.145672842457	2.517218971476
H	5.516513993268	-1.257372185674	0.516336439244
H	0.563539584644	1.017767912535	0.447416925871

$[Co(H_2O)_6]^{3+/2+}$

V_{11}(Def2-SV(P)) = -3680.603277256203000

= (-3680.299330647796978) - (-1840.146592891019964) + (-1840.450539499425986)

V_{22}(Def2-SV(P)) = -3680.603277250324027

= (-3680.299330647796978) - (-1840.146903951404965) + (-1840.450850553932014)

G(Def2-SV(P)) = -3680.363843758238

E(Def2-SV(P)) = -3680.612399899715911

V_{11}(Def2-TZVP) = -3682.273694587239788

= (-3681.968069544014725) - (-1840.985395121952934) + (-1841.291020165177997)

V_{22}(Def2-TZVP) = -3682.273706863079497

= (-3681.968069544014725) - (-1840.985531665186045) + (-1841.291168984251044)

E(Def2-TZVP) = -3682.282671299707999

5 8

Co	-2.638734439171	-0.046474449081	-0.060421456864
O	-1.154908603427	0.126287247696	-1.619694872560
O	-2.512000448543	-2.054810644194	-0.081930864851
O	-3.881584924459	-0.192379202411	1.565334937305
O	-2.694206023724	1.971237613076	-0.057092809333
O	-1.114553009080	0.036427768464	1.258292861200
O	-4.160516029623	-0.157130382193	-1.371425939295
H	-4.208080336111	0.239185916305	-2.260446899037
H	-1.005286059729	1.063408127592	-1.860027887152
H	-2.855505936603	-2.610637127187	-0.805622202089
H	-4.474316633775	0.539137416020	1.815975790653
H	-3.374525552041	2.495868247371	-0.518788909125
H	-1.267504514792	-0.189536345208	2.193790981550
H	-1.338092689953	-0.342116878209	-2.458623405688
H	-1.834493875141	-2.554846522290	0.409650782274
H	-4.346358616041	-1.031769589477	1.736070244861
H	-2.336639364138	2.501264452278	0.679399247953
H	-0.242659785450	0.488870825252	1.176619675290
H	-5.024051073312	-0.559849772114	-1.165813314032
Co	2.889404874237	-0.007414044834	0.068011739692
O	4.255197647908	-1.248202110550	-0.814482570937
O	2.770912142746	-1.322256502132	1.596668105054
O	1.457201113190	1.254240829529	1.056905800575
O	2.893634798379	1.386307580645	-1.398650061037
O	4.449298179277	0.878408689977	0.992593434051
O	1.360325702732	-0.929644506370	-0.869224031776
H	1.438327793880	-1.831654425102	-1.228718204900
H	4.321780420565	-1.377656661036	-1.777639505077
H	2.014863863283	-1.923061845418	1.730390313669
H	1.335939991193	2.101589188206	0.582649191354
H	2.486380563157	1.219752977166	-2.269045764852
H	4.520139360856	1.793641535836	1.319689469487
H	4.472201007159	-2.091061816442	-0.375791276965
H	3.226371888867	-1.188338835965	2.448430391390
H	1.633336044419	1.483850361042	1.991168251214
H	3.629337663469	2.015846496606	-1.517196055136

H	5.344203383331	0.497768986874	0.925546653915
H	0.496735643630	-0.550301188397	-1.161950584564

$[Ru(H_2O)_6]^{3+/2+}$

V_{11}(Def2-SV(P)) = -1105.127096592040743

= (-1104.897705759668952) - (-552.447662737883434) + (-552.677053570255111)

V_{22}(Def2-SV(P)) = -1105.127096573187146

= (-1104.897705759668952) - (-552.446731328079522) + (-552.676122141597716)

G(Def2-SV(P)) = -1104.872666924485

E(Def2-SV(P)) = -1105.135776920141097

V_{11}(Def2-TZVP) = -1106.476261377968967

= (-1106.247466132030013) - (-553.126046076230750) + (-553.354841322169818)

V_{22}(Def2-TZVP) = -1106.476088810093188

= (-1106.247466132030013) - (-553.125612457415400) + (-553.354235135478689)

E(Def2-TZVP) = -1106.484637503499016

5 2

Ru	-2.690204144839	0.047992392758	-0.151650344202
O	-1.061261508207	-0.421963935641	-1.487566926934
O	-2.506686716974	-1.984268280026	0.398865059004
O	-4.279196297035	0.541720646070	1.109733178692
O	-2.848850380568	2.076493203697	-0.680044830880
O	-1.180040107770	0.566324679969	1.183953284621
O	-4.197664758531	-0.324159022638	-1.537454055834
H	-4.145913621010	-1.038692829876	-2.198338951537
H	-1.200743533317	-0.170548571132	-2.422373504578
H	-3.174684300424	-2.609058614372	0.060520848695
H	-4.443117737274	1.501951999989	1.177490426189
H	-3.110787761861	2.262384638271	-1.601135731810
H	-1.381173584230	1.006788192572	2.029282632092
H	-0.882431707124	-1.384613172501	-1.482720659280
H	-2.383802410436	-2.183727000573	1.345401524398
H	-4.343725567338	0.155097372349	2.002523792332
H	-2.081763031547	2.641008112458	-0.469968915904
H	-0.360367170673	0.035778577090	1.290551578619
H	-5.111899789222	-0.274078612571	-1.199412411557
Ru	3.075418088635	0.001680422249	0.188591542727

O	4.656503660094	0.723519566421	-0.985337430138
O	3.141314173683	-1.641258967497	-1.105492368665
O	1.481456744668	-0.694245272048	1.486305875166
O	3.001751174924	1.647643609557	1.466425876194
O	4.606520464966	-0.738681616660	1.383284052440
O	1.560367854784	0.908019319117	-0.940708965325
H	1.813754485081	1.416427857181	-1.732121968622
H	4.775069399679	1.687819849205	-1.065325917098
H	2.328423043516	-2.125440319288	-1.339207855851
H	1.661193266481	-0.408023840826	2.405017750748
H	2.381007536334	2.362649553680	1.231905507232
H	4.535958532888	-1.599150371839	1.835476012178
H	4.734866951108	0.340793373523	-1.879877891862
H	3.859341318214	-2.293112364282	-1.003990902881
H	1.361794103957	-1.664096604629	1.519849037653
H	3.826981115821	2.048665718378	1.796595726588
H	5.508331964353	-0.643867445464	1.022818221104
H	0.719933235555	0.440748335779	-1.136207248819

$[Co(NH_3)_6]^{3+/2+}$

V_{11}(Def2-SV(P)) = -3442.695530426099140

= (-3442.509067802054233) - (-1721.257553427130006) + (-1721.444016051175140)

V_{22}(Def2-SV(P)) = -3442.695530512053665

= (-3442.509067802054233) - (-1721.257556486492831) + (-1721.444019196492036)

G(Def2-SV(P)) = -3442.273884660070

E(Def2-SV(P)) = -3442.698011813462017

V_{11}(Def2-TZVP) = -3444.016398017086885

= (-3443.826776513426012) - (-1721.916559940698107) + (-1722.106181444358981)

V_{22}(Def2-TZVP) = -3444.016421179060671

= (-3443.826776513426012) - (-1721.916566206672996) + (-1722.106210872307884)

E(Def2-TZVP) = -3444.018871317946832

5 4

Co	3.568130782296	0.133968334103	0.002149204446
N	3.617333543871	1.701326269849	-1.374941347570
H	3.316282403230	2.599625160293	-0.984380212699
H	3.028573029945	1.542380992439	-2.198785055695

H	4.563461539970	1.862195005565	-1.735783384883
N	2.319183849381	1.215164503764	1.279386666096
H	1.590610173907	0.619920179731	1.686676903688
H	1.820639256778	1.981361680504	0.816382706624
H	2.809907584549	1.638005897611	2.073584166909
N	3.629407008200	-1.439769260307	1.378374391538
H	2.967634713225	-2.194513034887	1.171716717729
H	3.430255722763	-1.149915016724	2.341289363203
H	4.552704032711	-1.884107449980	1.406209566686
N	1.847459053030	-0.615483941510	-0.923426854143
H	1.166734592885	0.119001179127	-1.141943197841
H	1.340448750283	-1.298825759319	-0.352224320477
H	2.043985203055	-1.089813173672	-1.810384172729
N	4.734647046298	-1.035374174270	-1.277955861027
H	4.711168990568	-0.718409564255	-2.252319888105
H	4.427062948481	-2.013065096804	-1.294689510639
H	5.725951133304	-1.062470286129	-1.019511548515
N	5.244876307882	0.959412715041	0.933958817022
H	5.159475426638	1.970444219751	1.078936103776
H	6.113092518886	0.830693184816	0.405012606696
H	5.424110031923	0.557015444048	1.859237248513
Co	-4.102843123670	-0.149064007385	-0.003231324909
N	-3.714779446770	0.984225694779	-1.714196474766
H	-4.579156481812	1.281784175483	-2.177745213643
H	-3.174762569649	0.486804839080	-2.429031947438
H	-3.192790505562	1.844442421903	-1.518888280197
N	-5.359536121910	1.379009303020	0.668772232661
H	-6.353635610750	1.193237596583	0.503530568872
H	-5.170241026795	2.279391876633	0.217297977757
H	-5.274689354826	1.545938335269	1.676435293612
N	-4.594218942081	-1.245484980438	1.701533510598
H	-4.302063669580	-2.226689807644	1.654177240125
H	-5.606437652765	-1.263289010559	1.863599834708
H	-4.184199770832	-0.870063998808	2.562343356252
N	-5.649598740809	-1.083802844048	-1.053999910058
H	-6.476018997770	-0.493902827862	-1.193288369307

H	-5.990642127015	-1.934337914187	-0.595153243704
H	-5.356315126226	-1.372025767756	-1.992842129320
N	-2.797716858041	-1.671501486361	-0.600803868129
H	-2.137674821749	-1.377966295283	-1.327767109862
H	-3.274102231603	-2.497468500453	-0.976846717075
H	-2.219105329482	-2.011584211574	0.173761022846
N	-2.485679811645	0.747414875262	0.975954600111
H	-2.712408803757	1.657669084653	1.388985614713
H	-1.695989832045	0.918645190832	0.344838428201
H	-2.107446342589	0.178738422987	1.739817482979

$[Ru(NH_3)_6]^{3+/2+}$

V_{11}(Def2-SV(P)) = -867.286842991708227

= (-867.097269043261690) - (-433.551101521467444) + (-433.740675469914038)

V_{22}(Def2-SV(P)) = -867.286843058529371

= (-867.097269043261690) - (-433.551272959872335) + (-433.740846975140016)

G(Def2-SV(P)) = -866.864689878055

E(Def2-SV(P)) = -867.289462843897013

V_{11}(Def2-TZVP) = -868.284331261225134

= (-868.096881691556064) - (-434.051244550310230) + (-434.238694119979300)

V_{22}(Def2-TZVP) = -868.284403942727522

= (-868.096881691556064) - (-434.051427240277746) + (-434.238949491449148)

E(Def2-TZVP) = -868.286918899852935

5 2

Ru	3.510336687685	0.088039151964	-0.128177149946
N	4.409701830095	-0.135828840094	-2.067555754803
H	3.788418194111	0.144330579241	-2.833269593943
H	4.676336615073	-1.104211948570	-2.271968731586
H	5.266736324279	0.413638748594	-2.189557898956
N	2.616966798203	1.953490366946	-0.718896234029
H	1.596726233077	1.911701528590	-0.808777131978
H	2.957889529446	2.295282761766	-1.623467466108
H	2.802149642791	2.710056014898	-0.051977323327
N	2.572561998190	0.273096355729	1.794495220449
H	2.154463839649	-0.606539015831	2.115781551373
H	1.809779084212	0.957742683681	1.804639110619

H	3.212396739531	0.555458108951	2.543803563872
N	1.792016059956	-0.975815052388	-0.873589876962
H	1.134732289900	-0.371679582274	-1.376834868196
H	1.237709945045	-1.423221024809	-0.135990243736
H	2.032553463737	-1.724360128993	-1.531378756110
N	4.429361212279	-1.755643441826	0.492821077689
H	5.308398403991	-1.948898449694	0.001879285068
H	3.840550825294	-2.579620082636	0.333413605440
H	4.662846523291	-1.772037052184	1.491039045934
N	5.224060250730	1.162714722920	0.591521974525
H	5.475042884267	1.956812837376	-0.007206149236
H	6.071143168053	0.589398531307	0.658271890495
H	5.087185956529	1.565875172812	1.523839254340
Ru	-3.870724997453	-0.097809582745	0.143869366146
N	-4.310937891071	0.320144129152	-1.921504135199
H	-5.279641080810	0.622112171547	-2.066844609163
H	-4.177108123435	-0.491209075075	-2.533583347767
H	-3.730888859429	1.063767694062	-2.322665769544
N	-4.991332301413	1.641916867457	0.723842312075
H	-6.004933131675	1.537129999081	0.614888503587
H	-4.743169077013	2.466453417582	0.166569584142
H	-4.843063800626	1.916292220574	1.700396883177
N	-3.476161401926	-0.511219165576	2.217685093266
H	-3.106174089836	-1.452276775485	2.385667659350
H	-4.315234721200	-0.441292902658	2.802112239637
H	-2.792759410546	0.129260385358	2.634013539283
N	-5.645941578472	-1.303300906443	0.242800549821
H	-6.413510731252	-0.869342115275	0.765214882352
H	-5.481803399592	-2.204181214312	0.704836213165
H	-6.030662350366	-1.532458908877	-0.679207753978
N	-2.714087989663	-1.806200614549	-0.480034451277
H	-2.025472929644	-1.564953602495	-1.200427545578
H	-3.279840844428	-2.561169346295	-0.880472861095
H	-2.174298076074	-2.238647172931	0.276068247145
N	-2.051406993844	1.066439539992	0.074627941684
H	-2.104694640788	1.941853157777	0.605280061021

H	-1.762595558341	1.335208019530	-0.871328234572
H	-1.254095861192	0.554140883317	0.465926352954

$[Ru(NH_3)_5py]^{3+/2+}$

V_{11} (Def2-SV(P)) = -1250.346425422224684

= (-1250.150762654413029)

- (-625.074001162632953) + (-625.269663930444608)

V_{22} (Def2-SV(P)) = -1250.346425431283478

= (-1250.150762654413029)

- (-625.073952937621698) + (-625.269615714492147)

G(Def2-SV(P)) = -1249.822084557713

E(Def2-SV(P)) = -1250.349471865552005

V_{11}(Def2-TZVP) = -1251.733793803222397

= (-1251.540954293549021)

- (-625.769983634622236) + (-625.962823144295612)

V_{22}(Def2-TZVP) = -1251.733818964411512

= (-1251.540954293549021)

- (-625.769927880964019) + (-625.962792551826510)

E(Def2-TZVP) = -1251.736821005446927

5 2

Ru	-4.529621865167	-0.082186080665	0.018152907471
N	-4.075938379049	-1.486295993263	-1.546335851749
H	-4.374358860951	-2.441848327617	-1.324865468749
H	-3.071663290989	-1.563189180801	-1.735980515575
H	-4.518920011245	-1.272525690409	-2.445260883703
N	-6.507294938828	-0.910569072526	0.149089136160
H	-6.891633488787	-0.901036155467	1.099236829786
H	-6.552130530797	-1.888335128800	-0.155858093051
H	-7.188246302703	-0.418531436468	-0.438122320336
N	-3.859898435842	-1.530646447980	1.459680388015
H	-2.837709005618	-1.591585580812	1.510920714412
H	-4.172410602467	-2.483743453253	1.247747718098
H	-4.181036792676	-1.353560741530	2.416974689340
N	-5.040456710050	1.298664766704	1.582526009360
H	-4.492625795269	2.165068557169	1.541512925985
H	-4.918977981128	0.933763938611	2.532542117172

H	-6.020320280980	1.596850619943	1.535405455310
N	-5.235367133353	1.364697240626	-1.408425181757
H	-5.405771043880	0.991458137153	-2.347800344630
H	-4.576258715092	2.140943693807	-1.530268126045
H	-6.116696988034	1.808885891682	-1.129988794199
C	-0.010031135920	1.822808481656	-0.277058430736
C	-0.589412815088	1.552070481585	0.960137034103
C	-1.866994158397	1.010631021953	1.000594208723
N	-2.577695971943	0.731161666360	-0.109257713888
C	-2.009802394629	0.992516555396	-1.303164601758
C	-0.737165815177	1.532110260322	-1.429099270553
H	0.992623177689	2.252732089964	-0.341908227615
H	-0.064034840136	1.757134755304	1.894617137836
H	-2.339341871760	0.797617648578	1.961386940895
H	-2.597352992230	0.764876304071	-2.195480043215
H	-0.331196485291	1.720436849219	-2.424492002443
Ru	4.983064845083	0.094461950995	0.032318241982
N	6.985744089260	0.867560167861	0.189541122328
H	7.676609643249	0.310549416370	-0.323690921619
H	7.324709857561	0.908300570111	1.156105492518
H	7.083480436554	1.818765916633	-0.180721234641
N	5.602192746004	-1.219943869826	-1.555139401075
H	6.555974015971	-1.575492687803	-1.434434488334
H	5.595673861546	-0.786394135965	-2.483784317435
H	5.013746321869	-2.056622612268	-1.627559931296
N	5.493498435518	-1.442284930091	1.447274473957
H	6.397025018420	-1.880452291019	1.240182279533
H	4.815885335700	-2.212051775809	1.449170606610
H	5.567503706678	-1.125969665376	2.419518362451
N	4.395215784736	1.430648896655	1.609454621352
H	4.910441460773	2.316537665058	1.587360814073
H	4.535654798618	1.063782666471	2.555805606471
H	3.405068381036	1.692274036841	1.554402103279
N	4.523021703473	1.639716004159	-1.392781241506
H	5.175360114610	1.671480274292	-2.183024645951
H	4.539241710826	2.580063864068	-0.985227373136

H	3.589998633673	1.546491148730	-1.806413378700
C	0.468181750205	-1.814900446338	-0.272785100352
C	1.180382470533	-1.487942874832	-1.424147690680
C	2.442762448293	-0.925212537108	-1.295992775691
N	3.016149467579	-0.678546047830	-0.102423363300
C	2.313910649703	-0.980131248966	1.006778484993
C	1.046631161978	-1.545736232276	0.965275378115
H	-0.521786258106	-2.272048188522	-0.339890558605
H	0.772252756472	-1.667360709528	-2.420355454475
H	3.019765197145	-0.670165854202	-2.187320732558
H	2.786215583264	-0.765169092045	1.967303686074
H	0.529437583216	-1.771703156124	1.899580645990

$[Co(en)_3]^{3+/2+}$

V_{11}(Def2-SV(P)) = -3906.715092120288318

= (-3906.529144613510198) - (-1953.266233251064023) + (-1953.452180757841916)

V_{22}(Def2-SV(P)) = -3906.715092162918154

= (-3906.529144613510198) - (-1953.266041034662067) + (-1953.451988584070023)

G(Def2-SV(P)) = -3906.061603979986

E(Def2-SV(P)) = -3906.717058701236056

V_{11}(Def2-TZVP) = -3908.475145303697900

= (-3908.290664840852969) - (-1954.147349150026002) + (-1954.331829612871161)

V_{22}(Def2-TZVP) = -3908.475121185414991

= (-3908.290664840852969) - (-1954.147191439431936) + (-1954.331647783993958)

E(Def2-TZVP) = -3908.477128031932352

5 4

Co	-3.873543706532	0.067386073654	-0.012046321930
N	-1.815942542221	0.375343798730	0.283571598888
N	-4.024662139709	1.622943616836	1.376550052233
N	-3.899522238562	1.356531157370	-1.656657569564
N	-3.519668572973	-1.376416163031	-1.480743923474
N	-4.002455393529	-1.372516227172	1.494197378930
N	-5.943770429608	-0.198147450713	-0.084912197046
C	-1.626480939843	1.668452292885	0.972307462286
C	-2.709691771204	1.818584476245	2.022026087209
C	-4.033263425570	0.540721605998	-2.882251530525

C	-3.159907461333	-0.689105791379	-2.739355454426
C	-5.337783422156	-2.003213413314	1.423203566467
C	-6.364084300579	-0.927643760428	1.130301825587
H	-4.654900731273	2.046453597226	-1.617528890386
H	-3.040625464080	1.911123896844	-1.709482850235
H	-4.342369002326	-1.965178212217	-1.636600843073
H	-2.777424849282	-2.030955939808	-1.218151468475
H	-4.295686934295	2.479977341827	0.885314589295
H	-4.747681090530	1.495407239134	2.089550837268
H	-1.247390297854	0.347679066955	-0.566553604706
H	-1.443755813108	-0.378421835366	0.869642113285
H	-3.867563720675	-0.954310199332	2.418980730848
H	-3.278694676456	-2.092836458217	1.425323531054
H	-6.229009924320	-0.726025839619	-0.914449092235
H	-6.452941596491	0.687460845238	-0.154479465596
H	-7.367592135176	-1.364795229932	1.008933884987
H	-6.416910837459	-0.209962049790	1.963932042108
H	-5.581094831256	-2.534094921042	2.356976046430
H	-5.321021239365	-2.748846505471	0.612956286820
H	-1.702608389406	2.473945879129	0.225108327737
H	-0.626382776695	1.739859416568	1.428784976611
H	-2.591830471703	1.051118939373	2.803378384095
H	-2.645372040667	2.802073693702	2.513437795576
H	-2.098045630485	-0.401466166686	-2.685747366897
H	-3.275928441773	-1.355787885888	-3.608191876480
H	-5.089987228609	0.249675968703	-2.988557789661
H	-3.756889523945	1.116066520423	-3.779805876797
Co	4.085658187158	-0.073340858675	0.011561261164
N	5.526906633170	-1.316017522968	0.873089367405
N	5.687789123363	0.491108564795	-1.207150174468
N	3.448341812098	-1.595366579043	-1.271947991692
N	2.572281821323	-0.839642730001	1.240945325450
N	4.452483918387	1.505093644501	1.328735251513
N	2.785529746393	1.310355930517	-0.868941562449
C	6.708042181487	-1.365205307125	-0.014269351597
C	6.943455420096	0.017563702030	-0.588343406482

C	2.121980377427	-2.059425437786	-0.813309390375
C	2.138918468185	-2.145299164216	0.699863846416
C	3.370442175442	2.500372181927	1.173844597510
C	3.057548559302	2.648880389847	-0.302141516072
H	3.391352386038	-1.296804671323	-2.249163454944
H	4.103689746826	-2.382407657613	-1.275401007013
H	1.766929434494	-0.210228367359	1.294918530846
H	2.882232045229	-0.947267924653	2.210442610711
H	5.577025263878	0.057495037862	-2.128566705326
H	5.750880971279	1.494470490830	-1.397068331603
H	5.203921870915	-2.265365494141	1.076463454012
H	5.798388527272	-0.932558779449	1.783321672897
H	5.354623953734	1.948878634996	1.135216338375
H	4.510657070730	1.206161485792	2.306021758420
H	1.806090757973	1.061465294831	-0.706944766584
H	2.880193620607	1.337206628047	-1.887907810915
H	2.205876798770	3.330263173987	-0.454267827565
H	3.920420043916	3.080021096953	-0.833363985433
H	3.653478106695	3.471027257051	1.610867597505
H	2.487748988318	2.138752356373	1.724100843493
H	6.502897779107	-2.082859523263	-0.824091736255
H	7.599888703083	-1.722295561098	0.524400242784
H	7.223425560145	0.723026729546	0.209851301671
H	7.769295879610	0.001546504818	-1.317039377571
H	2.856024926370	-2.912802730120	1.030992497393
H	1.148112650848	-2.431414607710	1.086636260055
H	1.366365856068	-1.331947460214	-1.149913351046
H	1.860706226403	-3.033083034784	-1.256957915050

$[Ru(en)_3]^{3+/2+}$

V_{11}(Def2-SV(P)) = -1331.298470528166945

= (-1331.106771267352997) - (-665.554851176932061) + (-665.746550437746123)

V_{22}(Def2-SV(P)) = -1331.298470594789706

= (-1331.106771267352997) - (-665.554921966882603) + (-665.746621294319311)

G(Def2-SV(P)) = -1330.644518708757

E(Def2-SV(P)) = -1331.300539360303901

V_{11}(Def2-TZVP) = -1332.741915561368842

= (-1332.553952541867829) - (-666.278843392006479) + (-666.466806411507605)

V_{22}(Def2-TZVP) = -1332.741942650165583

= (-1332.553952541867829) - (-666.278888471583969) + (-666.466878579881723)

E(Def2-TZVP) = -1332.743972044019984

5 2

Ru	-3.907936323779	0.039055352007	-0.026228322528
N	-1.834824123971	0.526086977522	0.313186582908
N	-4.157512586235	1.551327422462	1.481114143504
N	-3.939935821254	1.412877167035	-1.679827294677
N	-3.486293322882	-1.336956303141	-1.618742434390
N	-3.993917880556	-1.468243474147	1.502855947583
N	-5.994963549957	-0.444737880593	-0.145399655442
C	-1.756553191819	1.792080544133	1.081350029710
C	-2.849212061637	1.808437604999	2.131758995716
C	-4.022116187510	0.639583606655	-2.943286025797
C	-3.119002009643	-0.572224592140	-2.836054960039
C	-5.271067057513	-2.211997105312	1.373180224140
C	-6.381134372770	-1.232068813989	1.051615360316
H	-4.724982730052	2.069128062299	-1.635163307051
H	-3.108474145946	2.010506140696	-1.702749985991
H	-4.281519224724	-1.946384691472	-1.830392049664
H	-2.726455045269	-1.980161334515	-1.379207493360
H	-4.496038450222	2.413210892505	1.042087802750
H	-4.856964947117	1.341000474629	2.197790283891
H	-1.262776200343	0.609383599340	-0.530835370788
H	-1.387616981710	-0.224608274336	0.848235019009
H	-3.928918452907	-1.066921818971	2.442949594256
H	-3.213515490517	-2.129706073822	1.452688783919
H	-6.221478496333	-0.982089450224	-0.987529641130
H	-6.585414653722	0.389817876695	-0.207825428429
H	-7.332778416050	-1.761159261817	0.886292778108
H	-6.531807608322	-0.531269165732	1.887165724278
H	-5.498235961875	-2.771728685538	2.294063077718
H	-5.156609756066	-2.942231403393	0.557114072682
H	-1.892056559074	2.627007144254	0.376205984081

H	-0.766303933162	1.910742701162	1.549081775527
H	-2.676522452962	1.014604661370	2.875714180932
H	-2.855714262349	2.770210631431	2.668292245549
H	-2.067085669128	-0.260921549434	-2.740186120582
H	-3.198576298551	-1.200479068859	-3.736881788958
H	-5.068474844685	0.326146507120	-3.082821116623
H	-3.740973090552	1.261850163049	-3.807386069388
Ru	4.108092123772	-0.037627939853	0.024450504861
N	5.565649046269	-1.400975105064	0.835847481459
N	5.722737199320	0.320487581292	-1.352384895270
N	3.241856467902	-1.647004841096	-1.106099127643
N	2.543291094279	-0.606939557952	1.384779795255
N	4.740215072040	1.637108863626	1.210362214505
N	2.824100156973	1.468869446792	-0.810528543095
C	6.664326474292	-1.579406020716	-0.144316670678
C	6.974153864867	-0.246318139254	-0.793339953377
C	1.916632012033	-1.985687220134	-0.529823181662
C	2.003500773342	-1.929098486203	0.981924177619
C	3.734177961240	2.719115569794	1.079266635206
C	3.295013490349	2.805165412777	-0.368280523036
H	3.125686257848	-1.420706273292	-2.098248164832
H	3.836333558769	-2.480896088036	-1.104059271740
H	1.779137556204	0.074812362448	1.409579176613
H	2.871164112942	-0.657730752132	2.353856892991
H	5.519271911050	-0.137402330878	-2.246330202709
H	5.876498936405	1.301334244905	-1.600454639093
H	5.201589759735	-2.318789078865	1.105640512802
H	5.947226247674	-1.013057474554	1.704375907343
H	5.658293423747	1.994816546206	0.931254000794
H	4.849521040931	1.396091343445	2.199799632993
H	1.844773768393	1.348607375306	-0.536025241282
H	2.806329434645	1.440881029772	-1.834008963579
H	2.506580857216	3.563516647099	-0.493880662292
H	4.142236380792	3.095452974811	-1.008889587893
H	4.143372058861	3.682546354737	1.421540892825
H	2.880362021943	2.468892053473	1.728063397205

H	6.330401257932	-2.303444026014	-0.903741392711
H	7.562932616864	-1.994805134555	0.338156636019
H	7.369546053895	0.462050360884	-0.048610452632
H	7.739202562799	-0.365403788141	-1.576749941741
H	2.685716332287	-2.705934270400	1.360457634793
H	1.015815811745	-2.106881796831	1.435417771077
H	1.186550761418	-1.248757624032	-0.898903018351
H	1.584873286007	-2.981228067724	-0.864432453637

$[Fe(bpy)_3]^{3+/2+}$

V_{11}(Def2-SV(P)) = -5495.854390301957210

= (-5495.652192927709621) - (-2747.820811284861065) + (-2748.023008659108655)

V_{22}(Def2-SV(P)) = -5495.854390298880389

= (-5495.652192927709621) - (-2747.820811256640809) + (-2748.023008627812033)

G(Def2-SV(P)) = -5494.953500696567

E(Def2-SV(P)) = -5495.857549938864395

V_{11}(Def2-TZVP) = -5499.412442484848725

= (-5499.212315342012516) - (-2749.601942292520107) + (-2749.802069435355861)

V_{22}(Def2-TZVP) = -5499.412444308662089

= (-5499.212315342012516) - (-2749.601929150865999) + (-2749.802058117515116)

E(Def2-TZVP) = -5499.415565427758338

5 2

Fe	-4.267531369281	0.669611604322	0.073403486406
C	-6.990848122693	-2.959653835572	1.484932488315
C	-2.619113216235	1.054461437878	2.526226479711
C	-2.154581613567	0.729788437671	3.794374586493
C	-2.710735047094	-0.366314181560	4.447639731847
C	-3.720021276377	-1.089175517559	3.818237148706
C	-4.143797174410	-0.698425094560	2.550633395541
C	-5.226241621005	-1.362930041961	1.795730116796
C	-5.984887378426	-2.422492955666	2.284262173311
C	-7.212482280777	-2.421477565449	0.221434228948
C	-6.415537877960	-1.364887611355	-0.201542386928
H	-2.206666139838	1.906366549891	1.983043404757
H	-1.367950551742	1.331765358896	4.252239982998
H	-4.169625304038	-1.947873166517	4.316604281322

H	-5.802154941875	-2.827723167916	3.279374715137
H	-7.990636814798	-2.808008506779	-0.438461664429
H	-6.553815761628	-0.921248307960	-1.188702867310
N	-3.586262344367	0.356807451824	1.919688890371
N	-5.445799833893	-0.853462362604	0.564064866719
C	-4.584277628985	0.484801914894	-2.886525186991
C	-5.146958087713	0.807349289057	-4.114891528428
C	-6.231393676149	1.678147018377	-4.143486934864
C	-6.717606603562	2.193185232613	-2.944964169000
C	-6.105068125839	1.823570349626	-1.750886327045
C	-6.534434037497	2.295655180373	-0.418892060271
C	-7.646532734079	3.102911894069	-0.197236730494
C	-7.960915670618	3.479920544056	1.105545712569
C	-7.156601814135	3.037465948874	2.150884077215
C	-6.063422544467	2.233005343647	1.855381663236
C	-2.749427912423	-1.805221934240	-0.633043633663
C	-1.669691488748	-2.521482082187	-1.135594914722
C	-0.561951629707	-1.819890894479	-1.600147592142
C	-0.564628404844	-0.430098682608	-1.526895951864
C	-1.677724563121	0.221379603464	-1.002906023356
C	-1.792153927513	1.688052754162	-0.872896997540
C	-0.789037822630	2.582842490828	-1.234287168163
C	-1.003747810689	3.947336163217	-1.058279536987
C	-2.217958453104	4.378873937622	-0.534092311355
C	-3.174981972737	3.428417072059	-0.199154173044
H	-3.737436637969	-0.200245302163	-2.827730637283
H	-4.732808854138	0.376172536483	-5.027566895837
H	-7.564727756713	2.878871259019	-2.948582984921
H	-8.270377200580	3.434066225040	-1.027310777185
H	-7.364434731624	3.305801411367	3.187792951225
H	-5.408376951984	1.871272582145	2.649172641351
H	-3.642536455902	-2.322403066055	-0.279647498242
H	-1.711712543258	-3.611263177350	-1.164178251966
H	0.292968728524	0.138673740881	-1.883944658144
H	0.155256062140	2.227555747741	-1.647864333146
H	-2.432400138504	5.437467300959	-0.380187236890

H	-4.136248466787	3.728557412730	0.221284802155
N	-5.051088096896	0.979135903359	-1.734479871076
N	-5.758056272057	1.875246497499	0.603137704690
N	-2.757227430729	-0.468349250761	-0.577092874281
N	-2.964592848712	2.117438033176	-0.358174542542
H	-7.596670320908	-3.790923277734	1.851929041302
H	-2.367105852509	-0.657940927376	5.442471911024
H	-0.225334376616	4.663382655027	-1.330660171475
H	0.301477140497	-2.347325459996	-2.012368811694
H	-6.699697724875	1.957548762396	-5.089769904185
H	-8.830339882206	4.112289249582	1.297957422636
Fe	4.698335057406	-0.737404274381	-0.081192657219
C	9.036217503065	0.782409450937	-1.293594958253
C	5.007346170100	-0.845772382024	2.881311370292
C	5.666532602003	-0.639123541143	4.086505836139
C	6.961909493991	-0.132638905801	4.059614171000
C	7.554249593662	0.146472467259	2.831023239337
C	6.834073657653	-0.087824941748	1.663077834150
C	7.356974952259	0.157716427205	0.303736357024
C	8.651929043113	0.589178921854	0.030490126016
C	8.116484457067	0.536618591626	-2.308125778580
C	6.842190049619	0.105696722082	-1.961499753525
H	3.994007789760	-1.249470635926	2.864906956032
H	5.162495505078	-0.876247006529	5.024631689201
H	8.567938561808	0.544860882394	2.790880626680
H	9.360930787765	0.772294192031	0.837800249307
H	8.372543990443	0.673769943476	-3.359803656331
H	6.093124038536	-0.091948555120	-2.729470779950
N	5.575989122500	-0.577488280334	1.700783293200
N	6.471102432235	-0.073966498233	-0.688926244427
C	3.200223248185	-0.010666472271	-2.551192466888
C	2.636771852228	-0.249717317241	-3.797867324470
C	2.824256889179	-1.497187467600	-4.386121107887
C	3.579775651457	-2.455181104002	-3.716223405595
C	4.123887199842	-2.141079035236	-2.473119862991
C	4.968507694160	-3.058231669643	-1.679654886120

C	5.367483064483	-4.320464308686	-2.108908310677
C	6.173969456609	-5.094413727842	-1.278545737991
C	6.560086155172	-4.584857207227	-0.043075762119
C	6.125134467041	-3.315958019505	0.319913908156
C	4.508289990857	2.235680316661	0.032433851926
C	3.899870728786	3.450854705509	0.323097573724
C	2.628142337134	3.442698099634	0.886703024716
C	2.009872136500	2.221872125900	1.143969519541
C	2.678868750551	1.043873816183	0.823760501125
C	2.122542948999	-0.306952902974	1.039158431200
C	0.877310413846	-0.555873882598	1.610011001031
C	0.446656988491	-1.871225375194	1.755394693103
C	1.269734485219	-2.902312127749	1.314426454845
C	2.504924218164	-2.580582459458	0.764439877261
H	3.076687282333	0.956225965208	-2.060626566918
H	2.060319788352	0.535539418568	-4.289669549930
H	3.739112473138	-3.435874216865	-4.164152203421
H	5.059389285022	-4.702334031624	-3.082157500844
H	7.191406515356	-5.154905905798	0.640228786213
H	6.403359246650	-2.884719368513	1.282984824988
H	5.502203372688	2.203766577582	-0.416989874451
H	4.424473889992	4.382342119393	0.105336733720
H	1.014737538647	2.196458147334	1.589032469787
H	0.247520232992	0.266571001649	1.946710766532
H	0.972303752019	-3.948691251056	1.397501072459
H	3.183450226357	-3.365651363576	0.427745205315
N	3.921384086543	-0.933644930187	-1.904695239263
N	5.347567831780	-2.574633923093	-0.476653164061
N	3.910443306502	1.063184400669	0.269708095428
N	2.924947729904	-1.316539873809	0.639287987994
H	10.048022213421	1.121471472705	-1.526273675480
H	7.512106803541	0.045333928462	4.986088612602
H	-0.526911888446	-2.084570700061	2.203051839964
H	2.115764248192	4.377004084549	1.126321211041
H	2.389590950239	-1.725289083195	-5.361663558322
H	6.496644536814	-6.087252321449	-1.599659283066

$[Co(bpy)_3]^{3+/2+}$

V_{11}(Def2-SV(P)) = -5733.941931071816725

= (-5733.764781818348638) - (-2866.876474973177210) + (-2867.053624226644843)

V_{22}(Def2-SV(P)) = -5733.941931099316207

= (-5733.764781818348638) - (-2866.876344784775938) + (-2867.053494065743962)

G(Def2-SV(P)) = -5733.043265172826

E(Def2-SV(P)) = -5733.945641776352204

V_{11}(Def2-TZVP) = -5737.532969739830151

= (-5737.357104597199395) - (-2868.673892021231950) + (-2868.849757163863160)

V_{22}(Def2-TZVP) = -5737.532969522004350

= (-5737.357104597199395) - (-2868.673790825252127) + (-2868.849655750057082)

E(Def2-TZVP) = -5737.536652273976870

5 2

Co	-4.224691311278	0.561805332032	0.091775640689
C	-6.853847433313	-3.076680484716	1.569082589938
C	-2.649243022282	1.105533957723	2.631136380721
C	-2.192498512033	0.812358795514	3.911089851965
C	-2.708067957756	-0.306726421985	4.559403625783
C	-3.672276445279	-1.080634568994	3.918404866596
C	-4.088385434542	-0.715506523061	2.639899154878
C	-5.136138432224	-1.425475712353	1.871711840157
C	-5.876798988377	-2.492515582269	2.371433185652
C	-7.067527973464	-2.576858748507	0.288952716312
C	-6.290575286697	-1.510688251303	-0.146684265535
H	-2.275112083949	1.974998200611	2.086057331340
H	-1.445579908642	1.453771030424	4.382032394791
H	-4.089860254949	-1.955642079910	4.416525496639
H	-5.703042349442	-2.866308033088	3.380338098959
H	-7.823419641864	-2.999759668956	-0.374393026116
H	-6.420472899565	-1.093667749829	-1.146186192980
N	-3.565213523820	0.355625404360	2.015448483418
N	-5.349280864555	-0.955937591971	0.623010606431
C	-4.520335769235	0.278190504416	-2.933554713073
C	-5.031801286227	0.615997345287	-4.181291937509
C	-6.083119402494	1.524903791235	-4.243173585654

C	-6.590949930964	2.057436116999	-3.060742562678
C	-6.028271684646	1.666782887767	-1.847966903655
C	-6.493351046765	2.152749035042	-0.528542204339
C	-7.620246321543	2.951814044115	-0.353867159996
C	-7.981083776998	3.346987358463	0.931341645217
C	-7.207476858187	2.930627268973	2.009679987510
C	-6.096894898871	2.134005403332	1.761534771876
C	-2.690318821158	-1.910260569373	-0.562757053508
C	-1.619132983870	-2.615659764644	-1.098319748040
C	-0.544189885444	-1.902133254411	-1.618250896862
C	-0.567272780217	-0.511697749676	-1.562418441507
C	-1.668095516102	0.128568270693	-1.000255488197
C	-1.805366956375	1.594466852341	-0.885803180237
C	-0.828257689688	2.502816634652	-1.280184082070
C	-1.059729630378	3.865296025903	-1.109486976265
C	-2.264286810221	4.281337689660	-0.553105444161
C	-3.198112820729	3.318783061721	-0.188120422626
H	-3.699100707345	-0.435568923510	-2.844038648934
H	-4.605638308708	0.169442851352	-5.081068622108
H	-7.412255536487	2.773272971472	-3.093834665001
H	-8.222621651650	3.259986817586	-1.208241086004
H	-7.451702545131	3.212825125589	3.034896310012
H	-5.462637391214	1.791218964911	2.580207373964
H	-3.559112574796	-2.437990262118	-0.167715836905
H	-1.644050504701	-3.706220053756	-1.111397166288
H	0.261533943326	0.064501255125	-1.970341105252
H	0.108801561293	2.160428811337	-1.717741586685
H	-2.491003375642	5.337219000558	-0.398565017583
H	-4.151879696977	3.607521837146	0.255492303671
N	-5.005670725696	0.792303887498	-1.801618126856
N	-5.748567608811	1.760566747655	0.526243786961
N	-2.716609501030	-0.573812883009	-0.523751909321
N	-2.972402865699	2.011133000068	-0.348873343444
H	-7.444133539137	-3.913974818626	1.947508429482
H	-2.369156019044	-0.576132387885	5.562166717166
H	-0.301038835937	4.591151418170	-1.410021212387

H	0.307262424210	-2.420768357029	-2.064690407810
H	-6.509919616449	1.821119309398	-5.203956218620
H	-8.863311525589	3.972401540860	1.084337166806
Co	4.442768231801	-0.612619724468	-0.094050266502
C	8.795729229142	0.934663068629	-1.197095822735
C	4.767003825121	-0.812707390591	2.932631296604
C	5.408042111268	-0.581143865498	4.144294591970
C	6.680052869846	-0.018288413409	4.124839917513
C	7.269002248346	0.286397134607	2.900100419832
C	6.564999276398	0.022063390063	1.727758539819
C	7.093505996495	0.288013940673	0.370189799064
C	8.391988444199	0.723479211282	0.118422568632
C	7.891495314635	0.701962155249	-2.227918055047
C	6.612369361458	0.268227637051	-1.903634597144
H	3.770681530828	-1.258674029679	2.906663118530
H	4.909414716763	-0.840796678163	5.079470524270
H	8.264265369021	0.729536215490	2.869071839159
H	9.091652463579	0.892063677190	0.936849558138
H	8.161911432897	0.850632104068	-3.274395123178
H	5.873930400653	0.080860047685	-2.683975374166
N	5.331639968274	-0.517128854195	1.760098777918
N	6.224005321688	0.073196517372	-0.639502231117
C	3.008905313493	0.225262670955	-2.640570682998
C	2.443140355414	0.008009108748	-3.891651526205
C	2.583040374210	-1.248981182446	-4.473638751510
C	3.297922762491	-2.235006469009	-3.798376741616
C	3.846251528907	-1.936777097309	-2.552592870150
C	4.668279775327	-2.878436079120	-1.759496472513
C	5.037794930657	-4.146041666460	-2.199235266559
C	5.833077615083	-4.944353710231	-1.380998416060
C	6.241379433106	-4.453394575174	-0.145604372796
C	5.832075114698	-3.180436171297	0.232120967382
C	4.248946032816	2.335058078268	0.047709757751
C	3.639352839524	3.540274435476	0.375909027517
C	2.388197709183	3.512260967555	0.982075402663
C	1.788355777500	2.282106068197	1.239067327638

C	2.454417885478	1.115050047929	0.878709502812
C	1.914201201394	-0.243408993277	1.086200621509
C	0.700120719005	-0.515095280831	1.711046124342
C	0.289085454635	-1.837086742940	1.853068115530
C	1.098554753467	-2.851679965137	1.353548107969
C	2.302034581408	-2.507909289712	0.749308143996
H	2.923089116119	1.196337113253	-2.147698824626
H	1.902810027607	0.814202747767	-4.390741501856
H	3.422140596294	-3.221291192501	-4.245300190874
H	4.716804348374	-4.513252536538	-3.173904558092
H	6.867121032991	-5.041078821789	0.527633770525
H	6.120862267913	-2.762481431586	1.197402969960
H	5.227463308881	2.318688985932	-0.434305642812
H	4.148577355140	4.479224281083	0.153993238653
H	0.809178357134	2.240859331729	1.715645374538
H	0.082105897720	0.294358936727	2.096835213357
H	0.815638501231	-3.902391100932	1.431964626940
H	2.966639827259	-3.281261850900	0.363309252555
N	3.681538706290	-0.725683800713	-1.989570322089
N	5.063634383356	-2.418694903716	-0.552304821905
N	3.669223334836	1.155027405362	0.289581350118
N	2.702392563016	-1.237902577174	0.627380525527
H	9.811366979025	1.275208041851	-1.410136557251
H	7.215732524162	0.184498632734	5.054822789688
H	-0.657410423511	-2.069772251804	2.346425749427
H	1.876599929815	4.438014922504	1.254148584249
H	2.144842446089	-1.463218889300	-5.450825169067
H	6.131425855865	-5.941186506252	-1.712929587087

$[Ru(bpy)_3]^{3+/2+}$

V_{11}(Def2-SV(P)) = -3158.535220499114985

= (-3158.325229564693927) - (-1579.156410532812970) + (-1579.366401467234027)

V_{22}(Def2-SV(P)) = -3158.535220504411882

= (-3158.325229564693927) - (-1579.156222449703137) + (-1579.366213389421091)

G(Def2-SV(P)) = -3157.635327461220

E(Def2-SV(P)) = -3158.538960793356182

V_{11}(Def2-TZVP) = -3161.817893313180775

= (-3161.612361913295899) - (-1580.801322201627954) + (-1581.006853601512830)

V_{22}(Def2-TZVP) = -3161.817883725857882

= (-3161.612361913295899) - (-1580.801152322796952) + (-1581.006674135358935)

E(Def2-TZVP) = -3161.821592751013668

5 2

Ru	-4.299861001233	0.601235307592	0.077220758899
C	-7.064955406088	-3.123097565505	1.494611905591
C	-2.768639107954	0.952410584131	2.696245284232
C	-2.375907793895	0.641555555535	3.990844190883
C	-2.967642697405	-0.449746620936	4.622544209657
C	-3.941904277770	-1.178624000125	3.947677233397
C	-4.295882268614	-0.808329103692	2.651412361318
C	-5.336952441617	-1.492028102409	1.853272165836
C	-6.094249855024	-2.562896014758	2.320627629886
C	-7.258558521551	-2.596452225227	0.221384493879
C	-6.468698515887	-1.528741548133	-0.184080429010
H	-2.340561285928	1.803180725499	2.162328223450
H	-1.618258535282	1.249581448225	4.487878393449
H	-4.419679157615	-2.028634154219	4.434182625452
H	-5.936453192097	-2.961016863752	3.322775555847
H	-8.009504674141	-3.000821496760	-0.458922624402
H	-6.580012092350	-1.087942711870	-1.176454268196
N	-3.696132615218	0.240174157910	2.042050783897
N	-5.529939527681	-0.995582038226	0.608112421436
C	-4.584496473792	0.463984330372	-2.968279482247
C	-5.118363243694	0.780520927419	-4.210044984485
C	-6.210527045510	1.640773118811	-4.265790590864
C	-6.733722506965	2.149038739814	-3.080577849765
C	-6.153047033160	1.788117380637	-1.867126148101
C	-6.635952315865	2.262927197297	-0.552259846137
C	-7.755529507758	3.074469394374	-0.385303952530
C	-8.135165874189	3.457841211175	0.897646580204
C	-7.387307176683	3.018672808799	1.985932699851
C	-6.283607318251	2.210241981553	1.751072088578
C	-2.629716686770	-1.872347332722	-0.598194842855

C	-1.550093983198	-2.572213603162	-1.119274556253
C	-0.469116636077	-1.852839697126	-1.620383824480
C	-0.498136134420	-0.463063647247	-1.565433955473
C	-1.609148192468	0.178950075513	-1.022717280155
C	-1.737402787294	1.648441540849	-0.910940832423
C	-0.744567652065	2.544175043792	-1.298977562154
C	-0.955139834340	3.910349209662	-1.134088903048
C	-2.157834197443	4.349318618124	-0.589943510194
C	-3.110728418576	3.403721619467	-0.232962840662
H	-3.731727024539	-0.211259468555	-2.880070053048
H	-4.676492239856	0.354234136291	-5.111873767479
H	-7.588017013813	2.824849644857	-3.109390108013
H	-8.337099064511	3.405329643039	-1.245522766716
H	-7.647895009015	3.292496622557	3.009381331291
H	-5.666710656269	1.845510856333	2.574080511096
H	-3.505847350654	-2.396057821529	-0.212459528329
H	-1.569341613085	-3.662813309068	-1.133096786293
H	0.337239527351	0.115010065023	-1.957052433946
H	0.191302551497	2.188786304534	-1.728428164311
H	-2.367594227091	5.409567734159	-0.441310888941
H	-4.068688703296	3.701507274326	0.197756234632
N	-5.086886098196	0.954707552899	-1.827656782338
N	-5.915855188418	1.845590449450	0.515515941270
N	-2.663015572757	-0.533032247390	-0.561479459679
N	-2.904186973340	2.089875194483	-0.385799697676
H	-7.665924146731	-3.963434821913	1.848582897536
H	-2.679858601348	-0.730212005763	5.638019458107
H	-0.181428303292	4.622182949760	-1.429932382257
H	0.391642618094	-2.368957389304	-2.051036781648
H	-6.656760967473	1.916835967381	-5.223595934323
H	-9.011548074508	4.093416740662	1.041671378967
Ru	4.521731857431	-0.621370925645	-0.065206638085
C	9.068712573278	0.681472844734	-1.143486319764
C	4.867138237736	-0.689138720357	2.978320880746
C	5.529934784136	-0.518706923504	4.186056581341
C	6.856150662653	-0.098481570723	4.164577902393

C	7.473781781614	0.133008687636	2.939017507884
C	6.751905857254	-0.057900656727	1.763319043142
C	7.313075170111	0.154375138868	0.411486268467
C	8.635237577825	0.519100661501	0.169151907595
C	8.170563875713	0.472423668672	-2.185680340636
C	6.867132477063	0.109254055446	-1.876402675192
H	3.828971326115	-1.023972468190	2.951192562073
H	5.004659722832	-0.715556113138	5.121793455510
H	8.512408702724	0.461300268577	2.907288357219
H	9.330325018123	0.674032503412	0.994023064621
H	8.465765838521	0.586840826794	-3.229711875126
H	6.127568544406	-0.061192375586	-2.660661940542
N	5.460830895751	-0.464585933296	1.798891083683
N	6.448879105319	-0.040142043170	-0.612423285677
C	3.109886435309	0.127709589506	-2.667903852855
C	2.589204649161	-0.079994309179	-3.937585629199
C	2.739288522107	-1.335172077453	-4.521591032298
C	3.420063213716	-2.329693521562	-3.826073763217
C	3.927161177438	-2.052083742346	-2.558149447446
C	4.699312472919	-3.020116712217	-1.748592693959
C	5.020243233227	-4.306665920325	-2.173654991238
C	5.775769708913	-5.129919169342	-1.343204360292
C	6.198134967076	-4.646266240418	-0.108844687950
C	5.843550566863	-3.354141256406	0.255500784026
C	4.371782960686	2.428890347699	0.085619408920
C	3.798899074486	3.661860184383	0.370546756839
C	2.528408924451	3.692071667521	0.936911223781
C	1.874076364295	2.491714698240	1.200783779547
C	2.502152887918	1.289717870743	0.884838514217
C	1.895269045253	-0.040215289223	1.112890570894
C	0.667255281235	-0.234479099607	1.741832211076
C	0.184365520929	-1.527137311933	1.916881493506
C	0.932652768826	-2.599182320221	1.440485263617
C	2.150413228228	-2.338835162116	0.826560117647
H	3.023374503159	1.096318348437	-2.172050251759
H	2.074634681764	0.734133559541	-4.450380941576

H	3.554441223633	-3.313039072576	-4.275859488809
H	4.690632196161	-4.671855318377	-3.146130056658
H	6.794099775946	-5.255317560477	0.572407681560
H	6.145834620444	-2.933996634800	1.216690005251
H	5.364703974864	2.356816306487	-0.362671961413
H	4.349367087017	4.576894667791	0.147190762498
H	0.879117747963	2.501725822306	1.646241843244
H	0.098062925446	0.617986775940	2.109216680710
H	0.589974403768	-3.629610027385	1.544692414348
H	2.778154036014	-3.150603267277	0.455436690162
N	3.751776020071	-0.835253426998	-1.992868486915
N	5.108644105860	-2.566534684663	-0.540136230328
N	3.736829343506	1.275698352065	0.330122859159
N	2.625058263887	-1.094085515628	0.680587067251
H	10.103159493848	0.967084148670	-1.346218247837
H	7.411521472653	0.048860731435	5.093323165169
H	-0.769256095764	-1.696742011381	2.421561494213
H	2.044509231012	4.642013682842	1.174050981607
H	2.336640165663	-1.540419433598	-5.515723074070
H	6.033674806256	-6.141380692808	-1.664440377843
